# Burden of type 2 diabetes in working-age adults (20–54 years): a GBD 2021 analysis projecting trends to 2035 and exploring the potential benefits of physical activity

**DOI:** 10.3389/fpubh.2025.1706523

**Published:** 2026-01-05

**Authors:** Qiang Liu, Xiaoyang He, Yameng Liu

**Affiliations:** 1 Department of Basic Studies, Shandong Xiehe University, Jinan, China; 2 Bo’ai County People’s Hospital, Jiaozuo, China; 3 Physical Education Teaching and Research Section, Cangzhou Jiaotong College, Hebei, China

**Keywords:** DALYs (disability adjusted life year), global burden of disease (GBD) 2021, incidence, low physical activity, mortality, type 2 diabetes

## Abstract

**Background:**

Type 2 diabetes (T2D) has risen sharply across all age groups and regions, with growing disability and premature mortality. Evidence on regional disparities and modifiable drivers, particularly low physical activity, is needed to guide targeted prevention.

**Methods:**

Using Global Burden of Disease (GBD) 2021 data spanning 1990 to 2021, we analyzed the incidence, mortality, and disability-adjusted life years (DALYs) of T2D among adults aged 20–54 years. We estimated average and annual percentage changes (EAPCs) along with their 95% uncertainty intervals (UIs), compared the burdens across Socio-demographic Index (SDI) categories and geographic regions, quantified the proportion of burden attributable to low physical activity, and projected future trends through 2035.

**Results:**

From 1990 to 2021, global T2D cases in working-age adults increased by 216% to 13.69 million (95% UI: 12.15–15.47), while mortality rose by 112% to 176,562 deaths (95% UI: 163,156–189,590). DALYs increased by 194% to 22.17 million (95% UI: 17.43–27.93). The burden was higher among men and increased with age. Significant disparities were observed: high-SDI regions had the highest incidence rates, while lower-middle-SDI regions had the highest mortality rates. Oceania, the Caribbean, and Central Latin America showed the highest regional rates. In 2021, low physical activity accounted for 863,921 DALYs (95% UI: 377,518–1,366,461) in this age group. Projections suggest continued increases in T2D burden through 2035.

**Interpretation:**

The T2D burden in working-age adults has more than doubled since 1990, with large inequalities across regions and SDI levels. Physical inactivity is a significant modifiable risk factor. Urgent region-specific strategies integrating physical activity promotion, healthier food environments, and improved primary care are needed to reduce future burden and inequities.

## Introduction

Type 2 diabetes (T2D) has emerged as a fast-growing global health crisis, reaching epidemic proportions over the past few decades. The number of adults living with diabetes worldwide has more than quadrupled since 1990, now exceeding 800 million, driven overwhelmingly by T2D. This rise corresponds to a doubling in global diabetes prevalence among adults from about 7% in 1990 to roughly 14% by 2022 (1). An estimated 529 million individuals of all ages were living with diabetes in 2021, more than 95% of whom had T2D (2). While T2D historically affected mostly older adults, it is increasingly being diagnosed in younger age groups, amplifying the lifetime disease burden (3). Millions of new cases occur each year, and the incidence is rising most steeply in middle-aged and even young populations in developing regions (4). This explosive growth of T2D across all age groups underscores its status as one of the defining public health challenges of our time.

Diabetes is a leading cause of morbidity and premature mortality worldwide, fueled largely by T2D and its complications. Globally, diabetes was the eighth leading cause of death and disability (DALYs) in 2019 (5). That year alone, diabetes was responsible for approximately 1.6 million deaths, reflecting a nearly 70% increase in diabetes-related mortality since 1990 (6). By 2022, diabetes had become one of the top ten causes of death globally, with particularly sharp increases in death rates seen in South Asia and sub-Saharan Africa (7, 8). The cumulative disability burden from T2D, including years lived with complications such as cardiovascular disease, kidney failure, blindness, and amputations, has likewise mounted relentlessly (9, 10). In economic terms, the global burden of diabetes is enormous and growing. Annual health expenditures on diabetes were estimated at $966 billion USD in 2021 (11), and total economic costs (including indirect losses from reduced productivity) are projected to soar from about $1.3 trillion in 2015 to $2.2–2.5 trillion by 2030, exceeding 2% of the world’s GDP (12). Even if the ambitious global targets for non-communicable disease (NCD) control are met, diabetes will continue to place significant strain on healthcare systems and economies. These alarming figures underscore the urgent need to curb the escalating burden of diabetes and its widespread societal impact.

Among the modifiable drivers of the T2D epidemic, physical inactivity has been identified as a key risk factor for both the development of T2D and its adverse outcomes. Insufficient physical activity contributes substantially to obesity, insulin resistance, and poor glycemic control, creating a clear pathway to diabetes onset and progression (13). Epidemiological studies indicate that failing to meet basic physical activity guidelines is associated with significantly higher risk of T2D; indeed, one analysis estimated that around 7% of global T2D cases can be directly attributed to lack of physical activity (14, 15). Sedentary lifestyles are thought to be a major cause of the rising diabetes prevalence in urban populations worldwide (16). As a result, global health authorities consider physical inactivity one of the leading preventable risk factors for T2D and related NCDs, on par with unhealthy diet and smoking, and have called for urgent action to promote exercise at the population level. Together with dietary improvements, increasing physical activity represents a cornerstone of strategies to combat the diabetes epidemic and its downstream health effects.

In this study, utilizing estimates from GBD 2021, we quantified the incidence, mortality, DALYs, and the proportion attributable to low physical activity of T2D among working-age adults (20–54 years) across different regions. Such updated and in-depth analyses are critical for formulating region-specific public health strategies. Robust epidemiological evidence serves as the cornerstone for effective responses—including both the implementation of targeted preventive interventions (such as promoting physical activity) in high-burden areas and the rational allocation of medical resources in regions most in need of diabetes treatment and complication management. In summary, the global burden of T2D continues to increase at an alarming rate, urgently necessitating reinforced monitoring and action.

## Methods

### Overview and data collection

The GBD study provides estimates of disease burden for 371 diseases and injuries and 88 risk factors across 204 countries and territories (17). For further details regarding the locations, diseases, and risk hierarchies used in GBD 2021, please refer to the Global Health Data Exchange (GHDx). Within the GBD framework, a comparative assessment of disease occurrence rates and mortality can be conducted across different countries, regions, and globally. The GBD analysis employs three key metrics to quantify disease burden: mortality, incidence, and DALYs. DALYs are calculated as the sum of years of life lost (YLL) Equation 1 due to premature death and years lived with disability (YLD) Equation 2 (18). The specific formulas used are as follows:


$$\begin{array}{l} \mathrm{YLL}=\text{Number of deaths}\times \text{Standard life}\;\\ \text{expectancy}\;\mathrm{at}\;\mathrm{age}\;\text{of death} \end{array}$$ (1)

YLD=Disease prevalence×Disability weight (2)

This study retrieved and analyzed data on the number of cases, incidence rate, mortality, and DALYs of T2D among adults aged 20–54 years from 1990 to 2021, as well as the burden attributable to low physical activity. The data were sourced from the GBD database,^1^ covering 204 countries and regions.

The GBD data utilized in our analysis were downloaded on July 3, 2025. The analysis encompassed multiple dimensions, including sex, age (categorized into 20–24, 25–29, 30–34, 35–39, 40–44, 45–49, and 50–54 years), and geographic location. However, analyses related to race and ethnicity were not conducted, as these parameters were not available in the GBD database. This cross-sectional study involved the analysis and description of disease data over time and across regions and did not include any identifiable personal information. The study was conducted in strict accordance with the Strengthening the Reporting of Observational Studies in Epidemiology (STROBE) guidelines (19).

### Sociodemographic index

The Socio-demographic Index (SDI) is a comprehensive metric for assessing the socioeconomic development of a country or region (20). This index encompasses multiple dimensions, including but not limited to economic structure and scale, educational attainment, living standards, as well as social welfare and security. The SDI value ranges from 0 to 1, with higher values indicating greater levels of socioeconomic development. According to the GBD database, countries and regions are categorized into five groups based on their SDI values: low, low-middle, middle, high-middle, and high. This classification facilitates the investigation of the impact of socioeconomic indices and geographical disparities on the burden of T2D among adults.

#### Case definition and diagnostic guidelines context

GBD case estimation harmonizes multiple diagnostic metrics for diabetes (fasting plasma glucose, 2-h OGTT, and, where adopted, A1C ≥ 6.5%) using standardized models. Because A1C-based diagnosis was adopted at different times across regions, secular increases in case ascertainment may reflect true increases and changes in diagnostic practice.

### Auto regressive integrated moving average

The Autoregressive Integrated Moving Average (ARIMA) model combines the Autoregressive (AR) model and the Moving Average (MA) model (21). Its fundamental assumption is that the time series data constitute a time-varying random variable, whose autocorrelation can be captured by the ARIMA model, enabling the prediction of future values based on past observations. The model is represented by the equation:


$$\begin{array}{l} Y_{t}=\varphi_{1}Y_{\left\{t-1\right\}}+\varphi_{2}Y_{\left\{t-2\right\}}+\ldots +\varphi_{p}Y_{\left\{t-p\right\}}\\ +e_{t}-\theta_{1}e_{\left\{t-1\right\}}-\theta_{2}e_{\left\{t-2\right\}}-\ldots -\theta_{q}e_{\left\{t-q\right\}} \end{array}$$

where:

*φ*_1_*Y*_*t* − 1_ + *φ*_2_*Y*_*t* − 2_ + … + *φ_p_Y*_*t*–*p*_ + *e_t_* denotes the autoregressive (AR) component;

*e_t_* − *θ*_1_*e*_*t* − 1_ − *θ*_2_*e*_*t* − 2_ − … − *θ_q_e*_*t* − *q*_ represents the moving average (MA) component;

*Y*_*t*–*p*_ is the observed value at time period t − p;

*p* and *q* are the orders of the AR and MA parts, respectively;

e*_t_* refers to the random error term at time *t.*

### Statistical analysis

Based on the statistical data from the GBD database, the incidence rate, mortality rate, and DALY rate per 100,000 population, along with their corresponding 95% uncertainty intervals (UI), were calculated. The annual percentage change (APC) and its 95% confidence interval (CI) were estimated using a Joinpoint regression model to evaluate temporal trends within each distinct period (22). This approach provides a detailed understanding of annual fluctuations in rates, offering a fine-grained perspective on year-to-year variations. A log-transformed linear regression model was applied to analyze temporal trends in the incidence, mortality, and survival duration related to T2D among adults aged 20–54 from 1990 to 2021. The estimated average annual percentage change (EAPC) was also computed. The EAPC is particularly valuable for assessing long-term trends, as it reveals whether the rates have increased or decreased over time, independent of short-term fluctuations (23). An EAPC value with a lower bound of the 95% CI greater than 0 indicates an upward trend in the corresponding metric. Conversely, an EAPC value with an upper bound of the 95% CI less than 0 suggests a declining trend. The relationship between disease burden indicators and the Socio-demographic Index (SDI) was analyzed using fitted curves. All analyses in this study were conducted using R software version 4.4.2, with a significance level set at *p* < 0.05.

## Results

### Global tends

#### Incidence

**Figure 1:**
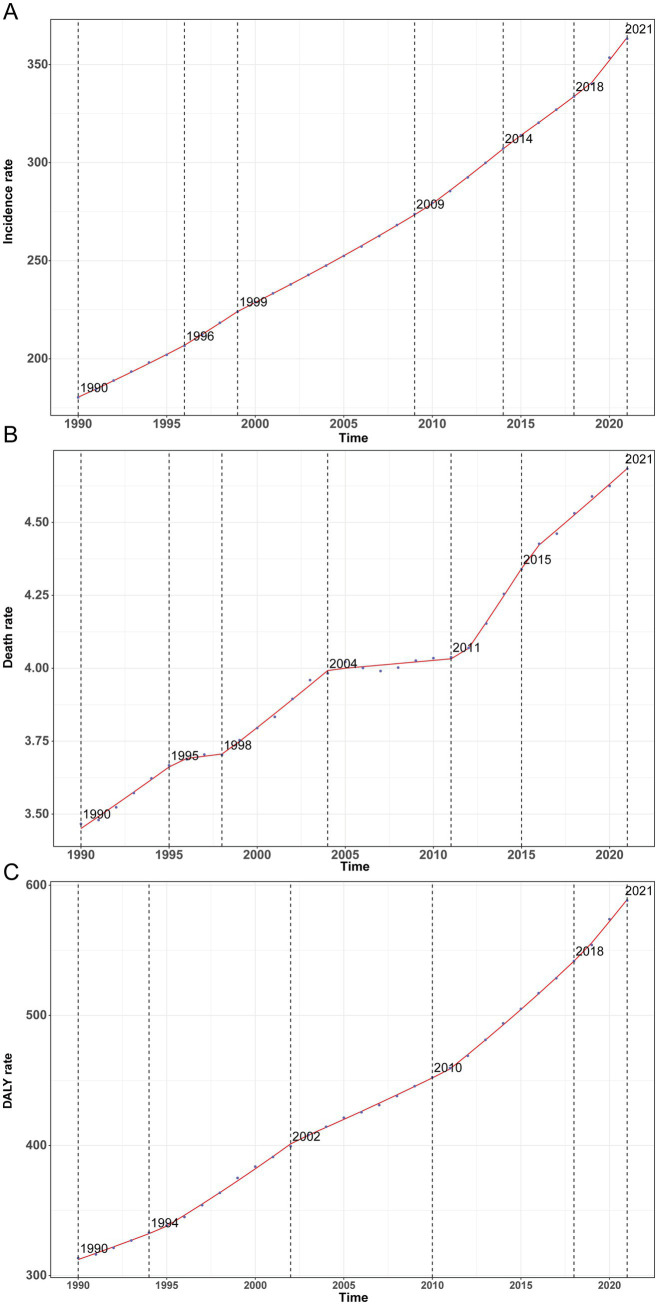
Annual percent change (APC) and trends in incidence, mortality, and disability-adjusted life years (DALYs) attributable to type 2 diabetes among adults aged 20–54 years worldwide from 1990 to 2021. **(A)** Incidence rate. **(B)** Mortality rate. **(C)** DALY rate.

**Figure 2:**
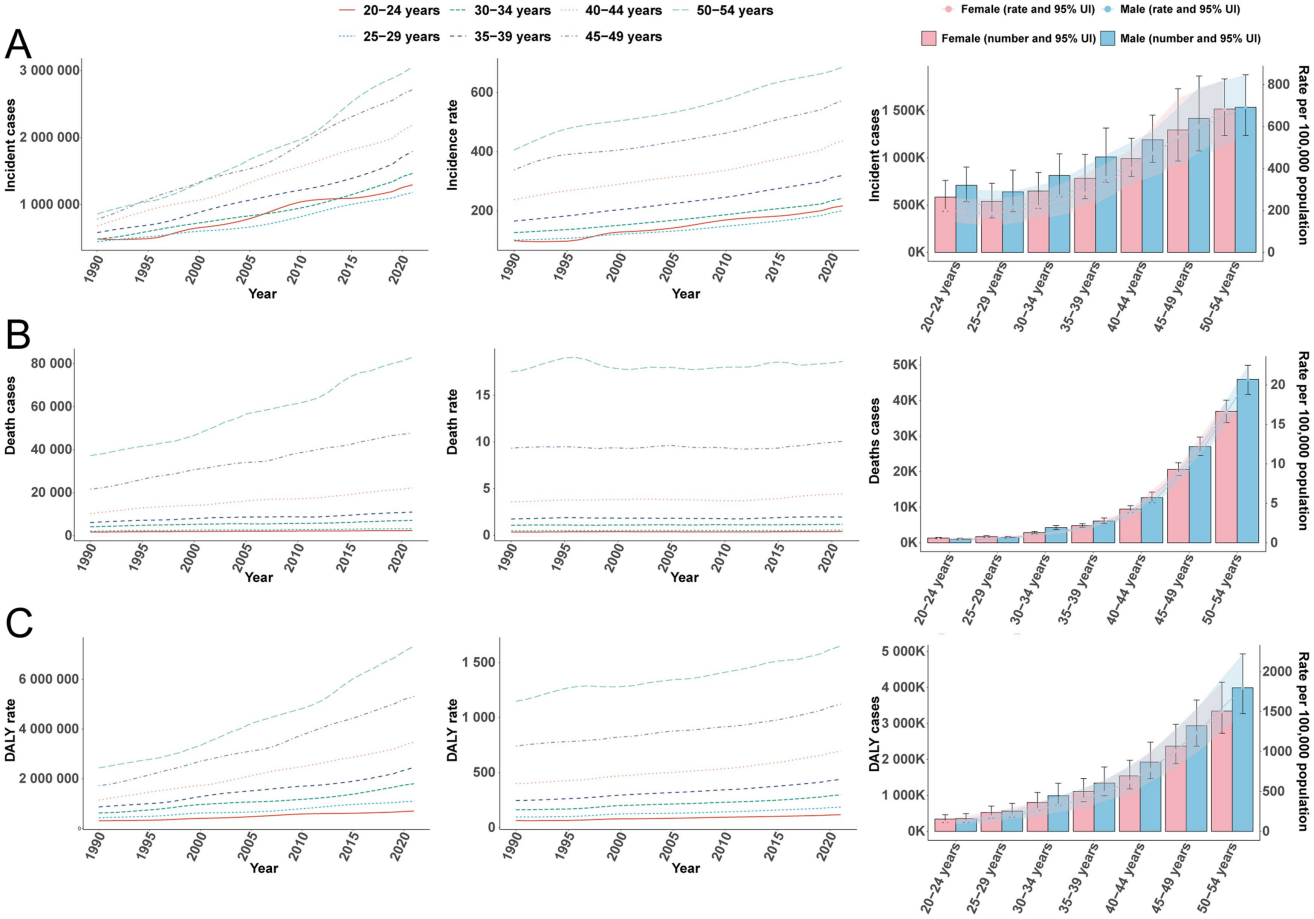
Trends in incidence, mortality, and disability-adjusted life years (DALYs) of type 2 diabetes in adults aged 20–54 years, by age and sex, 1990–2021. **(A)** Incidence (cases and rate). **(B)** Mortality (cases and rate). **(C)** DALYs (cases and rate).

Using GBD 2021, we quantified trends in incident T2D among adults aged 20–54 years during 1990–2021. The overall Average Annual Percent Change (AAPC) was 2.33% (95% CI, 2.29–2.37%; *p* < 0.001), with the steepest segment occurring in 2015–2021 (APC = 3.32, 95% CI, 3.10–3.54%; *p* < 0.001) (Figure 1A). Incident cases rose from 4,333,611 (95% UI, 3,791,811–4,995,279) in 1990 to 13,689,274 (95% UI, 12,154,334–15,474,678) in 2021—an absolute increase of 216% (95% UI, 205–227%). Over the same period, the global incidence rate increased from 180.29 per 100,000 (95% UI, 157.75–207.82) to 363.16 per 100,000 (95% UI, 322.44–410.52), a relative rise of 101% (95% UI, 94–109%). The EAPC in incidence was 2.21% (95% CI, 2.19–2.24%) (Table 1). All age groups experienced increases, most pronounced at ages 20–24 years (119%) and least at 50–54 years (69%) (Figure 2A). Across ages, men consistently had a higher incidence than women, with the largest sex gap at 40–44 years (Figure 2A).

**Table 1:** Incidence of type 2 diabetes in adults aged 20–54 years between 1990 and 2021 at the global and regional level.

	1990		2021		1990–2021		
Location	Incident cases	Incidence rate	Incident cases	Incidence rate	Cases change^b^	Rate change^b^	EAPC^a^
Global	4333611.20 (3791811.46, 4995279.48)	180.29 (157.75, 207.82)	13689273.79 (12154333.66, 15474678.27)	363.16 (322.44, 410.52)	215.89 (204.84, 227.17)	101.42 (94.38, 108.62)	Global
High SDI	872342.47 (774343.42, 994226.02)	197.47 (175.29, 225.07)	2368408.03 (2116316.99, 2655231.35)	458.72 (409.89, 514.27)	171.50 (162.25, 182.22)	132.29 (124.38, 141.46)	High SDI
High-middle SDI	933342.83 (813131.63, 1075694.50)	179.20 (156.12, 206.53)	2277153.09 (2003805.11, 2604545.31)	348.19 (306.40, 398.25)	143.98 (132.07, 156.49)	94.31 (84.82, 104.27)	High-middle SDI
Middle SDI	1517766.47 (1319520.00, 1765395.14)	193.40 (168.14, 224.95)	4511242.58 (3962670.40, 5154784.83)	366.92 (322.30, 419.26)	197.23 (182.91, 212.67)	89.72 (80.58, 99.58)	Middle SDI
Low-middle SDI	763198.75 (665403.51, 878602.54)	162.53 (141.70, 187.10)	3371179.69 (2965698.00, 3802578.03)	368.18 (323.89, 415.29)	341.72 (325.67, 357.94)	126.53 (118.31, 134.85)	Low-middle SDI
Low SDI	242021.72 (209106.74, 279044.83)	131.24 (113.39, 151.31)	1147284.59 (1005473.67, 1304645.60)	254.32 (222.88, 289.20)	374.04 (357.57, 391.32)	93.79 (87.05, 100.85)	Low SDI
Regions							
Andean Latin America	19532.83 (17558.61, 21823.37)	124.56 (111.97, 139.17)	92464.03 (82843.63, 102871.07)	284.04 (254.49, 316.01)	373.38 (343.74, 406.68)	128.03 (113.76, 144.08)	Andean Latin America
Australasia	12164.94 (10741.05, 14001.01)	120.88 (106.73, 139.13)	34871.66 (30223.49, 39391.22)	239.09 (207.22, 270.07)	186.66 (162.24, 213.82)	97.79 (80.94, 116.53)	Australasia
Caribbean	45343.59 (41107.71, 50241.77)	285.47 (258.80, 316.31)	140525.65 (126541.31, 156513.83)	612.64 (551.67, 682.34)	209.91 (191.67, 229.08)	114.61 (101.98, 127.88)	Caribbean
Central Asia	38413.54 (34595.04, 43598.05)	129.19 (116.35, 146.63)	163395.91 (148830.96, 180343.05)	350.38 (319.14, 386.72)	325.36 (295.23, 350.40)	171.21 (151.99, 187.17)	Central Asia
Central Europe	125328.06 (112073.38, 141149.91)	211.33 (188.98, 238.01)	218802.88 (193268.51, 247652.66)	400.16 (353.46, 452.92)	74.58 (66.46, 81.53)	89.35 (80.54, 96.88)	Central Europe
Central Latin America	224665.19 (200638.90, 253455.85)	329.39 (294.16, 371.60)	673124.74 (603081.05, 751533.71)	538.71 (482.66, 601.46)	199.61 (187.40, 214.19)	63.55 (56.88, 71.50)	Central Latin America
Central Sub-Saharan Africa	25561.46 (22093.64, 29570.49)	126.45 (109.30, 146.29)	142771.61 (126166.66, 163124.14)	262.72 (232.16, 300.17)	458.54 (420.40, 496.88)	107.76 (93.57, 122.02)	Central Sub-Saharan Africa
East Asia	1219518.01 (1033937.01, 1456070.94)	200.46 (169.96, 239.35)	2400054.80 (2057342.28, 2846108.11)	326.23 (279.64, 386.86)	96.80 (82.28, 113.77)	62.74 (50.73, 76.76)	East Asia
Eastern Europe	132169.40 (113734.76, 155017.70)	119.80 (103.09, 140.51)	268737.97 (230038.83, 314311.65)	272.77 (233.49, 319.03)	103.33 (92.63, 114.01)	127.69 (115.71, 139.65)	Eastern Europe
Eastern Sub-Saharan Africa	50207.20 (43474.28, 58113.04)	74.09 (64.16, 85.76)	207804.68 (179914.92, 238480.92)	121.19 (104.92, 139.08)	313.89 (299.31, 331.20)	63.56 (57.80, 70.40)	Eastern Sub-Saharan Africa
High-income Asia Pacific	216727.89 (189917.37, 249670.38)	246.04 (215.61, 283.44)	398756.41 (347353.13, 458043.80)	473.84 (412.76, 544.29)	83.99 (76.29, 92.65)	92.58 (84.52, 101.65)	High-income Asia Pacific
High-income North America	321092.13 (276171.13, 371487.01)	226.52 (194.83, 262.07)	951515.62 (853742.68, 1068164.34)	566.10 (507.93, 635.50)	196.34 (175.77, 218.89)	149.91 (132.57, 168.94)	High-income North America
North Africa and Middle East	251799.55 (224273.56, 285378.74)	187.69 (167.17, 212.72)	1879011.75 (1673961.01, 2116498.84)	605.60 (539.51, 682.14)	646.23 (609.87, 677.40)	222.65 (206.93, 236.13)	North Africa and Middle East
Oceania	9309.82 (8284.36, 10458.50)	344.49 (306.55, 387.00)	47648.38 (43032.01, 53076.31)	755.48 (682.29, 841.54)	411.81 (378.77, 443.76)	119.30 (105.15, 132.99)	Oceania
South Asia	802283.41 (685890.74, 937976.65)	175.92 (150.40, 205.68)	3416425.49 (2965686.39, 3921730.08)	373.47 (324.20, 428.71)	325.84 (310.23, 343.25)	112.29 (104.51, 120.97)	South Asia
Southeast Asia	272734.38 (238940.41, 313473.07)	134.18 (117.55, 154.22)	1022929.79 (902205.20, 1156903.62)	288.57 (254.52, 326.37)	275.06 (255.64, 295.65)	115.07 (103.93, 126.87)	Southeast Asia
Southern Latin America	38458.92 (34669.76, 43173.37)	172.95 (155.91, 194.15)	125796.17 (112474.47, 140878.77)	375.86 (336.06, 420.93)	227.09 (195.84, 254.40)	117.33 (96.56, 135.47)	Southern Latin America
Southern Sub-Saharan Africa	26823.88 (22817.59, 31421.55)	124.57 (105.97, 145.92)	96258.97 (82719.27, 112186.45)	244.91 (210.46, 285.43)	258.86 (238.58, 280.82)	96.60 (85.49, 108.63)	Southern Sub-Saharan Africa
Tropical Latin America	152200.77 (131261.24, 177906.37)	223.34 (192.61, 261.06)	419480.78 (358701.80, 493911.52)	359.64 (307.53, 423.45)	175.61 (160.35, 188.41)	61.03 (52.12, 68.51)	Tropical Latin America
Western Europe	274503.68 (241671.71, 312864.24)	145.27 (127.90, 165.58)	613031.83 (542656.29, 697417.91)	311.91 (276.10, 354.84)	123.32 (113.34, 134.27)	114.70 (105.11, 125.23)	Western Europe
Western Sub-Saharan Africa	74772.56 (64271.47, 87569.59)	104.97 (90.23, 122.94)	375864.68 (326079.48, 433632.27)	198.76 (172.43, 229.30)	402.68 (385.45, 419.91)	89.34 (82.85, 95.83)	Western Sub-Saharan Africa

EAPC, estimated annual percentage change; SDI, Sociodemographic Index; UI, uncertainty interval.

a EAPC is expressed as 95% confidence interval.

b Change shows the percentage change.

#### Mortality

In parallel with incidence, T2D mortality exhibited an overall rise with fluctuation over three decades. The AAPC was 0.99% (95% CI, 0.92–1.07%; *p* < 0.001), with the sharpest segment in 2017–2020 (APC = 2.19, 95% CI, 1.92–2.46%; *p* < 0.001) (Figure 1B). Deaths increased from 83,326 (95% UI, 78,973–87,754) in 1990 to 176,562 (95% UI, 163,156–189,590) in 2021 (112, 95% UI, 93–128%), while the mortality rate rose from 3.47 per 100,000 (95% UI, 3.29–3.65) to 4.68 per 100,000 (95% UI, 4.33–5.03) (+35%, 95% UI, 23–46%). The mortality EAPC was 0.90% (95% CI, 0.84–0.97%) (Table 2). Age-specific mortality was broadly stable but fluctuating over time, with a recent uptick among adults ≥40 years (Figure 2B). Death counts and rates rose with age, peaking at 50–54 years (Figure 2B), and differed by sex across age strata.

**Table 2:** Mortality of type 2 diabetes in adults aged 20–54 years between 1990 and 2021 at the global and regional level.

	1990		2021		1990–2021		
Location	Death cases	Death rate	Death cases	Death rate	Cases change^b^	Rate change^b^	EAPC^a^
Global	83326.35 (78973.37, 87754.46)	3.47 (3.29, 3.65)	176562.36 (163155.75, 189589.99)	4.68 (4.33, 5.03)	111.89 (92.61, 128.46)	35.11 (22.82, 45.68)	0.90 (0.84, 0.97)
High SDI	9977.47 (9739.22, 10253.59)	2.26 (2.20, 2.32)	12388.84 (11746.76, 13081.22)	2.40 (2.28, 2.53)	24.17 (16.79, 31.40)	6.24 (−0.08, 12.42)	−0.08 (−0.36, 0.20)
High-middle SDI	9800.34 (9168.08, 10520.41)	1.88 (1.76, 2.02)	14561.45 (13271.27, 15994.59)	2.23 (2.03, 2.45)	48.58 (31.99, 66.82)	18.33 (5.12, 32.86)	0.32 (0.13, 0.51)
Middle SDI	29108.89 (27514.56, 30536.35)	3.71 (3.51, 3.89)	67201.80 (62569.51, 71796.85)	5.47 (5.09, 5.84)	130.86 (109.26, 150.78)	47.36 (33.57, 60.07)	1.21 (1.11, 1.31)
Low-middle SDI	21911.95 (20186.31, 23785.21)	4.67 (4.30, 5.07)	55379.51 (49803.58, 61313.33)	6.05 (5.44, 6.70)	152.74 (120.55, 184.98)	29.62 (13.11, 46.15)	0.95 (0.89, 1.00)
Low SDI	12389.75 (10976.55, 13959.86)	6.72 (5.95, 7.57)	26795.39 (23244.50, 31117.32)	5.94 (5.15, 6.90)	116.27 (84.68, 151.71)	−11.59 (−24.50, 2.90)	−0.57 (−0.68,-0.47)
Regions							
Andean Latin America	582.23 (521.12, 657.84)	3.71 (3.32, 4.20)	1533.93 (1226.09, 1901.82)	4.71 (3.77, 5.84)	163.46 (103.10, 232.95)	26.91 (−2.16, 60.39)	0.59 (0.40, 0.78)
Australasia	136.74 (128.61, 145.00)	1.36 (1.28, 1.44)	205.99 (191.90, 221.73)	1.41 (1.32, 1.52)	50.65 (37.78, 64.96)	3.95 (−4.93, 13.82)	−0.40 (−0.79,-0.02)
Caribbean	1350.21 (1240.40, 1489.56)	8.50 (7.81, 9.38)	2189.33 (1767.15, 2661.41)	9.54 (7.70, 11.60)	62.15 (30.86, 97.28)	12.28 (−9.38, 36.61)	0.38 (0.27, 0.49)
Central Asia	640.33 (597.99, 676.17)	2.15 (2.01, 2.27)	1731.27 (1488.13, 2000.54)	3.71 (3.19, 4.29)	170.37 (130.48, 212.84)	72.39 (46.95, 99.47)	1.51 (1.15, 1.87)
Central Europe	1399.69 (1350.66, 1448.58)	2.36 (2.28, 2.44)	1260.67 (1155.23, 1367.29)	2.31 (2.11, 2.50)	−9.93 (−17.65,-2.47)	−2.31 (−10.69, 5.78)	0.03 (−0.17, 0.23)
Central Latin America	6123.02 (5991.69, 6269.92)	8.98 (8.78, 9.19)	16251.57 (14493.67, 18169.23)	13.01 (11.60, 14.54)	165.42 (137.29, 197.89)	44.88 (29.53, 62.61)	0.92 (0.53, 1.32)
Central Sub-Saharan Africa	1854.46 (1452.25, 2304.51)	9.17 (7.18, 11.40)	4917.47 (3742.64, 6413.55)	9.05 (6.89, 11.80)	165.17 (98.40, 254.42)	−1.37 (−26.20, 31.83)	0.03 (−0.08, 0.14)
East Asia	9812.12 (8492.26, 11245.90)	1.61 (1.40, 1.85)	14320.59 (11711.60, 17317.62)	1.95 (1.59, 2.35)	45.95 (14.96, 89.61)	20.68 (−4.94, 56.78)	0.45 (0.12, 0.78)
Eastern Europe	1136.04 (1044.01, 1198.45)	1.03 (0.95, 1.09)	1963.43 (1810.86, 2121.48)	1.99 (1.84, 2.15)	72.83 (57.34, 93.14)	93.54 (76.19, 116.27)	0.71 (−0.07, 1.49)
Eastern Sub-Saharan Africa	5944.66 (5127.10, 6962.66)	8.77 (7.57, 10.28)	11429.61 (9707.20, 13628.81)	6.67 (5.66, 7.95)	92.27 (57.19, 130.89)	−24.02 (−37.88,-8.76)	−1.18 (−1.31,-1.06)
High-income Asia Pacific	2093.27 (1970.39, 2242.51)	2.38 (2.24, 2.55)	947.52 (859.99, 1041.87)	1.13 (1.02, 1.24)	−54.73 (−59.48,-49.45)	−52.62 (−57.59,-47.08)	−2.55 (−2.79,-2.30)
High-income North America	4136.34 (4024.51, 4268.70)	2.92 (2.84, 3.01)	5963.29 (5774.24, 6154.47)	3.55 (3.44, 3.66)	44.17 (37.82, 50.20)	21.58 (16.23, 26.67)	0.23 (−0.22, 0.68)
North Africa and Middle East	4611.69 (4094.11, 5265.39)	3.44 (3.05, 3.92)	13661.70 (11404.86, 16152.11)	4.40 (3.68, 5.21)	196.24 (133.18, 247.29)	28.09 (0.82, 50.16)	0.93 (0.78, 1.07)
Oceania	675.61 (505.57, 855.48)	25.00 (18.71, 31.66)	1755.70 (1422.52, 2181.84)	27.84 (22.55, 34.59)	159.87 (93.01, 245.14)	11.35 (−17.30, 47.89)	0.37 (0.32, 0.43)
South Asia	17665.87 (16027.07, 19059.88)	3.87 (3.51, 4.18)	45561.31 (39869.28, 51028.61)	4.98 (4.36, 5.58)	157.91 (120.12, 200.35)	28.57 (9.73, 49.73)	0.91 (0.75, 1.07)
Southeast Asia	12095.56 (10724.86, 13847.69)	5.95 (5.28, 6.81)	28284.23 (24814.82, 32016.25)	7.98 (7.00, 9.03)	133.84 (99.48, 175.81)	34.09 (14.39, 58.15)	1.03 (0.96, 1.10)
Southern Latin America	692.17 (650.18, 739.24)	3.11 (2.92, 3.32)	802.80 (748.70, 858.93)	2.40 (2.24, 2.57)	15.98 (5.33, 27.83)	−22.94 (−30.02,-15.06)	−1.03 (−1.20,-0.86)
Southern Sub-Saharan Africa	1533.60 (1406.74, 1675.77)	7.12 (6.53, 7.78)	4217.55 (3789.80, 4650.98)	10.73 (9.64, 11.83)	175.01 (135.58, 212.74)	50.66 (29.06, 71.33)	1.89 (1.49, 2.29)
Tropical Latin America	4370.99 (4251.02, 4487.86)	6.41 (6.24, 6.59)	6650.52 (6416.17, 6903.40)	5.70 (5.50, 5.92)	52.15 (45.35, 58.98)	−11.10 (−15.08,-7.12)	−0.64 (−0.77,-0.51)
Western Europe	2718.90 (2642.00, 2803.30)	1.44 (1.40, 1.48)	1936.90 (1868.61, 2010.13)	0.99 (0.95, 1.02)	−28.76 (−31.77,-25.36)	−31.51 (−34.40,-28.24)	−1.01 (−1.20,-0.83)
Western Sub-Saharan Africa	3752.86 (3113.45, 4503.50)	5.27 (4.37, 6.32)	10976.98 (8850.20, 13130.48)	5.80 (4.68, 6.94)	192.50 (134.53, 262.69)	10.17 (−11.66, 36.61)	0.28 (0.19, 0.37)

EAPC, estimated annual percentage change; SDI, Sociodemographic Index; UI, uncertainty interval.

a EAPC is expressed as 95% confidence interval.

b Change shows the percentage change.

#### DALYs

Consistent with the incidence and mortality patterns, T2D DALYs increased globally. The fastest growth occurred from 2007 to 2015 (APC = 1.44%, 95% CI, 1.24–1.65%), with a peak segment APC of 2.95% (95% CI, 2.52–3.38%) (Figure 1C). DALYs rose from 7,532,385 (95% UI, 6,236,921–9,025,902) in 1990 to 22,174,878 (95% UI, 17,433,361–27,934,331) in 2021 (194%, 95% UI, 176–212%), and the DALY rate increased from 313.38 per 100,000 (95% UI, 259.48–375.51) to 588.27 per 100,000 (95% UI, 462.48–741.06) (88%, 95% UI, 76–99%). The EAPC in DALY rate was 2.00% (95% CI, 1.95–2.06%) (Table 3). DALY rates increased with age across the 20–54-year span and rose over time in all age groups; men consistently carried a higher DALY burden than women (Figure 2C).

**Table 3:** DALYs of type 2 diabetes in adults aged 20–54 years between 1990 and 2021 at the global and regional level.

	1990		2021		1990–2021		
Location	DALY cases	DALY rate	DALY cases	DALY rate	Cases change^b^	Rate change^b^	EAPC^a^
Global	7532385.09 (6236920.83, 9025901.78)	313.38 (259.48, 375.51)	22174878.36 (17433361.35, 27934331.25)	588.27 (462.48, 741.06)	194.39 (175.62, 211.73)	87.72 (75.75, 98.77)	2.00 (1.95, 2.06)
High SDI	1070834.60 (863078.23, 1319303.55)	242.41 (195.38, 298.65)	2673110.57 (1977890.15, 3531590.03)	517.73 (383.08, 684.00)	149.63 (127.94, 167.72)	113.58 (95.02, 129.06)	2.33 (2.22, 2.45)
High-middle SDI	1248465.01 (971311.37, 1584653.54)	239.70 (186.49, 304.25)	3230948.76 (2362025.76, 4264783.90)	494.04 (361.17, 652.12)	158.79 (140.87, 174.53)	106.10 (91.83, 118.64)	2.34 (2.18, 2.50)
Middle SDI	2697757.46 (2230792.05, 3247370.09)	343.75 (284.25, 413.79)	7913453.48 (6321014.42, 9828171.84)	643.63 (514.12, 799.37)	193.33 (177.53, 210.43)	87.24 (77.15, 98.15)	1.99 (1.92, 2.07)
Low-middle SDI	1705358.36 (1459364.91, 1998542.62)	363.16 (310.78, 425.60)	5867754.45 (4744114.43, 7234384.14)	640.84 (518.12, 790.09)	244.08 (213.53, 272.53)	76.46 (60.79, 91.05)	1.86 (1.79, 1.94)
Low SDI	799511.33 (707673.69, 920947.93)	433.54 (383.74, 499.39)	2465546.47 (2048872.33, 3023885.54)	546.54 (454.18, 670.31)	208.38 (175.21, 240.71)	26.07 (12.51, 39.28)	0.60 (0.50, 0.71)
Regions							
Andean Latin America	42831.39 (36240.50, 50916.47)	273.13 (231.10, 324.69)	148029.17 (119204.76, 185696.27)	454.73 (366.18, 570.44)	245.61 (197.34, 291.55)	66.49 (43.23, 88.62)	1.59 (1.51, 1.67)
Australasia	14223.77 (11307.34, 17890.42)	141.34 (112.36, 177.77)	34437.33 (25608.68, 45451.81)	236.11 (175.58, 311.62)	142.11 (116.48, 170.42)	67.05 (49.37, 86.58)	1.60 (1.48, 1.73)
Caribbean	104592.50 (89113.70, 125207.58)	658.48 (561.03, 788.27)	252787.05 (198480.72, 325985.46)	1102.06 (865.30, 1421.18)	141.69 (114.80, 166.32)	67.36 (48.74, 84.42)	1.70 (1.65, 1.75)
Central Asia	63926.95 (51058.05, 80250.07)	215.00 (171.72, 269.89)	233229.94 (178855.01, 305332.38)	500.12 (383.53, 654.74)	264.84 (237.57, 291.30)	132.62 (115.23, 149.49)	2.69 (2.47, 2.91)
Central Europe	158920.52 (124369.47, 200204.57)	267.98 (209.72, 337.59)	230697.76 (170518.79, 305635.36)	421.92 (311.86, 558.97)	45.17 (35.22, 54.20)	57.44 (46.65, 67.24)	1.40 (1.31, 1.48)
Central Latin America	501403.39 (425690.69, 588690.32)	735.12 (624.12, 863.10)	1468202.78 (1220393.20, 1792672.00)	1175.03 (976.70, 1434.70)	192.82 (175.94, 212.28)	59.84 (50.63, 70.46)	1.38 (1.22, 1.55)
Central Sub-Saharan Africa	110842.08 (90527.70, 134574.93)	548.34 (447.84, 665.74)	394163.55 (317790.03, 492722.90)	725.31 (584.77, 906.67)	255.61 (183.35, 325.00)	32.27 (5.40, 58.09)	0.94 (0.85, 1.03)
East Asia	1536244.09 (1151093.66, 2010153.40)	252.53 (189.22, 330.43)	4036151.52 (2854297.41, 5443219.14)	548.61 (387.97, 739.87)	162.73 (139.93, 184.77)	117.25 (98.40, 135.48)	2.59 (2.30, 2.87)
Eastern Europe	179000.06 (136072.88, 228263.47)	162.25 (123.34, 206.91)	345680.45 (260654.03, 449857.01)	350.87 (264.57, 456.61)	93.12 (84.36, 102.21)	116.25 (106.44, 126.44)	2.06 (1.87, 2.26)
Eastern Sub-Saharan Africa	323484.08 (283526.36, 373437.99)	477.38 (418.42, 551.10)	777633.90 (670231.66, 926108.80)	453.50 (390.86, 540.08)	140.39 (108.27, 179.27)	−5.00 (−17.70, 10.36)	−0.43 (−0.56,-0.30)
High-income Asia Pacific	267542.43 (209282.18, 336550.65)	303.73 (237.59, 382.07)	481984.54 (335891.45, 664778.70)	572.74 (399.14, 789.96)	80.15 (59.49, 99.86)	88.57 (66.94, 109.20)	2.02 (1.88, 2.15)
High-income North America	361578.71 (301240.94, 432734.49)	255.08 (212.51, 305.28)	958210.25 (729709.77, 1230739.57)	570.08 (434.14, 732.22)	165.01 (139.84, 189.13)	123.49 (102.27, 143.84)	2.47 (2.19, 2.74)
North Africa and Middle East	398361.03 (328980.66, 485139.55)	296.94 (245.22, 361.62)	2139877.78 (1631842.86, 2802775.16)	689.67 (525.94, 903.32)	437.17 (366.82, 492.79)	132.26 (101.84, 156.31)	2.82 (2.66, 2.97)
Oceania	39081.27 (30985.60, 48148.22)	1446.13 (1146.57, 1781.64)	128619.28 (105647.30, 158963.13)	2039.30 (1675.07, 2520.41)	229.11 (162.45, 305.50)	41.02 (12.46, 73.75)	1.11 (1.08, 1.14)
South Asia	1572069.94 (1305567.69, 1869134.41)	344.72 (286.28, 409.86)	5627008.61 (4389683.30, 7114409.48)	615.12 (479.86, 777.72)	257.94 (225.98, 286.98)	78.44 (62.51, 92.92)	1.88 (1.74, 2.01)
Southeast Asia	779198.75 (679835.09, 899716.88)	383.34 (334.46, 442.63)	2182014.87 (1845000.11, 2601124.43)	615.56 (520.48, 733.79)	180.03 (150.07, 212.07)	60.58 (43.39, 78.94)	1.30 (1.22, 1.38)
Southern Latin America	53030.90 (44890.40, 63072.40)	238.47 (201.87, 283.63)	121310.62 (90905.25, 159930.11)	362.46 (271.61, 477.85)	128.75 (101.59, 157.01)	51.99 (33.95, 70.76)	1.29 (1.22, 1.35)
Southern Sub-Saharan Africa	97260.70 (87129.62, 109910.00)	451.69 (404.64, 510.43)	281776.89 (244755.33, 324594.79)	716.92 (622.72, 825.86)	189.71 (160.23, 215.54)	58.72 (42.56, 72.87)	1.81 (1.52, 2.10)
Tropical Latin America	330809.09 (290234.32, 380258.20)	485.42 (425.88, 557.98)	679115.15 (551272.57, 838648.75)	582.23 (472.63, 719.01)	105.29 (89.79, 120.61)	19.94 (10.89, 28.90)	0.49 (0.39, 0.58)
Western Europe	348905.14 (272837.56, 442217.75)	184.65 (144.39, 234.03)	713812.39 (498937.40, 970427.19)	363.18 (253.86, 493.75)	104.59 (83.68, 120.33)	96.69 (76.59, 111.82)	2.24 (2.17, 2.30)
Western Sub-Saharan Africa	249078.28 (210228.98, 293464.04)	349.69 (295.15, 412.00)	940134.51 (777677.26, 1167138.97)	497.14 (411.23, 617.18)	277.45 (224.44, 332.07)	42.17 (22.20, 62.74)	1.13 (1.07, 1.18)

EAPC, estimated annual percentage change; SDI, Sociodemographic Index; UI, uncertainty interval; DALYs, Disability-adjusted life years.

a EAPC is expressed as 95% confidence interval.

b Change shows the percentage change.

### SDI regional trends

#### Incidence

**Figure 3:**
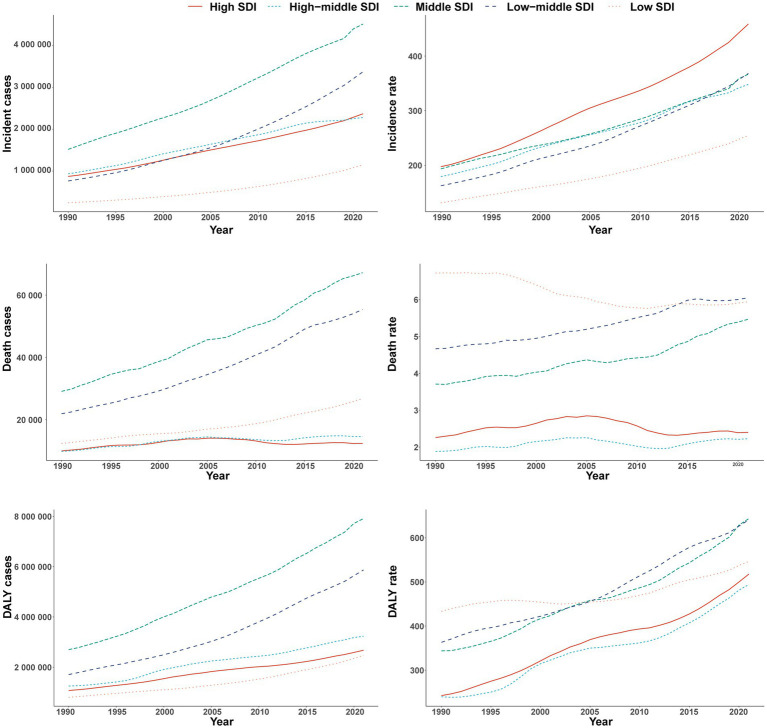
Incidence, mortality, and disability-adjusted life years of type 2 diabetes in adults aged 20–54 years across five Sociodemographic Index regions, 1990–2021.

In 2021, middle-SDI regions recorded the highest number of incident cases (4,511,243; 95% UI, 3,962,670–5,154,785), whereas high-SDI regions had the highest incidence rate (458.72 per 100,000; 95% UI, 409.89–514.27). The largest temporal increase in incidence was observed in high-SDI regions (EAPC = 2.68%, 95% CI, 2.62–2.74%) (Table 1 and Figure 3).

#### Mortality

High-SDI regions had 12,389 deaths (95% UI, 11,747–13,081) with a mortality rate of 2.40 per 100,000 (95% UI, 2.28–2.53). Deaths were numerically greatest in middle-SDI regions (67,202; 95% UI, 62,570–71,797), while lower-middle-SDI regions exhibited the highest mortality rate (6.05 per 100,000; 95% UI, 5.44–6.70). The steepest decline was in low-SDI regions (EAPC = −0.57%, 95% CI, −0.68% to −0.47%) (Table 2 and Figure 3).

#### DALYs

Middle-SDI regions bore the largest DALY count in 2021 (7,913,453; 95% UI, 6,321,014–9,828,172), with a DALY rate of 643.63 per 100,000 (95% UI, 514.12–799.37). The fastest DALY-rate growth was in high-middle SDI (EAPC = 2.34%, 95% CI, 2.18–2.50%), approaching that of high SDI (Table 3 and Figure 3).

### Geographic regional trends

#### Incidence

**Figure 4:**
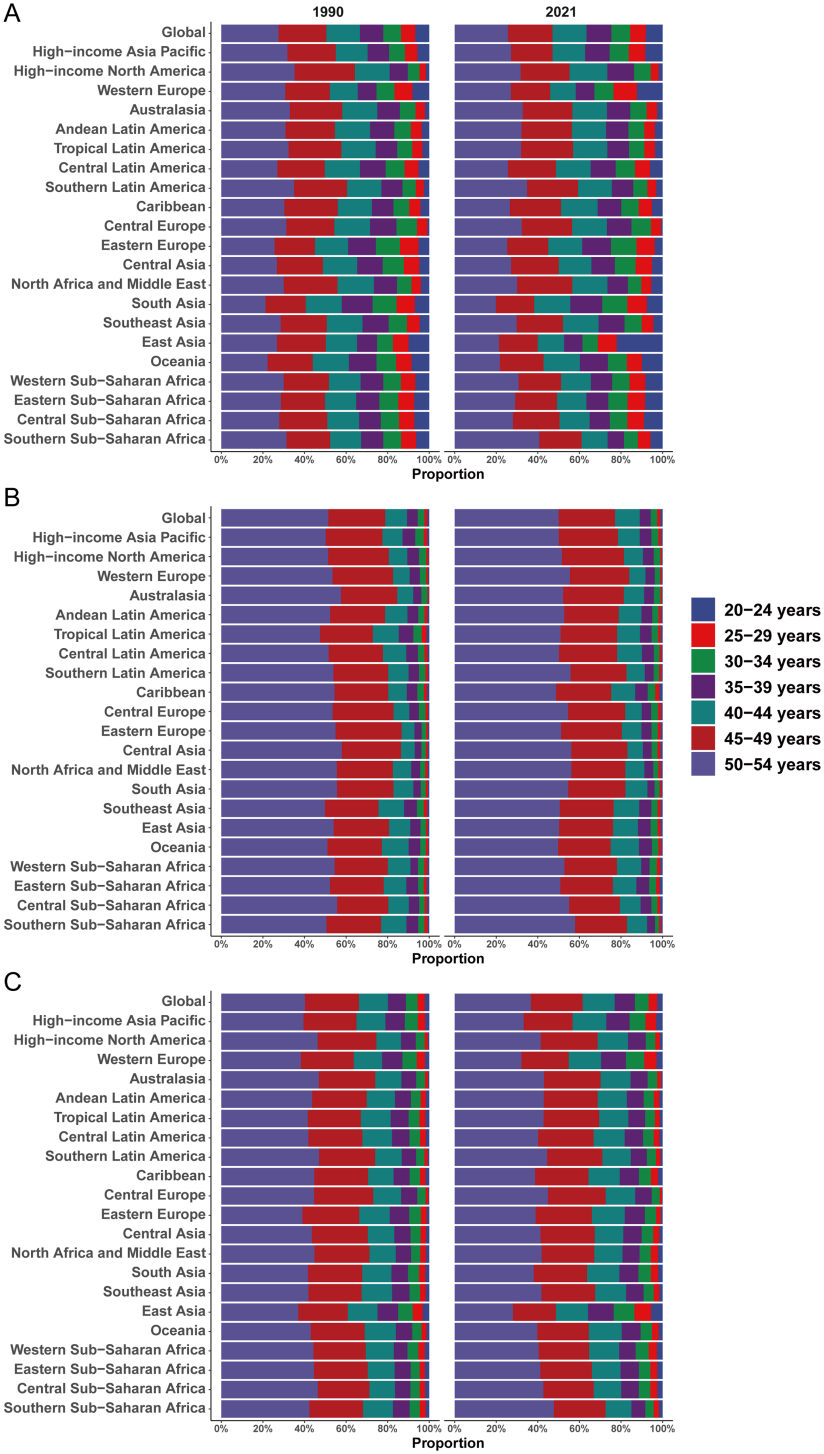
Age-specific percentages of type 2 diabetes (T2D)-related incidence, mortality, and disability-adjusted life years (DALYs) among adults aged 20–54 years in 1990 and 2021. **(A)** Incidence. **(B)** Mortality. **(C)** DALYs.

**Figure 5:**
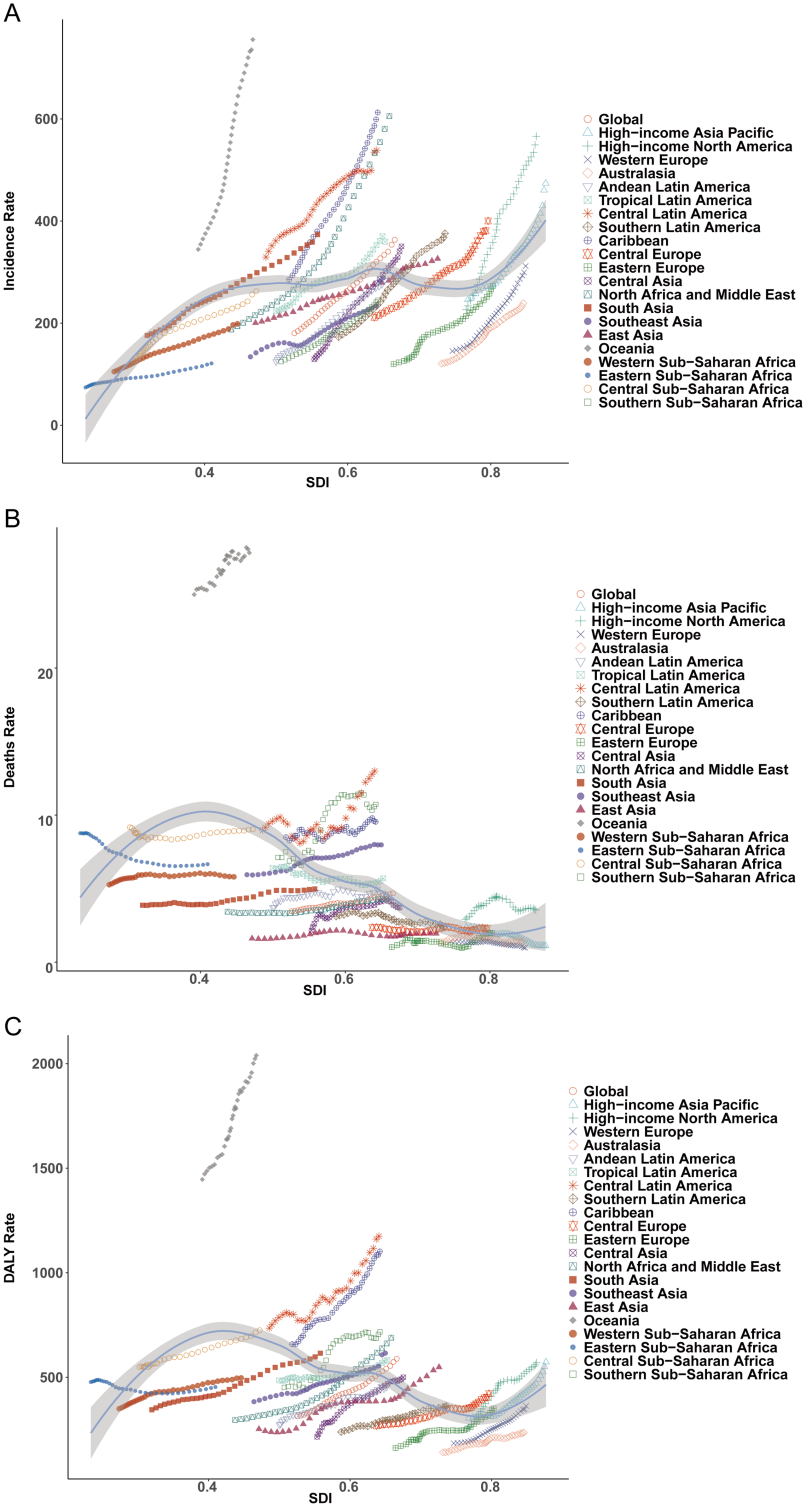
Association between the incidence, mortality, and disability-adjusted life years (DALYs) of type 2 diabetes among adults aged 20–54 years and the Sociodemographic Index (SDI) across regions, 1990–2021. **(A)** Incidence rate. **(B)** Mortality rate. **(C)** DALY rate.

In 2021, South Asia had the largest number of incident cases (3,416,425; 95% UI, 2,965,686–3,921,730), followed by East Asia (2,400,055; 95% UI, 2,057,342–2,846,108) and high-income North America (951,516; 95% UI, 853,743–1,068,164). Oceania had the highest incidence rate (755.48 per 100,000; 95% UI, 682.29–841.54), whereas Eastern Sub-Saharan Africa had the lowest (121.19 per 100,000; 95% UI, 104.92–139.08). Caribbean (612.64 per 100,000; 95% UI, 551.67–682.34) and high-income North America (566.10 per 100,000; 95% UI, 507.93–635.50) also showed high rates (Table 1). By age, Oceania’s rate at 50–54 years reached 1,270.92 per 100,000 in 2021, compared with 467.22 per 100,000 in Eastern Europe (Figure 4A). Incidence correlated positively with SDI (Spearman *r* = 0.34, 95% CI, 0.27–0.41; *p* < 0.001) (Figure 5A).

#### Mortality

South Asia recorded the most deaths (45,561; 95% UI, 39,869–51,029), followed by Southeast Asia (28,284; 95% UI, 24,815–32,016) and Central Latin America (16,252; 95% UI, 14,494–18,169). Australasia had the fewest deaths (206; 95% UI, 192–222). Mortality rates were highest in Oceania (27.84 per 100,000; 95% UI, 22.55–34.59), more than double Central America (13.01 per 100,000; 95% UI, 11.60–14.54) and Southern Sub-Saharan Africa (10.73 per 100,000; 95% UI, 9.64–11.83). Western Europe had the lowest mortality rate (0.99 per 100,000; 95% UI, 0.95–1.02) (Table 2). Age-specific mortality peaked at 50–54 years and was lowest at 20–24 years (Figure 4B). Mortality correlated inversely with SDI (Spearman *r* = −0.67, 95% CI, −0.70 to −0.62; *p* < 0.001) (Figure 5B).

### DALYs

DALYs were highest in South Asia (5,627,009; 95% UI, 4,389,683–7,114,409) and East Asia (4,036,152; 95% UI, 2,854,297–5,443,219) in 2021. North Africa and Middle East (2,139,878; 95% UI, 1,631,843–2,802,775) and Southeast Asia (2,182,015; 95% UI, 1,845,000–2,601,124) also carried substantial burdens; Central Latin America (1,468,203; 95% UI, 1,220,393–1,792,672) and Tropical Latin America (679,115; 95% UI, 551,273–838,649) remained prominent as well. High-income North America (958,210; 95% UI, 729,710–1,230,740) and Western Europe (713,812; 95% UI, 498,937–970,427) were lower (Table 3). Within Sub-Saharan Africa, West Africa had the highest DALYs (940,135; 95% UI, 777,677–1,167,139). Oceania had the highest DALY rate (2,039.30 per 100,000; 95% UI, 1,675.07–2,520.41), followed by the Caribbean (1,102.06; 95% UI, 865.30–1,421.18) and Central Latin America (1,175.03; 95% UI, 976.70–1,434.70). Rates were lowest in Australasia (236.11; 95% UI, 175.58–311.62) and Western Europe (363.18; 95% UI, 253.86–493.75). DALY rates increased steeply with age, e.g., from 63.4 (1990) to 117.1 (2021) at ages 20–24 years versus 1,148.1 (1990) to 1,650.2 (2021) at ages 50–54 years. Latin America, Sub-Saharan Africa, and Oceania exhibited high burdens, whereas high-income regions (Western Europe, Australasia) were comparatively low; notably, Central Latin America’s DALY rate at ages 50–54 years reached 3,795.3 in 2021 versus 736.7 in Australasia. Oceania’s burden was extreme—its 50–54-year DALY rate (7,309.5) was 4.4-fold the global mean (Figure 4C). DALY rates correlated negatively with SDI (Spearman *r* = −0.39, 95% CI, −0.44 to −0.32; *p* < 0.001) (Figure 5C).

### National trends

#### Incidence

**Figure 6:**
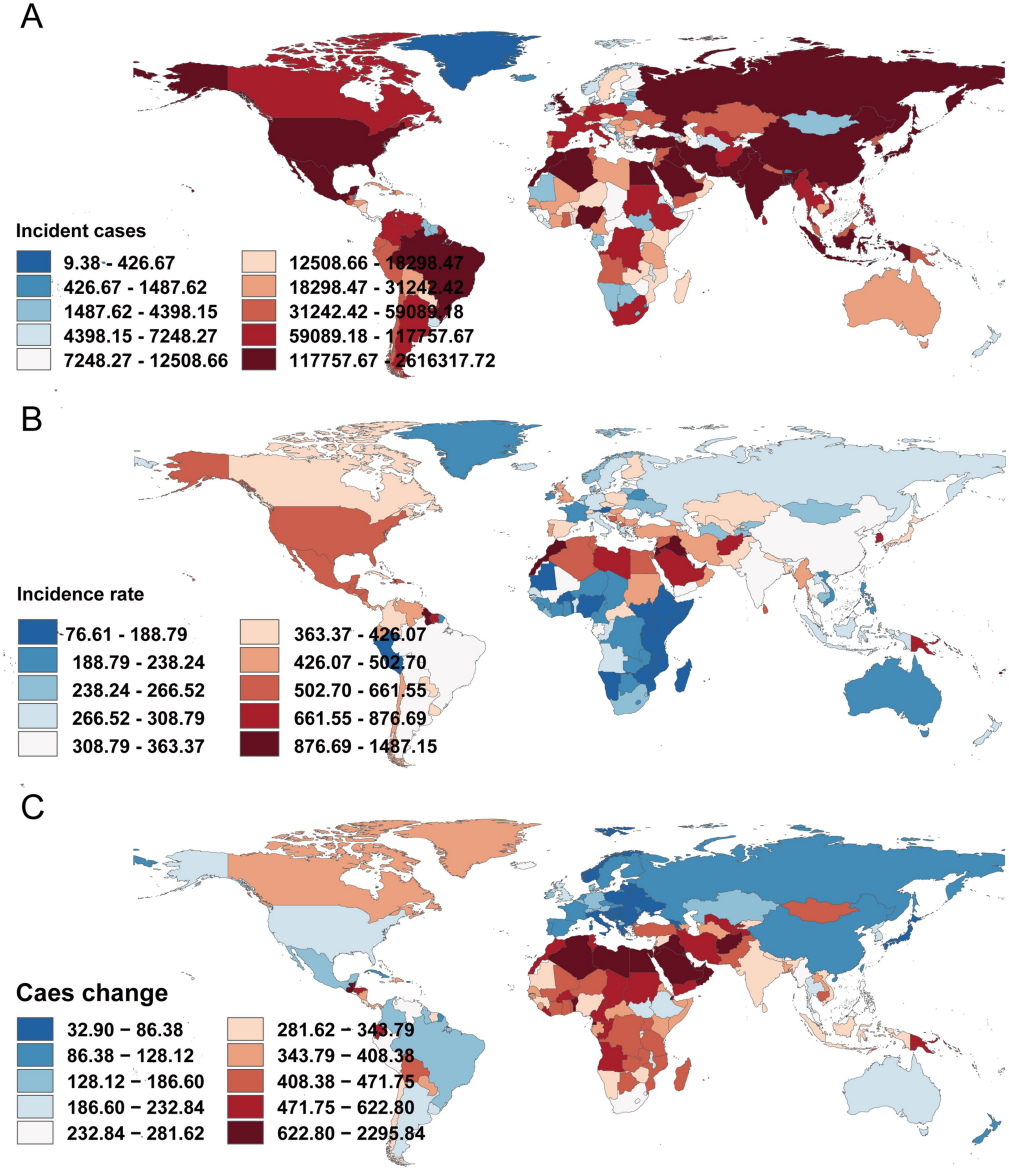
Incidence of type 2 diabetes in adults aged 20–54 years across 204 countries and territories. **(A)** Number of incident cases. **(B)** Incidence rate. **(C)** Change in incident cases.

In 2021, the largest numbers of incident cases were observed in India (2,590,414; 95% UI, 2,241,911–2,983,195), China (2,304,568; 95% UI, 1,969,275–2,740,463), and the United States (883,786; 95% UI, 789,746–995,357). The highest incidence rates were in Pacific Island settings: American Samoa (1,472.42 per 100,000; 95% UI, 1,299.18–1,658.36), the Marshall Islands (1,413.03; 95% UI, 1,274.86–1,572.13), and the Cook Islands (1,336.02; 95% UI, 1,186.40–1,477.49). Countries with the fastest increases (EAPC) included Greenland (5.65%, 95% CI, 5.55–5.75%), Egypt (5.01%, 95% CI, 4.88–5.14%), and Libya (4.51%, 95% CI, 4.43–4.59%) (Table S1 and Figure 6).

#### Mortality

Death counts were highest in India (33,854; 95% UI, 28,886–38,451), China (13,200; 95% UI, 10,507–16,137), and the United States (5,691; 95% UI, 5,508–5,882). Mortality rates peaked in the Federated States of Micronesia (37.21 per 100,000; 95% UI, 27.03–52.46), the Marshall Islands (53.24 per 100,000; 95% UI, 33.14–79.76), and Kiribati (52.05 per 100,000; 95% UI, 36.59–74.18). Many high-income countries had low mortality rates, including Iceland (0.33 per 100,000; 95% UI, 0.29–0.37), Sweden (0.86 per 100,000; 95% UI, 0.74–0.98), and Norway (0.74 per 100,000; 95% UI, 0.70–0.78) (Table S2).

#### DALYs

India (4,186,480; 95% UI, 3,263,244–5,280,942), China (3,883,238; 95% UI, 2,742,746–5,256,088), and the United States (905,523; 95% UI, 690,363–1,163,952) had the highest DALY counts in 2021. DALY rates were particularly elevated in Pacific Island countries and territories—American Samoa (3,627.65 per 100,000; 95% UI, 2,903.85–4,631.25), the Cook Islands (3,180.01; 95% UI, 2,503.55–4,080.80), and the Federated States of Micronesia (2,597.53; 95% UI, 1,984.41–3,316.85). By contrast, rates were low in many European countries (e.g., Denmark, 253.92 per 100,000; 95% UI, 178.48–341.86; Iceland, 302.92; 95% UI, 199.10–425.83). Several Middle Eastern countries showed high burdens, including Saudi Arabia (854.06 per 100,000; 95% UI, 638.50–1,113.24) and Iraq (1,146.94; 95% UI, 854.71–1,525.12) (Table S3).

### Risk factors

#### Mortality

**Figure 7:**
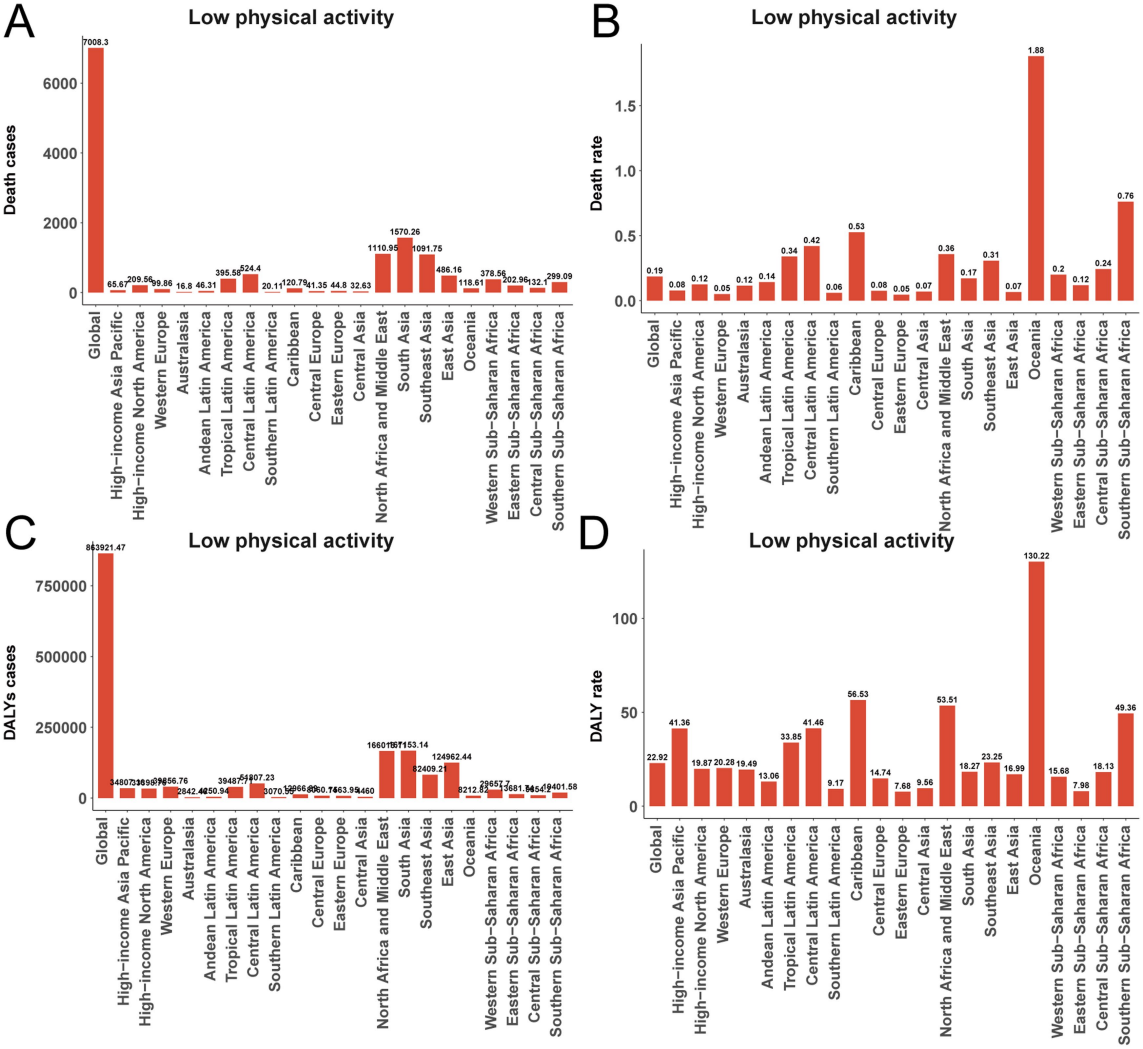
Low physical activity contributed to type 2 diabetes-related deaths and disability-adjusted life years (DALYs) among adults aged 20–54 years in 2021. **(A)** Number of deaths. **(B)** Death rate. **(C)** Number of DALYs. **(D)** DALY rate.

In 2021, 7,008 deaths (95% UI, 2,854–10,692) from T2D among adults aged 20–54 years were attributable to insufficient physical activity globally, corresponding to a mortality rate of 0.186 per 100,000 (95% UI, 0.08–0.28). Deaths were highest in South Asia (1,570.3; 95% UI, 624.4–2,604.0), followed by Southeast Asia (1,091.8) and North Africa and the Middle East (1,110.9). High-income Asia Pacific (65.7) and Australasia (16.8) had the lowest death counts. However, Oceania had the highest attributable mortality rate (1.881 per 100,000; 95% UI, 0.754–3.261), whereas Eastern Europe had the lowest (0.05; 95% UI, 0.02–0.08) (Figures 7A,B).

### DALYs

Globally, 863,921 DALYs (95% UI, 377,518–1,366,461) were attributable to low physical activity in this age group. East Asia (124,962 DALYs), South Asia (167,153), and North Africa and the Middle East (166,016) together accounted for 52.7% of the global total. Attributable DALY rates were highest in Oceania (130.22 per 100,000; 95% UI, 52.51–217.68), followed by the Caribbean (56.53; 95% UI, 23.55–94.00) and North Africa and the Middle East (53.51; 95% UI, 23.60–85.94). Rates were lowest in Eastern Europe (7.68; 95% UI, 2.94–13.55) and East Asia (16.99; 95% UI, 6.34–30.69). The global mean attributable DALY rate was 22.92 per 100,000 (95% UI, 10.01–36.25) (Figures 7C,D).

### Projection to 2035 by ARIMA

#### Incidence

**Figure 8:**
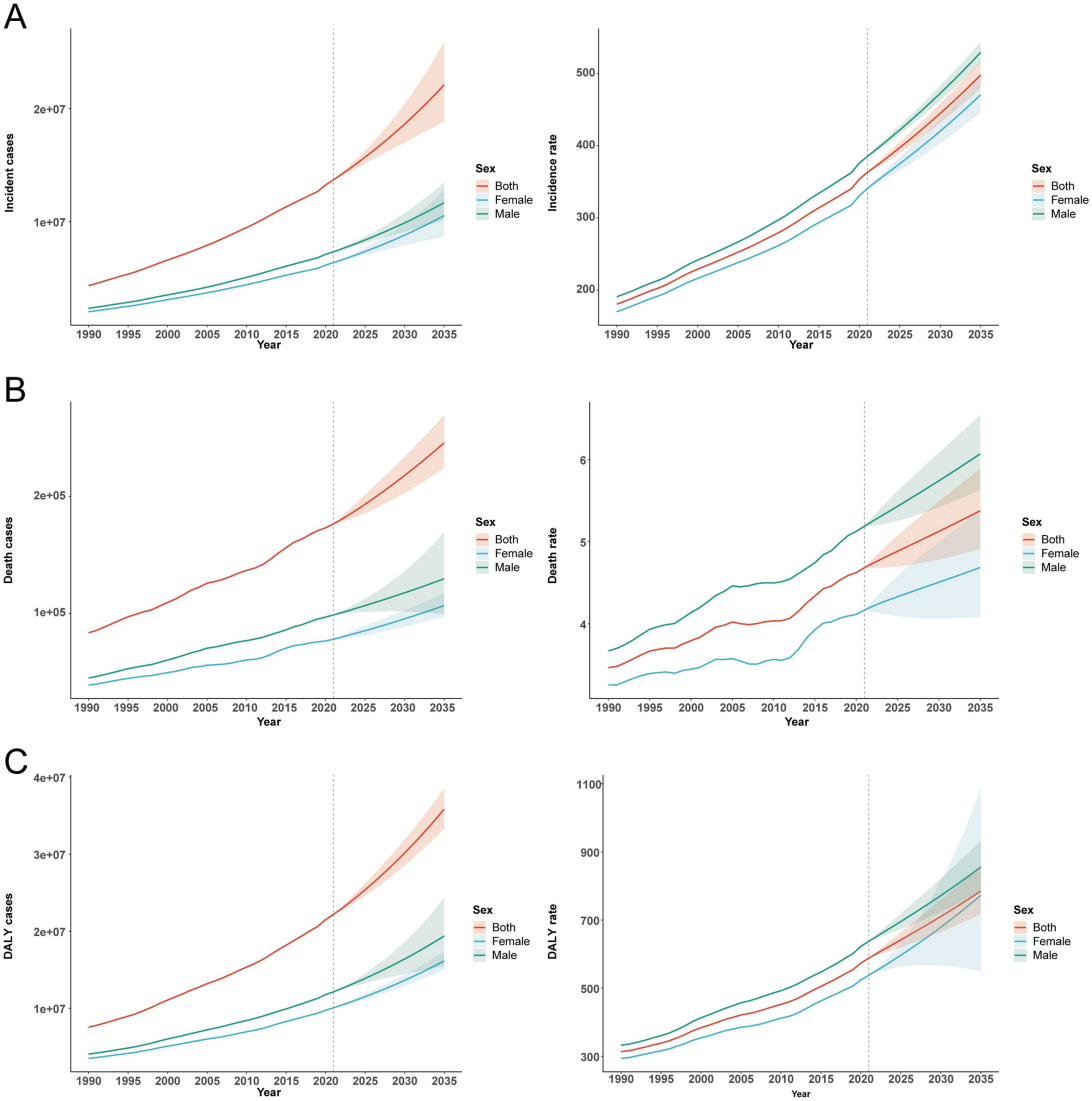
Forecast of the global type 2 diabetes burden among adults aged 20–54 years through 2035 utilizing an Autoregressive Integrated Moving Average (ARIMA) model. **(A)** Incidence (cases and rate). **(B)** Mortality (cases and rate). **(C)** Disability-adjusted life years (cases and rate).

From 1990 to 2021, incident cases increased steadily in men (from 2.31 million to 7.32 million) and women (from 2.01 million to 6.36 million). Projections for 2022–2035 indicate further acceleration, reaching 11.67 million in men, 10.53 million in women, and 22.10 million combined. Incidence rates rose historically from 190.56 to 385.21 per 100,000 in men, 169.77 to 340.71 in women, and 180.29 to 363.16 overall (average annual growth ≈2.5–3.0%), and are projected to continue increasing to 529.35 (men), 470.53 (women), and 497.84 (overall) by 2035 (Figure 8A).

#### Mortality

Deaths increased from 44,668 (men) and 38,658 (women) in 1990 to 98,681 and 77,881, respectively, in 2021 (combined, 83,326 to 176,562; average annual growth ≈3.0%). Projections suggest continued growth through 2035 to 129,630 (men), 106,670 (women), and 245,926 combined. Mortality rates are projected to continue rising in both sexes (Figure 8B).

### DALYs

In line with incidence and mortality, both DALY counts and DALY rates increased from 1990 to 2021 in men and women and are projected to rise further through 2035 (Figure 8C).

## Discussion

Our analysis of GBD 2021 data for adults aged 20–54 reveals pronounced regional disparities in T2D incidence, mortality, and DALYs. High-burden regions such as Oceania, South Asia, and Latin America carry a disproportionately large share of working-age T2D cases and health losses. In particular, Oceania’s highest incidence and DALY rates likely reflect a rapid nutrition and activity transition, swift shifts toward imported, ultra-processed foods; constrained access to fresh produce; motorization; and desk-based work, superimposed on high baseline adiposity at younger ages. Mechanistically, visceral and ectopic fat accumulation, together with early-life under-nutrition followed by energy surplus, may magnify insulin resistance and earlier diabetes onset. Furthermore, logistics (geography, small dispersed populations) can limit primary-care continuity and screening, increasing undetected hyperglycemia and delaying management. Taken together, these interacting factors provide a coherent explanation for the extreme working-age DALY rates and top-ranked incidence in the Pacific Islands in our results. By contrast, lower-burden regions like Western Europe and Australasia show substantially lower age-standardized T2D rates and slower increases. These discrepancies echo previous global surveys indicating that T2D risk is highly context-specific (5). Importantly, this disparity is evident not only in incidence but also in outcomes: T2D-related mortality rates among working-age adults in low-resource regions far exceed those in high-income settings, reflecting inequalities in both prevention and care.

Notably, the 2017–2020 acceleration we observed in mortality trends coincides with the onset of the COVID-19 pandemic, during which people with diabetes faced higher infection severity and care disruptions (postponed visits, reduced monitoring, and medicine access challenges). Moreover, movement restrictions likely reduced physical activity and worsened weight and glycemic control in many settings. While attribution is cautious, the temporal alignment suggests both direct viral effects and indirect system-level impacts plausibly contributed to the short-term rise in working-age mortality.

The divergent patterns by region likely arise from a mix of socioeconomic and lifestyle factors. Regions like the Pacific Islands and South Asia have undergone rapid nutrition transitions, characterized by high-calorie diets and sedentary habits, leading to obesity-driven diabetes epidemics at younger ages (24, 25). Additionally, health system capacity influences outcomes: Western Europe and Australasia generally have stronger primary care and diabetes management programs, which may mitigate mortality and complications despite any increases in T2D incidence (26). In lower-SDI regions, limited access to healthcare and earlier onset of disease contribute to higher DALYs per case. Indeed, our data suggest that while T2D incidence and prevalence have risen fastest in higher-SDI countries, the severity of the disease burden (measured by mortality and DALY rates) is greatest in lower-SDI settings. This finding is consistent with prior reports of a “double burden” in less-developed regions, increasing diabetes prevalence coupled with worse outcomes due to constrained health resources (27). Collectively, these global disparities highlight that T2D in working-aged adults is not one epidemic but many, varying widely by geography. Strategies to curb T2D must therefore be tailored to regional needs and capacities.

Our results also reinforce the central role of low physical activity in driving the T2D epidemic among adults 20–54. Physical inactivity emerged as one of the leading modifiable risk factors associated with T2D incidence and outcomes in this age group. Globally, approximately 7% of T2D DALYs in 2021 were directly attributable to insufficient physical activity, mirroring earlier estimates (15). In absolute terms, over 800,000 DALYs and 70,000 deaths from T2D were linked to low physical activity in 2021 alone. These figures have more than doubled since 1990, reflecting the worldwide pandemic of sedentary lifestyles. Our findings align with extensive epidemiological evidence that inadequate physical activity significantly increases the risk of developing T2D (28–30). Biologically, a sedentary lifestyle exacerbates insulin resistance and weight gain, creating a pathway to earlier T2D onset (31). Conversely, engaging in regular exercise has well-documented benefits: prospective trials have shown that moderate to vigorous physical activity improves glycemic control and reduces both cardiovascular and overall mortality in people with or at risk for T2D (32–34). In the landmark Diabetes Prevention Program, lifestyle intervention (diet and exercise) reduced progression to T2D by ~58% in high-risk individuals, far outperforming pharmacotherapy (35). Similarly, the Finnish DPS trial and other studies confirmed that even modest increases in physical activity can substantially delay or prevent T2D onset (36, 37). Moreover, for patients with established T2D, exercise interventions lead to better glycemic control and weight reduction, which translates into fewer complications (38, 39). Taken together, these findings establish physical inactivity as a critical modifiable driver of the T2D burden in young adults; yet, it represents only one component of a broader risk ensemble.

Accordingly, while low physical activity is modifiable, it accounts for only ~7% of the working-age T2D burden. This warrants moving beyond a solely individual, activity-focused narrative toward a multifactorial framework in which dietary patterns, environmental exposures, and their socioeconomic determinants together constitute the primary drivers of the T2D epidemic.

First, diet. High intake of refined carbohydrates, saturated fats, and ultra-processed foods, combined with insufficient consumption of whole grains and dietary fiber, disrupts glucose homeostasis and promotes obesity. Importantly, these choices are constrained by affordability, access to fresh produce (“food deserts”), and pervasive marketing, making poor diet both an individual risk and a manifestation of social inequity.

Second, the environment. Exposure to pollutants constitutes an increasingly recognized pathway. This includes (i) ambient air pollution (particularly PM₂.₅), which impairs insulin sensitivity via systemic inflammation and oxidative stress, and (ii) endocrine-disrupting chemicals (e.g., persistent organic pollutants and some plasticizers) that interfere with hormonal signaling and plausibly contribute to insulin resistance. This pathway is especially salient in rapidly growing economies, where industrialization can outpace safety regulation, elevating population exposures.

In short, the global distribution of T2D cannot be attributed solely to differences in physical activity. Rather, it reflects the interaction between rapid economic transformation and socio-ecological systems. The pace of socioeconomic development, dietary Westernization, increased environmental pollution, and proliferating urbanized lifestyles collectively shape each region’s risk trajectory. Populations in the most rapidly transitioning regions face a “synergistic shock” of multiple risks, from early-life under nutrition to adult over nutrition and toxicant exposure, inherently explaining the extreme working-age burden observed.

Additionally, the findings of this study have important public health implications, calling for targeted interventions stratified by sociodemographic level and region. A one-size-fits-all approach is unlikely to curb the global T2D burden given the heterogeneous drivers identified. Instead, evidence-based strategies must be tailored to regional needs and resources:

1. High-SDI Regions (e.g., Western Europe, North America, and Australia): These regions have relatively lower T2D incidence and mortality in younger adults, possibly reflecting past successes in health promotion and care. However, the continued rise in obesity and early-onset T2D even in affluent countries demands sustained preventive action. Public health authorities should reinforce lifestyle interventions that have proven effective – for instance, national diabetes prevention programs focusing on weight control and exercise for high-risk individuals (40, 41). Policies to reduce sedentary behavior (workplace wellness programs, active transport infrastructure) and to improve diet quality (sugar-sweetened beverage taxes, front-of-pack nutrition labeling) remain highly relevant in these settings. Moreover, with many cases still undiagnosed or developing in midlife, screening guidelines may need to be updated. In fact, the American Diabetes Association now recommends routine diabetes screening beginning at age 35 even in average-risk adults, a shift that could be adopted more widely in high-SDI countries to catch early cases. Continued investment in managing T2D is also critical: strengthening primary care to achieve tight risk factor control will help maintain the low mortality rates observed (42). High-SDI countries should also share best practices and support global efforts, given their resources and successful models for prevention and care.
2. Middle- and High-Middle SDI Regions (e.g., Latin America, Eastern Europe, East Asia): These regions are experiencing a rapid epidemiological transition, with T2D incidence climbing alongside economic development. Our results show significant increases in DALYs in these areas, which often face a dual challenge of high exposure to risk factors and constrained healthcare capacity. For middle-income countries, multi-sectoral prevention strategies are paramount. Governments should implement robust policies to promote physical activity, for example, urban planning that encourages walking/cycling, school-based physical education, and mass media campaigns to reduce sedentary lifestyles (43). At the same time, improving dietary environments is crucial: Latin American countries like Mexico and Chile have pioneered sugary drink taxes and junk-food marketing restrictions, yielding reductions in soda consumption and obesity rates (44). Scaling up such measures can help curb the upstream drivers of T2D. On the healthcare side, middle-SDI regions should expand access to essential diabetes care. This includes training primary healthcare workers in early diagnosis and management, subsidizing medications like metformin, and developing region-specific management guidelines (45). Crucially, these countries must monitor inequalities within their populations: rural and low-income urban groups often carry a higher diabetes burden and need particular support.
3. Low and Low-Middle SDI Regions (e.g., parts of Sub-Saharan Africa, some South Asian and Pacific nations): In low-SDI settings, diabetes incidence among young adults may still be relatively low today, but our findings warn of rapid growth and disproportionately severe outcomes. Health systems in these regions must act now to avoid a looming diabetes crisis. Integration of diabetes prevention into primary health care and infectious disease programs is one practical approach – for example, community health workers who already manage maternal or HIV care can be trained to deliver lifestyle counseling for NCD prevention (46). Given resource constraints, population-level interventions offer high return on investment: enforcing policies to limit trans fats, reduce salt and sugar in processed foods, and promote active living can reach broad swathes of the population at low cost. Low-income countries should also capitalize on co-benefits of interventions; for instance, improving urban walkability and public transit not only increases physical activity but also reduces air pollution and road injuries. Importantly, strengthening surveillance is a foundational step – many of these countries lack reliable data on diabetes incidence or outcomes. Implementing WHO STEP-wise surveys and building diabetes registries would enable more tailored strategies and help evaluate progress. From a treatment standpoint, even if prevention slows the tide, the number of individuals with T2D will inevitably grow given global trends. Low-SDI regions, therefore, need a basic package of diabetes care as part of universal health coverage commitments. This includes ensuring affordable access to insulin and oral hypoglycemics, blood glucose monitoring, and patient education to prevent acute complications. Without such measures, the high case-fatality rates observed in young adults in these regions will persist or worsen. International support and financing mechanisms (e.g., through the WHO Global Diabetes Compact or bilateral aid) should prioritize building this capacity in the lowest-SDI countries.

In summary, our recommendations stress a multifaceted response calibrated to the development level. High-SDI countries must sustain and innovate current prevention efforts, middle-SDI countries should implement bold policy and health system interventions to bend the curve of rising diabetes, and low-SDI countries need foundational health system strengthening and preventive policies to avert an oncoming epidemic. Across all regions, reducing physical inactivity stands out as a unifying strategy – increasing exercise through urban design, school and workplace initiatives, and public education will yield benefits far beyond T2D, impacting cardiovascular health, mental well-being, and more. Likewise, obesity prevention through improved nutrition is critical globally, given the strong influence of adiposity on T2D which we found to account for over half of T2D DALYs. Achieving these goals will require political commitment and cross-sector collaboration, as many determinants of diet and activity lie outside the health sector. Encouraging signs are emerging, from the UN’s inclusion of NCD targets in the Sustainable Development Goals to WHO’s recent Global Diabetes Compact, but concrete action must accelerate. The present analysis provides a clarion call: without region-specific, evidence-backed interventions, the global burden of type 2 diabetes in young and middle-aged adults will continue its alarming rise, entrenching health inequalities and straining economies. The tools for prevention are at hand; the task now is to implement them with urgency and at scale.

## Conclusion

T2D continues to rise globally and at younger ages, with stark geographic inequalities: Pacific Island states, South Asia, and parts of Latin America bear the highest rates and losses, while Western Europe and Australasia remain comparatively low. SDI patterns diverge, incidence growth is fastest in high-SDI settings, yet lower-middle SDI faces the highest mortality, and middle-SDI the largest absolute counts. Insufficient physical activity is a pervasive, tractable driver, especially where burdens are greatest. Without decisive action, incidence, deaths, and DALYs will keep climbing through 2035. Priorities should be region-tailored: scale population-wide activity and healthy food policies; expand early detection and risk-stratified prevention in high−/middle-SDI settings; and strengthen primary care, essential medicines, and surveillance in low-SDI systems, with urgent, culturally grounded programs for Pacific Island nations and other high-risk communities. Despite modeled, age-bounded estimates, the consistency of signals justifies rapid, coordinated action to curb incidence, compress complications, and narrow inequities.

## Data Availability

Publicly available datasets were analyzed in this study. This data can be found at: https://vizhub.healthdata.org/gbd-results/.

## Footnotes

1
https://vizhub.healthdata.org/gbd-results/
