# Supplementary material for: Burden of type 2 diabetes in working-age adults (20–54 years): a GBD 2021 analysis projecting trends to 2035 and exploring the potential benefits of physical activity
Source: Front Public Health. 2026 Jan 5;13:1706523. doi: 10.3389/fpubh.2025.1706523 (PMC12812608; doi:10.3389/fpubh.2025.1706523)
Supplement: Supplementary file 1 [file Table_1.docx]

Table S1: Incidence of type 2 diabetes in adults aged 20-54 years between 1990 and 2021 at the national level.

| location | 1990 | |  | 2021 | |  | 1990-2021 | |
| --- | --- | --- | --- | --- | --- | --- | --- | --- |
|  | Incident cases | Incidence rate |  | Incident cases | Incidence rate |  | Cases change | EAPC |
| Afghanistan | 11876.53(10130.90,13899.97) | 376.40(321.07,440.52) |  | 105780.53(93048.54,118799.02) | 865.87(761.65,972.43) |  | 790.67(706.11,898.28) | 3.26(2.86,3.67) |
| Albania | 1878.20(1612.47,2163.80) | 124.20(106.63,143.08) |  | 3067.71(2635.63,3672.43) | 242.61(208.44,290.44) |  | 63.33(48.06,79.50) | 2.22(2.04,2.40) |
| Algeria | 18473.26(15919.85,21455.94) | 190.91(164.52,221.73) |  | 138879.38(122291.48,156475.82) | 640.71(564.19,721.89) |  | 651.79(570.24,762.53) | 3.92(3.81,4.03) |
| American Samoa | 107.81(94.65,123.83) | 515.35(452.47,591.92) |  | 326.64(288.21,367.89) | 1472.42(1299.18,1658.36) |  | 202.98(173.64,236.52) | 3.37(3.13,3.61) |
| Andorra | 35.91(30.29,41.63) | 115.71(97.60,134.15) |  | 129.62(110.15,153.45) | 289.62(246.14,342.87) |  | 260.92(220.20,312.21) | 3.03(2.98,3.08) |
| Angola | 5981.30(5164.75,6969.58) | 153.87(132.86,179.29) |  | 37354.05(32641.55,42802.59) | 308.79(269.83,353.83) |  | 524.51(465.03,584.25) | 2.41(2.31,2.50) |
| Antigua and Barbuda | 93.18(80.72,106.84) | 336.44(291.43,385.75) |  | 345.40(299.92,391.66) | 732.34(635.90,830.42) |  | 270.67(228.59,322.22) | 2.65(2.56,2.74) |
| Argentina | 25796.08(23159.42,29003.55) | 177.66(159.50,199.75) |  | 79164.17(69675.73,89250.01) | 352.74(310.46,397.68) |  | 206.88(173.26,240.18) | 2.20(2.14,2.26) |
| Armenia | 2666.35(2350.93,3050.57) | 166.02(146.38,189.94) |  | 4763.73(4105.81,5412.36) | 329.55(284.03,374.42) |  | 78.66(61.55,94.14) | 2.12(1.78,2.46) |
| Australia | 9055.27(7935.84,10652.13) | 107.73(94.41,126.73) |  | 28172.98(23892.02,32384.07) | 231.96(196.71,266.63) |  | 211.12(176.78,252.39) | 2.49(2.34,2.64) |
| Austria | 2686.27(2323.55,3107.99) | 68.02(58.84,78.70) |  | 7392.50(6506.18,8484.29) | 173.04(152.29,198.59) |  | 175.20(151.14,204.13) | 3.19(3.10,3.29) |
| Azerbaijan | 3789.57(3330.16,4328.82) | 114.00(100.18,130.22) |  | 17519.43(15427.63,19778.05) | 316.37(278.60,357.16) |  | 362.31(317.06,411.76) | 3.58(3.33,3.83) |
| Bahamas | 384.25(341.67,431.99) | 308.67(274.47,347.02) |  | 1343.04(1163.63,1521.73) | 669.04(579.67,758.05) |  | 249.53(214.79,290.23) | 2.66(2.54,2.78) |
| Bahrain | 830.30(710.15,955.04) | 298.34(255.17,343.16) |  | 9372.48(8204.85,10797.75) | 972.54(851.38,1120.44) |  | 1028.81(897.78,1184.43) | 3.63(3.50,3.77) |
| Bangladesh | 64375.01(56386.95,73805.72) | 156.30(136.90,179.20) |  | 327271.30(294100.05,372635.16) | 407.66(366.34,464.16) |  | 408.38(368.45,456.41) | 3.26(3.14,3.38) |
| Barbados | 384.90(326.94,439.71) | 316.47(268.81,361.52) |  | 1004.39(878.47,1146.15) | 709.69(620.72,809.85) |  | 160.94(137.80,197.17) | 2.51(2.45,2.57) |
| Belarus | 5009.74(4342.88,5729.99) | 100.37(87.01,114.80) |  | 8529.88(7464.20,9824.30) | 192.86(168.77,222.13) |  | 70.27(48.39,91.96) | 1.95(1.83,2.07) |
| Belgium | 6457.60(5515.68,7532.60) | 132.19(112.91,154.19) |  | 14753.51(12573.67,17126.24) | 286.80(244.42,332.92) |  | 128.47(102.81,162.72) | 2.54(2.51,2.56) |
| Belize | 156.05(137.24,176.93) | 226.31(199.04,256.60) |  | 1159.31(1022.30,1327.78) | 552.21(486.95,632.46) |  | 642.92(553.61,749.15) | 3.07(2.95,3.20) |
| Benin | 2045.88(1759.61,2377.00) | 123.17(105.94,143.11) |  | 14977.22(13003.24,17408.10) | 290.49(252.21,337.64) |  | 632.07(571.74,699.06) | 2.55(2.35,2.75) |
| Bermuda | 64.62(56.04,75.57) | 196.41(170.31,229.67) |  | 126.67(109.65,149.43) | 440.54(381.37,519.72) |  | 96.01(76.10,118.94) | 2.54(2.47,2.61) |
| Bhutan | 362.80(314.37,417.78) | 145.26(125.87,167.28) |  | 1234.16(1102.35,1389.77) | 306.75(273.99,345.43) |  | 240.18(209.84,273.72) | 2.32(2.21,2.43) |
| Bolivia (Plurinational State of) | 3911.95(3450.26,4545.84) | 155.99(137.58,181.26) |  | 20089.21(17428.47,23438.76) | 352.14(305.50,410.86) |  | 413.53(369.26,470.73) | 2.60(2.55,2.65) |
| Bosnia and Herzegovina | 4373.88(3778.73,5054.22) | 191.59(165.52,221.39) |  | 8067.90(6918.10,9444.52) | 523.03(448.49,612.28) |  | 84.46(62.60,112.90) | 3.48(3.29,3.68) |
| Botswana | 482.07(409.46,572.37) | 99.23(84.29,117.82) |  | 2736.40(2376.81,3156.67) | 221.78(192.64,255.84) |  | 467.64(411.67,528.78) | 2.56(2.52,2.60) |
| Brazil | 149125.27(128446.52,174535.48) | 224.12(193.04,262.31) |  | 405263.68(344431.61,479148.45) | 358.22(304.45,423.53) |  | 171.76(156.30,184.20) | 1.56(1.47,1.66) |
| Brunei Darussalam | 306.92(269.91,348.21) | 238.71(209.92,270.83) |  | 2299.06(2021.91,2615.47) | 877.09(771.36,997.80) |  | 649.08(569.37,742.02) | 4.27(4.18,4.36) |
| Bulgaria | 8637.84(7585.32,9862.35) | 213.10(187.13,243.31) |  | 14671.73(12728.05,16583.18) | 470.89(408.51,532.24) |  | 69.85(51.50,95.29) | 2.58(2.49,2.68) |
| Burkina Faso | 2744.39(2350.65,3218.55) | 87.19(74.68,102.25) |  | 15755.52(13824.76,18332.77) | 186.41(163.57,216.91) |  | 474.10(425.43,531.25) | 2.48(2.34,2.62) |
| Burundi | 1507.32(1295.96,1739.02) | 74.55(64.10,86.01) |  | 6215.71(5367.69,7130.88) | 120.81(104.32,138.59) |  | 312.37(276.58,350.55) | 1.36(1.26,1.46) |
| Cabo Verde | 135.74(114.79,159.63) | 113.24(95.77,133.17) |  | 866.88(744.01,1009.30) | 299.06(256.67,348.19) |  | 538.62(466.86,612.35) | 3.60(3.35,3.85) |
| Cambodia | 3713.13(3222.91,4238.71) | 96.73(83.96,110.43) |  | 19652.06(17331.57,22389.28) | 238.52(210.36,271.74) |  | 429.26(386.71,481.70) | 2.88(2.83,2.92) |
| Cameroon | 3925.39(3393.85,4579.00) | 104.42(90.28,121.80) |  | 27714.14(23983.69,32295.35) | 215.82(186.77,251.49) |  | 606.02(543.17,667.49) | 2.35(2.26,2.44) |
| Canada | 13882.53(11739.35,16291.33) | 98.25(83.09,115.30) |  | 67651.65(57829.44,78775.93) | 398.67(340.79,464.23) |  | 387.31(323.60,467.07) | 4.14(3.91,4.38) |
| Central African Republic | 1861.60(1601.19,2172.21) | 178.20(153.27,207.93) |  | 8500.26(7398.34,9773.99) | 380.76(331.40,437.82) |  | 356.61(318.44,396.56) | 2.58(2.51,2.64) |
| Chad | 2103.04(1792.03,2463.60) | 103.20(87.94,120.90) |  | 12508.66(10837.01,14446.31) | 213.05(184.58,246.05) |  | 494.79(444.51,552.73) | 2.31(2.17,2.44) |
| Chile | 10589.91(9350.62,11991.21) | 167.32(147.74,189.46) |  | 40682.53(35316.88,47039.95) | 431.58(374.66,499.02) |  | 284.16(240.91,342.85) | 3.24(3.01,3.46) |
| China | 1183922.26(1001152.40,1418255.22) | 201.38(170.29,241.24) |  | 2304567.66(1969275.15,2740462.57) | 324.82(277.56,386.26) |  | 94.66(79.73,111.82) | 1.56(1.51,1.62) |
| Colombia | 33053.23(29187.70,37697.02) | 227.02(200.47,258.91) |  | 91738.95(81028.88,103251.24) | 367.88(324.93,414.04) |  | 177.55(151.29,211.03) | 1.26(1.14,1.37) |
| Comoros | 150.87(129.97,176.23) | 91.00(78.39,106.29) |  | 692.10(597.05,809.96) | 197.97(170.78,231.68) |  | 358.74(318.80,410.36) | 2.66(2.61,2.71) |
| Congo | 1181.85(1023.17,1367.51) | 131.74(114.06,152.44) |  | 7353.96(6270.02,8541.03) | 297.70(253.82,345.76) |  | 522.24(461.49,576.39) | 2.64(2.56,2.72) |
| Cook Islands | 49.56(43.15,56.29) | 605.99(527.65,688.27) |  | 104.90(93.15,116.01) | 1336.02(1186.40,1477.49) |  | 111.66(92.00,134.03) | 2.43(2.27,2.59) |
| Costa Rica | 2859.90(2505.30,3197.52) | 213.56(187.08,238.77) |  | 11116.85(9713.41,12873.71) | 459.10(401.14,531.65) |  | 288.71(251.81,329.69) | 2.38(2.24,2.52) |
| Croatia | 4955.05(4338.07,5728.73) | 204.60(179.13,236.55) |  | 7248.27(6239.89,8563.92) | 379.18(326.43,448.00) |  | 46.28(31.39,62.20) | 2.03(1.90,2.17) |
| Cuba | 11591.48(10276.91,13022.40) | 210.74(186.84,236.76) |  | 23224.24(20029.00,26579.69) | 426.03(367.42,487.59) |  | 100.36(75.18,124.51) | 2.36(2.30,2.42) |
| Cyprus | 618.71(531.84,713.52) | 163.12(140.22,188.12) |  | 2095.72(1788.41,2423.06) | 289.79(247.30,335.05) |  | 238.72(207.39,275.06) | 1.78(1.65,1.90) |
| Czechia | 9979.02(8561.66,11658.61) | 204.38(175.35,238.77) |  | 20955.65(17828.85,24353.40) | 426.07(362.49,495.15) |  | 110.00(83.83,142.53) | 2.23(2.10,2.36) |
| C么te d'Ivoire | 5312.38(4486.56,6172.16) | 114.52(96.72,133.06) |  | 27835.91(23849.35,32230.83) | 238.24(204.12,275.85) |  | 423.98(381.34,474.64) | 2.39(2.35,2.42) |
| Democratic People's Republic of Korea | 15961.72(13880.86,18749.80) | 157.40(136.88,184.90) |  | 46705.28(39933.95,53906.11) | 329.97(282.13,380.85) |  | 192.61(166.32,226.56) | 2.57(2.49,2.65) |
| Democratic Republic of the Congo | 15749.15(13533.33,18497.47) | 113.66(97.67,133.49) |  | 84931.99(74063.58,98194.37) | 235.46(205.33,272.23) |  | 439.28(388.54,491.07) | 2.28(2.22,2.34) |
| Denmark | 2560.49(2240.71,2876.51) | 98.91(86.55,111.11) |  | 6483.59(5645.28,7495.10) | 246.30(214.46,284.73) |  | 153.22(129.58,180.18) | 2.91(2.85,2.96) |
| Djibouti | 101.88(87.30,119.37) | 60.45(51.80,70.84) |  | 819.53(698.84,957.26) | 130.79(111.53,152.77) |  | 704.44(632.83,789.75) | 2.50(2.45,2.54) |
| Dominica | 102.46(90.83,118.93) | 343.46(304.48,398.68) |  | 242.57(209.46,277.97) | 744.79(643.16,853.49) |  | 136.74(113.02,164.05) | 2.63(2.53,2.72) |
| Dominican Republic | 6470.67(5660.18,7332.50) | 213.04(186.35,241.41) |  | 31242.42(27541.21,35551.62) | 573.13(505.23,652.18) |  | 382.83(336.51,434.09) | 3.36(3.26,3.45) |
| Ecuador | 6654.39(5915.38,7612.29) | 159.93(142.17,182.95) |  | 38171.67(33908.47,42398.56) | 439.68(390.58,488.37) |  | 473.63(413.20,533.19) | 3.47(3.34,3.60) |
| Egypt | 27585.24(24255.56,31737.14) | 120.25(105.74,138.35) |  | 253366.93(221042.40,292742.90) | 527.72(460.40,609.74) |  | 818.49(739.59,911.80) | 5.01(4.88,5.14) |
| El Salvador | 3798.31(3325.75,4342.03) | 184.27(161.34,210.64) |  | 13212.42(11620.46,15103.99) | 433.82(381.55,495.93) |  | 247.85(218.83,285.30) | 2.63(2.51,2.75) |
| Equatorial Guinea | 214.37(186.35,248.13) | 142.06(123.49,164.43) |  | 1890.24(1613.47,2192.09) | 285.07(243.33,330.59) |  | 781.78(703.74,866.01) | 2.32(2.23,2.42) |
| Eritrea | 1178.27(1011.41,1385.98) | 93.33(80.12,109.79) |  | 5467.81(4710.40,6335.95) | 185.94(160.18,215.46) |  | 364.06(331.08,410.68) | 2.24(2.21,2.28) |
| Estonia | 995.02(868.97,1154.66) | 132.55(115.75,153.81) |  | 1867.85(1621.26,2145.80) | 314.95(273.37,361.82) |  | 87.72(68.98,110.54) | 2.77(2.67,2.87) |
| Eswatini | 349.96(298.18,412.08) | 123.18(104.95,145.04) |  | 1335.52(1165.72,1550.93) | 252.13(220.08,292.80) |  | 281.62(249.62,318.83) | 2.37(2.17,2.58) |
| Ethiopia | 19551.97(16838.12,22887.41) | 109.30(94.13,127.95) |  | 61977.45(52701.69,72557.50) | 138.05(117.39,161.62) |  | 216.99(202.86,232.69) | 0.54(0.44,0.65) |
| Fiji | 1599.23(1415.17,1814.03) | 465.83(412.22,528.40) |  | 4810.36(4271.47,5384.03) | 1100.51(977.22,1231.75) |  | 200.79(168.31,239.19) | 2.75(2.73,2.77) |
| Finland | 4744.75(4104.18,5519.11) | 185.79(160.70,216.11) |  | 9310.93(8096.74,10594.08) | 392.08(340.95,446.12) |  | 96.24(75.80,114.51) | 2.29(2.23,2.35) |
| France | 27549.69(23571.30,31329.14) | 99.41(85.06,113.05) |  | 59665.05(52005.05,70701.68) | 209.14(182.29,247.83) |  | 116.57(93.49,142.51) | 2.65(2.44,2.85) |
| Gabon | 573.19(499.59,667.01) | 151.86(132.36,176.71) |  | 2741.11(2381.54,3172.67) | 338.29(293.91,391.55) |  | 378.22(338.64,428.38) | 2.59(2.50,2.69) |
| Gambia | 354.25(299.14,408.70) | 97.21(82.09,112.15) |  | 2125.70(1819.46,2448.29) | 218.49(187.02,251.65) |  | 500.06(450.08,563.60) | 2.69(2.63,2.75) |
| Georgia | 3757.09(3265.10,4435.80) | 143.65(124.84,169.60) |  | 6995.94(6109.86,7926.23) | 429.69(375.27,486.83) |  | 86.21(62.19,108.72) | 3.81(3.61,4.00) |
| Germany | 42333.94(37320.01,48322.66) | 101.70(89.65,116.08) |  | 109329.67(95771.03,125764.65) | 288.39(252.62,331.74) |  | 158.26(129.82,188.38) | 3.52(3.36,3.68) |
| Ghana | 6334.56(5428.12,7431.48) | 111.31(95.39,130.59) |  | 35868.91(30769.69,41834.23) | 235.91(202.37,275.14) |  | 466.24(417.07,517.27) | 2.47(2.35,2.59) |
| Greece | 8132.49(6949.69,9554.02) | 165.80(141.68,194.78) |  | 14144.35(11878.19,16736.25) | 307.88(258.55,364.29) |  | 73.92(52.19,95.30) | 1.95(1.85,2.05) |
| Greenland | 14.26(11.81,16.87) | 44.34(36.73,52.45) |  | 63.41(54.66,72.06) | 233.52(201.29,265.35) |  | 344.79(291.60,411.70) | 5.65(5.55,5.75) |
| Grenada | 116.09(102.15,132.65) | 354.01(311.48,404.51) |  | 426.67(366.57,503.64) | 813.72(699.10,960.50) |  | 267.53(221.27,315.92) | 2.69(2.64,2.75) |
| Guam | 170.31(150.86,192.91) | 244.11(216.23,276.50) |  | 388.38(344.27,443.86) | 531.84(471.43,607.81) |  | 128.04(107.34,155.20) | 2.50(2.46,2.55) |
| Guatemala | 5913.83(5296.36,6604.58) | 204.04(182.73,227.87) |  | 43111.46(38681.08,47882.96) | 588.82(528.31,653.99) |  | 628.99(557.14,694.72) | 3.67(3.47,3.88) |
| Guinea | 2162.98(1857.97,2548.15) | 100.44(86.28,118.33) |  | 9598.99(8306.76,11074.49) | 190.01(164.43,219.22) |  | 343.79(301.21,387.63) | 1.99(1.80,2.18) |
| Guinea-Bissau | 481.24(407.64,561.05) | 135.49(114.77,157.96) |  | 2152.65(1847.58,2457.38) | 259.19(222.46,295.89) |  | 347.31(312.52,396.03) | 2.05(2.02,2.09) |
| Guyana | 1727.31(1503.75,1974.98) | 512.89(446.51,586.43) |  | 4060.05(3594.62,4558.11) | 1093.15(967.83,1227.25) |  | 135.05(114.14,159.70) | 2.64(2.44,2.84) |
| Haiti | 9743.54(8463.74,10959.43) | 390.52(339.22,439.25) |  | 41996.18(36533.74,47420.40) | 691.73(601.76,781.07) |  | 331.02(293.79,371.94) | 1.75(1.70,1.80) |
| Honduras | 3968.14(3441.72,4507.59) | 238.58(206.93,271.02) |  | 23833.83(21037.62,27442.45) | 506.24(446.85,582.89) |  | 500.63(446.32,556.65) | 2.47(2.40,2.53) |
| Hungary | 11274.54(9855.56,12872.40) | 229.30(200.44,261.79) |  | 19468.44(17040.96,22109.86) | 428.65(375.20,486.81) |  | 72.68(54.10,91.50) | 1.91(1.71,2.11) |
| Iceland | 129.50(109.59,149.83) | 106.43(90.07,123.15) |  | 437.42(381.25,504.19) | 266.89(232.62,307.63) |  | 237.79(208.23,271.17) | 2.99(2.95,3.04) |
| India | 646554.81(549307.60,761333.62) | 176.66(150.09,208.03) |  | 2590413.58(2241911.42,2983195.04) | 363.37(314.49,418.47) |  | 300.65(284.82,318.49) | 2.27(2.20,2.34) |
| Indonesia | 104991.52(90002.02,123177.41) | 129.04(110.62,151.39) |  | 413636.07(352971.61,489156.14) | 281.74(240.42,333.17) |  | 293.97(270.46,319.25) | 1.28(0.67,1.90) |
| Iran (Islamic Republic of) | 29781.82(25648.15,35078.96) | 141.95(122.25,167.20) |  | 210481.38(179675.64,246988.36) | 452.55(386.31,531.04) |  | 606.74(560.23,654.86) | 3.54(3.37,3.71) |
| Iraq | 26126.63(23162.28,29460.55) | 381.05(337.81,429.67) |  | 193113.08(169807.49,219347.13) | 980.16(861.87,1113.31) |  | 639.14(571.84,722.32) | 3.24(3.12,3.36) |
| Ireland | 2000.44(1716.47,2317.03) | 125.61(107.78,145.49) |  | 5474.37(4671.01,6402.46) | 237.25(202.44,277.47) |  | 173.66(140.94,206.88) | 2.08(1.97,2.19) |
| Israel | 3487.12(3051.66,4008.28) | 162.63(142.32,186.94) |  | 13499.53(11438.80,15324.20) | 319.74(270.94,362.96) |  | 287.13(246.29,335.76) | 2.16(1.90,2.42) |
| Italy | 50974.37(42055.05,63013.14) | 182.31(150.41,225.37) |  | 75451.38(60488.76,93857.14) | 283.49(227.27,352.65) |  | 48.02(38.48,57.47) | 1.26(1.12,1.41) |
| Jamaica | 2250.36(2008.18,2519.49) | 229.98(205.23,257.49) |  | 7059.24(6183.95,8051.60) | 484.96(424.83,553.13) |  | 213.69(184.37,249.11) | 2.31(2.19,2.43) |
| Japan | 149449.16(125956.04,179187.70) | 237.78(200.40,285.10) |  | 199120.97(166278.30,243256.90) | 366.95(306.43,448.29) |  | 33.24(27.26,39.75) | 0.86(0.71,1.02) |
| Jordan | 4894.59(4356.58,5529.37) | 343.70(305.92,388.28) |  | 53974.56(47663.62,61224.13) | 878.74(776.00,996.77) |  | 1002.74(901.83,1110.17) | 3.45(3.33,3.57) |
| Kazakhstan | 12877.18(11214.31,15508.50) | 168.14(146.43,202.50) |  | 36906.10(33070.52,41129.65) | 407.44(365.10,454.07) |  | 186.60(149.16,220.56) | 2.99(2.89,3.08) |
| Kenya | 3944.21(3332.34,4628.25) | 49.16(41.54,57.69) |  | 18298.47(15562.20,21265.27) | 84.24(71.65,97.90) |  | 363.93(339.51,387.08) | 1.67(1.63,1.71) |
| Kiribati | 153.57(136.36,171.58) | 482.98(428.86,539.63) |  | 533.42(478.66,593.61) | 967.40(868.10,1076.57) |  | 247.35(221.02,274.04) | 2.25(2.06,2.43) |
| Kuwait | 3428.41(2970.90,3933.78) | 364.28(315.66,417.97) |  | 31882.78(27412.47,36842.67) | 1032.34(887.60,1192.94) |  | 829.96(703.73,972.87) | 3.58(3.52,3.64) |
| Kyrgyzstan | 1948.86(1689.06,2198.29) | 106.05(91.92,119.63) |  | 8434.04(7353.80,9570.02) | 264.02(230.20,299.58) |  | 332.77(293.30,388.58) | 3.10(2.95,3.25) |
| Lao People's Democratic Republic | 2109.18(1856.61,2432.47) | 135.37(119.16,156.12) |  | 10676.82(9410.39,12020.36) | 295.52(260.47,332.71) |  | 406.21(359.96,461.66) | 2.37(2.29,2.45) |
| Latvia | 1638.87(1439.21,1886.60) | 127.99(112.40,147.34) |  | 2652.81(2311.50,3045.18) | 321.46(280.11,369.01) |  | 61.87(44.32,81.60) | 3.03(2.91,3.15) |
| Lebanon | 4000.11(3531.49,4644.22) | 314.05(277.26,364.62) |  | 19924.16(17750.13,22449.01) | 688.43(613.31,775.67) |  | 398.09(355.46,449.26) | 2.59(2.48,2.71) |
| Lesotho | 469.50(398.17,556.78) | 84.53(71.69,100.25) |  | 1772.63(1539.23,2035.00) | 207.32(180.03,238.01) |  | 277.56(242.07,315.48) | 2.92(2.81,3.02) |
| Liberia | 1019.81(874.43,1191.18) | 112.56(96.51,131.48) |  | 6012.58(5127.71,7040.24) | 257.02(219.20,300.95) |  | 489.58(435.44,558.96) | 2.52(2.46,2.58) |
| Libya | 3374.04(2912.33,3946.03) | 211.45(182.51,247.29) |  | 29453.95(25633.86,34471.60) | 746.29(649.50,873.42) |  | 772.96(684.03,905.47) | 4.51(4.43,4.59) |
| Lithuania | 1964.73(1710.48,2269.08) | 110.74(96.41,127.89) |  | 3228.46(2846.13,3739.02) | 263.61(232.39,305.29) |  | 64.32(50.41,82.44) | 2.74(2.62,2.86) |
| Luxembourg | 259.71(221.47,299.40) | 130.14(110.98,150.03) |  | 883.91(753.05,1038.25) | 266.52(227.07,313.06) |  | 240.35(207.09,275.37) | 2.38(2.36,2.39) |
| Madagascar | 2687.73(2322.17,3112.15) | 62.08(53.63,71.88) |  | 13872.11(12095.76,16189.47) | 117.89(102.79,137.58) |  | 416.13(373.26,456.02) | 2.07(2.05,2.08) |
| Malawi | 1906.28(1654.01,2216.86) | 52.90(45.90,61.52) |  | 5949.18(5206.39,6795.35) | 77.38(67.72,88.39) |  | 212.08(181.33,240.32) | 0.98(0.84,1.11) |
| Malaysia | 16030.22(13929.77,18666.82) | 204.26(177.49,237.85) |  | 58897.72(51882.75,67139.32) | 353.65(311.53,403.14) |  | 267.42(227.31,308.85) | 1.68(1.46,1.89) |
| Maldives | 102.62(90.08,119.31) | 130.59(114.63,151.82) |  | 798.74(689.29,935.31) | 240.22(207.30,281.29) |  | 678.33(585.40,801.54) | 1.88(1.69,2.07) |
| Mali | 5631.36(4815.70,6611.58) | 184.74(157.98,216.89) |  | 29940.33(25684.78,35566.79) | 355.43(304.91,422.22) |  | 431.67(383.42,483.95) | 2.20(2.08,2.31) |
| Malta | 230.59(196.34,271.81) | 126.08(107.35,148.61) |  | 802.90(681.58,946.19) | 400.53(340.01,472.01) |  | 248.19(213.09,290.54) | 3.67(3.53,3.81) |
| Marshall Islands | 93.61(82.35,106.26) | 581.62(511.67,660.19) |  | 386.35(348.57,429.85) | 1413.03(1274.86,1572.13) |  | 312.71(278.57,346.98) | 2.92(2.78,3.06) |
| Mauritania | 684.91(589.02,806.61) | 90.75(78.05,106.88) |  | 2744.22(2450.90,3059.61) | 160.92(143.72,179.42) |  | 300.67(263.13,347.52) | 1.61(1.47,1.75) |
| Mauritius | 1602.72(1418.89,1792.69) | 294.89(261.07,329.84) |  | 4468.04(3858.47,5044.90) | 701.72(605.99,792.32) |  | 178.78(149.66,211.46) | 2.85(2.61,3.10) |
| Mexico | 152260.81(133056.93,176945.07) | 434.15(379.39,504.53) |  | 404254.72(350618.76,465923.28) | 626.03(542.97,721.53) |  | 165.50(152.92,178.46) | 1.09(0.97,1.21) |
| Micronesia (Federated States of) | 142.00(126.29,160.93) | 373.48(332.16,423.25) |  | 450.41(402.12,505.15) | 938.86(838.20,1052.96) |  | 217.18(189.07,247.01) | 3.24(3.01,3.46) |
| Monaco | 15.37(12.88,18.30) | 106.09(88.91,126.32) |  | 39.36(33.25,46.49) | 254.78(215.23,300.92) |  | 156.11(133.36,181.16) | 2.89(2.85,2.93) |
| Mongolia | 743.27(651.60,854.30) | 87.50(76.71,100.57) |  | 4231.92(3714.61,4730.55) | 259.82(228.06,290.43) |  | 469.37(415.13,528.10) | 3.69(3.61,3.76) |
| Montenegro | 666.68(566.64,770.03) | 220.22(187.17,254.36) |  | 1261.73(1089.37,1462.02) | 430.19(371.42,498.48) |  | 89.26(70.59,111.21) | 2.25(2.13,2.37) |
| Morocco | 27664.11(23896.87,32348.27) | 262.66(226.89,307.13) |  | 171691.42(150033.61,197009.27) | 940.62(821.97,1079.33) |  | 520.63(462.09,590.23) | 4.30(4.23,4.37) |
| Mozambique | 3020.63(2604.64,3521.45) | 62.77(54.13,73.18) |  | 15934.79(13547.83,18529.83) | 138.21(117.51,160.72) |  | 427.53(376.61,486.83) | 2.73(2.67,2.80) |
| Myanmar | 33208.99(28997.01,38578.09) | 190.72(166.53,221.55) |  | 117396.46(104492.70,131191.48) | 431.46(384.03,482.16) |  | 253.51(213.99,291.53) | 2.40(2.30,2.50) |
| Namibia | 529.81(456.03,616.72) | 99.24(85.42,115.52) |  | 2055.86(1776.61,2380.05) | 181.32(156.69,209.91) |  | 288.04(256.41,323.62) | 1.89(1.85,1.94) |
| Nauru | 20.53(18.00,23.55) | 480.51(421.46,551.25) |  | 50.80(45.60,56.82) | 1022.00(917.25,1143.07) |  | 147.48(123.99,173.86) | 2.11(1.89,2.33) |
| Nepal | 14213.76(12305.92,16353.03) | 187.49(162.33,215.71) |  | 59089.18(51783.45,67318.62) | 401.49(351.85,457.41) |  | 315.72(279.51,356.76) | 2.44(2.25,2.64) |
| Netherlands | 9393.31(8111.39,10689.53) | 120.77(104.29,137.44) |  | 18754.37(16009.50,21723.66) | 244.56(208.76,283.28) |  | 99.66(71.47,135.11) | 2.31(2.21,2.42) |
| New Zealand | 3109.66(2606.81,3769.36) | 187.53(157.21,227.31) |  | 6698.68(6005.00,7388.47) | 274.58(246.15,302.85) |  | 115.42(89.95,144.36) | 1.76(1.50,2.02) |
| Nicaragua | 3391.26(2972.46,3835.91) | 244.19(214.03,276.21) |  | 16797.24(14920.08,19030.16) | 515.94(458.28,584.52) |  | 395.31(343.14,453.06) | 2.36(2.31,2.41) |
| Niger | 2954.19(2527.39,3454.56) | 107.77(92.20,126.03) |  | 16445.78(14096.17,19004.20) | 204.06(174.91,235.81) |  | 456.69(411.20,514.68) | 2.14(2.10,2.19) |
| Nigeria | 32199.32(27452.91,37872.92) | 94.05(80.18,110.62) |  | 138467.67(117997.89,162092.97) | 156.65(133.49,183.38) |  | 330.03(314.73,346.06) | 1.63(1.58,1.68) |
| Niue | 4.78(4.22,5.48) | 518.54(458.56,595.25) |  | 9.48(8.23,10.73) | 1259.48(1093.83,1426.23) |  | 98.42(78.09,120.38) | 2.93(2.81,3.05) |
| North Macedonia | 2256.87(1934.26,2683.13) | 233.53(200.15,277.64) |  | 5948.64(5142.70,6828.48) | 523.20(452.32,600.59) |  | 163.58(136.39,199.59) | 2.65(2.47,2.83) |
| Northern Mariana Islands | 70.99(62.25,82.14) | 268.24(235.20,310.39) |  | 155.88(137.13,177.46) | 655.64(576.77,746.42) |  | 119.58(95.05,148.92) | 3.15(2.99,3.32) |
| Norway | 3809.86(3220.00,4560.09) | 185.79(157.02,222.37) |  | 6410.54(5349.17,7695.80) | 250.91(209.37,301.22) |  | 68.26(60.61,75.89) | 0.68(0.55,0.80) |
| Oman | 1977.37(1688.57,2291.90) | 224.29(191.53,259.97) |  | 14576.27(12671.49,17159.10) | 502.70(437.01,591.78) |  | 637.15(559.49,726.91) | 1.55(1.12,1.98) |
| Pakistan | 76777.02(65430.50,90091.21) | 187.06(159.41,219.50) |  | 438417.27(376677.50,509159.36) | 411.64(353.67,478.07) |  | 471.03(440.55,506.91) | 2.73(2.52,2.94) |
| Palau | 36.13(31.65,41.47) | 486.51(426.18,558.44) |  | 117.74(103.75,131.69) | 1241.81(1094.25,1388.96) |  | 225.87(192.99,270.86) | 3.13(3.06,3.21) |
| Palestine | 1423.86(1248.16,1634.92) | 201.05(176.24,230.86) |  | 12206.31(10614.42,13807.15) | 531.29(462.00,600.97) |  | 757.27(660.24,855.62) | 3.31(3.25,3.37) |
| Panama | 2120.55(1859.51,2416.99) | 200.12(175.49,228.10) |  | 9508.32(8307.75,10839.53) | 465.82(407.00,531.04) |  | 348.39(306.46,408.73) | 2.82(2.74,2.90) |
| Papua New Guinea | 5341.01(4685.32,6080.12) | 318.17(279.11,362.20) |  | 34185.79(30593.64,38335.28) | 721.03(645.27,808.55) |  | 540.06(489.83,594.49) | 2.76(2.69,2.83) |
| Paraguay | 3075.50(2656.08,3549.32) | 190.93(164.89,220.35) |  | 14217.11(12652.46,16095.76) | 405.27(360.67,458.82) |  | 362.27(317.89,417.65) | 2.50(2.32,2.68) |
| Peru | 8966.50(7813.71,10244.62) | 99.49(86.70,113.67) |  | 34203.15(29866.56,38709.00) | 188.27(164.40,213.08) |  | 281.46(241.42,330.49) | 2.16(1.97,2.35) |
| Philippines | 33070.16(28201.53,38940.11) | 126.31(107.71,148.73) |  | 117757.67(101319.90,138793.79) | 215.96(185.82,254.54) |  | 256.08(232.30,280.66) | 1.89(1.75,2.03) |
| Poland | 43382.41(37165.04,50728.43) | 240.78(206.28,281.56) |  | 75595.94(64080.98,88253.11) | 410.30(347.80,479.00) |  | 74.25(65.61,83.56) | 1.56(1.47,1.65) |
| Portugal | 9403.98(8139.17,10800.41) | 199.31(172.51,228.91) |  | 21452.42(18339.28,24590.78) | 446.88(382.03,512.26) |  | 128.12(102.47,158.56) | 2.61(2.39,2.82) |
| Puerto Rico | 6533.86(5804.04,7412.08) | 390.44(346.83,442.91) |  | 12177.58(10560.24,14145.78) | 826.44(716.68,960.01) |  | 86.38(67.60,106.89) | 2.48(2.19,2.77) |
| Qatar | 776.86(661.86,904.56) | 282.06(240.31,328.43) |  | 18435.68(15418.92,21463.76) | 827.91(692.43,963.89) |  | 2273.11(1966.91,2657.14) | 2.85(2.50,3.21) |
| Republic of Korea | 60555.81(54498.50,67630.04) | 259.43(233.48,289.74) |  | 180211.27(161090.15,200811.47) | 680.98(608.73,758.83) |  | 197.60(168.96,229.37) | 3.59(3.35,3.83) |
| Republic of Moldova | 3388.29(2997.54,3819.90) | 161.81(143.15,182.42) |  | 6606.80(5744.81,7586.26) | 358.80(311.99,411.99) |  | 94.99(70.90,116.93) | 2.52(2.33,2.71) |
| Romania | 17442.01(15029.09,19865.03) | 160.41(138.22,182.70) |  | 27516.87(23978.10,32534.09) | 309.44(269.65,365.87) |  | 57.76(38.25,77.65) | 2.16(2.08,2.25) |
| Russian Federation | 87777.81(75112.55,104288.60) | 118.24(101.18,140.48) |  | 190078.96(161220.21,223657.84) | 277.25(235.16,326.23) |  | 116.55(104.52,129.74) | 2.82(2.75,2.89) |
| Rwanda | 1728.94(1496.60,1992.28) | 66.94(57.95,77.14) |  | 5086.54(4368.97,5907.56) | 88.09(75.66,102.30) |  | 194.20(171.78,219.26) | 0.50(0.31,0.68) |
| Saint Kitts and Nevis | 53.99(47.14,61.78) | 323.14(282.14,369.75) |  | 237.19(201.24,276.83) | 748.13(634.74,873.16) |  | 339.32(279.56,396.72) | 2.80(2.70,2.90) |
| Saint Lucia | 235.12(202.84,270.25) | 426.22(367.71,489.91) |  | 809.71(711.05,905.78) | 866.25(760.71,969.03) |  | 244.38(203.92,287.14) | 2.48(2.39,2.57) |
| Saint Vincent and the Grenadines | 160.99(142.84,181.51) | 368.11(326.60,415.03) |  | 492.77(422.92,561.15) | 896.83(769.72,1021.29) |  | 206.08(173.97,239.34) | 3.09(2.99,3.19) |
| Samoa | 269.72(239.36,305.73) | 428.63(380.39,485.86) |  | 867.94(774.20,979.31) | 985.44(879.01,1111.89) |  | 221.80(196.29,248.62) | 2.86(2.69,3.03) |
| San Marino | 13.59(11.52,15.71) | 114.25(96.85,132.02) |  | 40.59(34.15,49.48) | 276.63(232.75,337.20) |  | 198.64(170.66,228.35) | 2.98(2.90,3.07) |
| Sao Tome and Principe | 45.30(38.84,52.66) | 112.69(96.62,130.99) |  | 239.47(205.59,276.11) | 248.08(212.98,286.03) |  | 428.62(384.98,476.00) | 2.60(2.54,2.66) |
| Saudi Arabia | 18803.03(16658.82,21187.42) | 278.39(246.65,313.70) |  | 173853.61(151215.37,200960.95) | 707.87(615.69,818.24) |  | 824.60(731.39,929.74) | 2.80(2.73,2.88) |
| Senegal | 3941.56(3379.95,4622.37) | 148.35(127.21,173.97) |  | 18892.64(16332.29,21395.39) | 292.77(253.09,331.55) |  | 379.32(338.80,426.62) | 2.45(2.32,2.57) |
| Serbia | 12242.05(10513.86,14175.62) | 263.34(226.17,304.94) |  | 20298.23(17272.89,23677.89) | 478.68(407.34,558.39) |  | 65.81(41.26,92.43) | 1.95(1.84,2.05) |
| Seychelles | 48.56(41.78,56.89) | 151.58(130.42,177.57) |  | 350.98(300.67,410.46) | 648.04(555.14,757.85) |  | 622.80(532.78,743.62) | 4.80(4.57,5.04) |
| Sierra Leone | 1657.35(1432.74,1942.91) | 102.65(88.74,120.34) |  | 8086.17(6929.71,9471.15) | 217.81(186.66,255.11) |  | 387.90(344.49,434.59) | 2.42(2.35,2.49) |
| Singapore | 6416.00(5730.29,7168.45) | 363.79(324.91,406.45) |  | 17125.10(14725.03,19529.60) | 541.07(465.24,617.04) |  | 166.91(134.64,202.94) | 1.08(1.01,1.15) |
| Slovakia | 4449.69(3844.84,5269.40) | 178.66(154.37,211.57) |  | 8567.40(7432.48,9900.49) | 321.11(278.57,371.07) |  | 92.54(69.65,118.36) | 1.73(1.64,1.81) |
| Slovenia | 1785.59(1540.24,2067.50) | 181.09(156.20,209.68) |  | 2949.62(2505.59,3463.42) | 316.27(268.66,371.36) |  | 65.19(47.35,85.06) | 1.69(1.53,1.85) |
| Solomon Islands | 325.41(293.60,367.73) | 267.09(240.98,301.82) |  | 1721.16(1530.08,1918.09) | 576.46(512.46,642.41) |  | 428.93(387.99,478.61) | 2.53(2.44,2.62) |
| Somalia | 2234.95(1905.21,2607.93) | 78.52(66.94,91.63) |  | 10715.57(9130.23,12561.36) | 136.00(115.88,159.43) |  | 379.45(339.81,423.66) | 1.51(1.43,1.58) |
| South Africa | 21884.54(18481.89,25721.87) | 136.64(115.40,160.60) |  | 76079.70(64843.08,89230.53) | 261.91(223.23,307.18) |  | 247.64(227.09,271.84) | 2.05(2.01,2.10) |
| South Sudan | 1321.39(1125.41,1546.68) | 60.71(51.70,71.06) |  | 4398.15(3838.03,5058.71) | 123.46(107.74,142.00) |  | 232.84(203.27,265.17) | 2.31(2.20,2.42) |
| Spain | 39989.61(34955.35,46165.75) | 221.25(193.40,255.42) |  | 82546.93(71575.87,95161.14) | 388.15(336.56,447.46) |  | 106.42(75.55,137.33) | 1.62(1.50,1.73) |
| Sri Lanka | 15095.82(13059.38,17178.57) | 186.12(161.02,211.80) |  | 60225.18(53140.34,67887.68) | 570.65(503.52,643.25) |  | 298.95(258.11,350.22) | 3.59(3.42,3.77) |
| Sudan | 13487.68(11867.98,15563.49) | 180.12(158.49,207.84) |  | 82667.98(73160.48,93005.21) | 436.70(386.48,491.31) |  | 512.91(461.98,575.33) | 2.85(2.77,2.92) |
| Suriname | 565.10(494.60,640.88) | 328.15(287.21,372.16) |  | 2434.49(2171.65,2753.09) | 876.69(782.04,991.42) |  | 330.81(289.72,384.47) | 3.63(3.47,3.78) |
| Sweden | 6918.30(5744.27,8384.52) | 168.47(139.88,204.17) |  | 13383.08(11117.58,15942.95) | 291.46(242.12,347.21) |  | 93.44(77.71,111.06) | 1.76(1.70,1.81) |
| Switzerland | 6992.58(5927.47,8204.13) | 194.23(164.65,227.89) |  | 16947.02(14445.29,19696.88) | 400.70(341.55,465.71) |  | 142.36(107.68,179.93) | 2.23(2.16,2.30) |
| Syrian Arab Republic | 9289.72(8053.00,10707.53) | 207.18(179.60,238.80) |  | 38628.73(32910.30,45248.01) | 620.28(528.46,726.57) |  | 315.82(270.95,367.88) | 3.62(3.32,3.92) |
| Taiwan (Province of China) | 19634.02(17846.56,21706.96) | 190.47(173.13,210.58) |  | 48781.86(42188.03,55571.09) | 404.45(349.78,460.74) |  | 148.46(119.28,180.61) | 2.54(2.37,2.71) |
| Tajikistan | 1995.51(1764.36,2227.81) | 98.49(87.08,109.95) |  | 12370.31(10904.98,14114.16) | 264.45(233.13,301.74) |  | 519.91(455.73,579.71) | 3.34(3.22,3.46) |
| Thailand | 36386.63(31649.62,41645.81) | 129.57(112.70,148.29) |  | 108064.90(93449.60,123674.42) | 321.65(278.15,368.11) |  | 196.99(160.13,238.01) | 3.09(3.04,3.14) |
| Timor-Leste | 282.80(248.31,323.45) | 85.43(75.02,97.71) |  | 1487.62(1256.90,1703.30) | 260.86(220.40,298.68) |  | 426.03(380.78,477.08) | 3.59(3.51,3.66) |
| Togo | 1036.44(873.60,1221.74) | 79.79(67.26,94.06) |  | 5626.85(4911.34,6489.22) | 157.58(137.54,181.73) |  | 442.90(394.92,507.74) | 2.18(2.09,2.27) |
| Tokelau | 3.21(2.82,3.65) | 535.08(470.84,609.06) |  | 7.12(6.37,7.96) | 1172.93(1050.67,1312.91) |  | 121.81(101.66,145.37) | 2.53(2.35,2.71) |
| Tonga | 151.68(134.17,171.66) | 421.33(372.69,476.82) |  | 406.94(362.92,457.75) | 940.10(838.41,1057.48) |  | 168.29(147.89,193.38) | 2.81(2.75,2.87) |
| Trinidad and Tobago | 2936.00(2651.18,3297.37) | 535.74(483.77,601.68) |  | 6987.77(6080.47,7989.59) | 1026.39(893.12,1173.54) |  | 138.00(104.89,179.18) | 1.86(1.78,1.94) |
| Tunisia | 6594.97(5742.18,7651.62) | 189.04(164.59,219.33) |  | 39163.01(33480.42,46739.89) | 661.55(565.56,789.54) |  | 493.83(431.86,562.20) | 3.97(3.73,4.21) |
| Turkey | 32540.17(28905.08,36879.53) | 131.12(116.47,148.61) |  | 181945.14(159457.55,205452.17) | 429.33(376.27,484.80) |  | 459.14(398.92,534.37) | 4.05(3.66,4.43) |
| Turkmenistan | 1345.10(1202.47,1491.04) | 90.32(80.74,100.12) |  | 6436.78(5750.07,7212.16) | 258.49(230.92,289.63) |  | 378.54(329.06,432.26) | 3.45(3.28,3.62) |
| Tuvalu | 13.68(12.17,15.63) | 335.41(298.57,383.38) |  | 40.31(36.05,45.21) | 715.70(640.05,802.75) |  | 194.76(171.61,219.05) | 2.32(2.20,2.44) |
| Uganda | 3516.28(3007.31,4118.24) | 59.12(50.57,69.25) |  | 17951.90(15425.55,20928.72) | 112.20(96.41,130.80) |  | 410.54(362.45,456.57) | 2.05(2.00,2.10) |
| Ukraine | 31394.93(26915.19,37232.66) | 124.61(106.83,147.78) |  | 55773.21(46896.97,65501.19) | 264.90(222.74,311.10) |  | 77.65(62.46,94.19) | 2.33(2.20,2.45) |
| United Arab Emirates | 2387.03(2041.12,2808.75) | 215.49(184.26,253.56) |  | 53034.81(45091.79,63022.93) | 734.76(624.71,873.14) |  | 2121.79(1852.55,2482.64) | 3.20(2.70,3.70) |
| United Kingdom | 45539.88(38679.29,54349.71) | 164.93(140.08,196.83) |  | 133063.33(116612.44,153626.14) | 426.98(374.20,492.97) |  | 192.19(175.10,210.93) | 3.37(3.13,3.60) |
| United Republic of Tanzania | 4612.64(3954.05,5358.38) | 50.63(43.40,58.82) |  | 24761.73(21634.35,28297.92) | 105.52(92.19,120.58) |  | 436.82(393.12,493.08) | 2.41(2.33,2.49) |
| United States of America | 238.23(205.05,278.26) | 472.87(407.01,552.33) |  | 400.44(350.84,459.04) | 1128.23(988.48,1293.32) |  | 68.09(46.02,93.17) | 2.90(2.78,3.01) |
| United States Virgin Islands | 307187.98(263983.53,356361.96) | 240.77(206.90,279.31) |  | 883785.64(789745.51,995356.52) | 584.97(522.72,658.81) |  | 187.70(165.57,210.62) | 2.89(2.74,3.04) |
| Uruguay | 2071.11(1783.27,2455.39) | 149.25(128.51,176.95) |  | 5942.52(5207.41,6780.24) | 371.87(325.87,424.30) |  | 186.93(153.29,224.95) | 3.29(3.20,3.37) |
| Uzbekistan | 9290.61(8088.87,10404.69) | 111.57(97.14,124.95) |  | 65737.66(58353.94,74142.95) | 387.28(343.78,436.80) |  | 607.57(528.38,685.21) | 4.40(4.11,4.68) |
| Vanuatu | 162.68(142.24,188.29) | 276.61(241.86,320.15) |  | 945.49(852.76,1052.57) | 690.80(623.05,769.04) |  | 481.20(429.17,534.87) | 2.93(2.88,2.97) |
| Venezuela (Bolivarian Republic of) | 17299.14(15310.14,19163.99) | 211.86(187.50,234.70) |  | 59550.96(51807.86,68626.93) | 470.95(409.71,542.73) |  | 244.24(199.26,294.35) | 2.49(2.42,2.56) |
| Viet Nam | 25697.51(22435.72,29509.46) | 93.15(81.32,106.97) |  | 108090.77(95738.87,122044.45) | 211.20(187.06,238.46) |  | 320.63(279.01,368.47) | 2.52(2.38,2.67) |
| Yemen | 6346.09(5534.67,7304.56) | 143.44(125.10,165.10) |  | 44827.02(39067.03,51779.33) | 321.98(280.60,371.91) |  | 606.37(548.29,671.47) | 1.81(1.32,2.31) |
| Zambia | 2707.96(2315.39,3191.33) | 97.56(83.42,114.98) |  | 15482.74(13341.10,17997.15) | 194.00(167.16,225.50) |  | 471.75(416.91,525.80) | 2.18(2.15,2.20) |
| Zimbabwe | 3108.00(2679.75,3588.92) | 84.97(73.27,98.12) |  | 12278.85(10480.32,14267.88) | 188.79(161.14,219.38) |  | 295.07(259.65,337.69) | 2.64(2.60,2.67) |
