# Supplementary material for: Burden of type 2 diabetes in working-age adults (20–54 years): a GBD 2021 analysis projecting trends to 2035 and exploring the potential benefits of physical activity
Source: Front Public Health. 2026 Jan 5;13:1706523. doi: 10.3389/fpubh.2025.1706523 (PMC12812608; doi:10.3389/fpubh.2025.1706523)
Supplement: Supplementary file 2 [file Table_2.docx]

Table S2: Mortality of type 2 diabetes in adults aged 20-54 years between 1990 and 2021 at the national level.

| location | 1990 | |  | 2021 | |  | 1990-2021 | |
| --- | --- | --- | --- | --- | --- | --- | --- | --- |
|  | Death cases | Death rate |  | Death cases | Death rate |  | Cases change | EAPC |
| Afghanistan | 248.85(173.68,369.21) | 7.89(5.50,11.70) |  | 946.73(617.64,1394.01) | 7.75(5.06,11.41) |  | 280.43(149.17,497.77) | -0.09(-0.92,0.76) |
| Albania | 6.10(4.28,7.62) | 0.40(0.28,0.50) |  | 7.04(5.15,9.17) | 0.56(0.41,0.72) |  | 15.45(-22.86,69.78) | 1.79(1.47,2.12) |
| Algeria | 135.65(100.19,186.95) | 1.40(1.04,1.93) |  | 580.70(441.66,763.72) | 2.68(2.04,3.52) |  | 328.09(196.58,510.02) | 2.58(2.32,2.84) |
| American Samoa | 4.08(3.28,5.16) | 19.52(15.68,24.65) |  | 9.25(7.09,11.93) | 41.68(31.94,53.77) |  | 126.47(58.40,225.98) | 2.67(2.27,3.08) |
| Andorra | 0.36(0.23,0.51) | 1.15(0.75,1.65) |  | 0.53(0.32,0.77) | 1.17(0.72,1.72) |  | 47.60(-19.79,160.03) | 0.43(0.15,0.71) |
| Angola | 362.61(264.87,485.93) | 9.33(6.81,12.50) |  | 1019.82(706.78,1359.60) | 8.43(5.84,11.24) |  | 181.25(80.94,324.53) | -0.26(-0.44,-0.09) |
| Antigua and Barbuda | 2.74(2.50,3.00) | 9.89(9.02,10.83) |  | 4.17(3.76,4.68) | 8.83(7.98,9.92) |  | 52.14(30.94,77.31) | -0.20(-0.39,-0.01) |
| Argentina | 523.47(486.00,568.08) | 3.61(3.35,3.91) |  | 620.34(568.08,678.64) | 2.76(2.53,3.02) |  | 18.51(5.25,33.04) | -1.18(-1.41,-0.96) |
| Armenia | 51.88(48.56,55.46) | 3.23(3.02,3.45) |  | 45.34(38.40,53.13) | 3.14(2.66,3.68) |  | -12.60(-28.52,6.32) | 0.74(-0.19,1.68) |
| Australia | 100.98(94.12,108.52) | 1.20(1.12,1.29) |  | 162.25(150.29,175.65) | 1.34(1.24,1.45) |  | 60.68(44.46,77.79) | -0.21(-0.61,0.18) |
| Austria | 57.84(53.98,62.37) | 1.46(1.37,1.58) |  | 33.15(30.74,35.99) | 0.78(0.72,0.84) |  | -42.68(-48.75,-36.58) | -1.09(-1.72,-0.45) |
| Azerbaijan | 85.62(70.08,104.65) | 2.58(2.11,3.15) |  | 187.37(130.34,240.60) | 3.38(2.35,4.34) |  | 118.85(45.03,199.22) | 1.37(0.96,1.78) |
| Bahamas | 12.24(10.95,13.48) | 9.83(8.80,10.83) |  | 20.98(16.24,26.78) | 10.45(8.09,13.34) |  | 71.46(29.30,126.49) | 0.21(0.01,0.41) |
| Bahrain | 18.51(15.55,21.88) | 6.65(5.59,7.86) |  | 90.52(70.83,111.15) | 9.39(7.35,11.53) |  | 388.90(248.52,562.32) | 0.57(0.31,0.83) |
| Bangladesh | 1510.78(1224.58,1852.40) | 3.67(2.97,4.50) |  | 4027.02(2995.74,5399.26) | 5.02(3.73,6.73) |  | 166.55(84.00,269.56) | 1.70(1.41,1.99) |
| Barbados | 13.17(12.23,14.18) | 10.83(10.06,11.66) |  | 16.72(12.72,21.85) | 11.81(8.99,15.44) |  | 26.92(-5.92,65.60) | 0.39(0.01,0.76) |
| Belarus | 43.63(38.40,49.17) | 0.87(0.77,0.99) |  | 63.05(49.94,78.32) | 1.43(1.13,1.77) |  | 44.50(9.44,83.63) | -0.27(-1.22,0.69) |
| Belgium | 47.17(43.53,50.98) | 0.97(0.89,1.04) |  | 34.92(31.85,38.02) | 0.68(0.62,0.74) |  | -25.97(-33.90,-17.45) | -1.55(-2.07,-1.03) |
| Belize | 5.19(4.78,5.61) | 7.53(6.93,8.13) |  | 24.67(21.46,28.13) | 11.75(10.22,13.40) |  | 375.14(297.42,463.84) | 1.48(0.91,2.04) |
| Benin | 70.14(54.52,86.12) | 4.22(3.28,5.18) |  | 273.22(195.48,374.31) | 5.30(3.79,7.26) |  | 289.51(167.17,443.49) | 0.67(0.49,0.85) |
| Bermuda | 1.66(1.53,1.82) | 5.06(4.64,5.52) |  | 1.09(0.86,1.32) | 3.79(3.00,4.59) |  | -34.53(-48.80,-19.55) | -1.00(-1.19,-0.80) |
| Bhutan | 8.00(5.81,10.66) | 3.20(2.33,4.27) |  | 14.89(9.85,21.60) | 3.70(2.45,5.37) |  | 86.08(18.86,194.73) | 0.00(-0.17,0.18) |
| Bolivia (Plurinational State of) | 165.15(131.04,206.07) | 6.59(5.23,8.22) |  | 370.12(263.20,502.88) | 6.49(4.61,8.81) |  | 124.12(51.61,227.30) | -0.38(-0.53,-0.23) |
| Bosnia and Herzegovina | 52.35(45.73,60.28) | 2.29(2.00,2.64) |  | 53.18(39.61,69.10) | 3.45(2.57,4.48) |  | 1.58(-27.75,35.03) | 1.74(1.43,2.06) |
| Botswana | 28.57(19.11,40.38) | 5.88(3.93,8.31) |  | 74.18(52.38,100.96) | 6.01(4.25,8.18) |  | 159.61(61.14,318.24) | 0.06(-0.22,0.35) |
| Brazil | 4298.98(4181.73,4411.07) | 6.46(6.28,6.63) |  | 6331.89(6090.84,6569.21) | 5.60(5.38,5.81) |  | 47.29(40.57,53.70) | -0.76(-0.90,-0.61) |
| Brunei Darussalam | 11.71(9.78,14.02) | 9.11(7.61,10.90) |  | 27.41(22.85,32.40) | 10.46(8.72,12.36) |  | 134.01(83.63,201.74) | 0.63(0.46,0.80) |
| Bulgaria | 138.19(126.50,149.94) | 3.41(3.12,3.70) |  | 126.00(103.92,150.89) | 4.04(3.34,4.84) |  | -8.82(-26.25,12.09) | -0.04(-0.38,0.31) |
| Burkina Faso | 218.71(167.25,289.28) | 6.95(5.31,9.19) |  | 520.08(373.93,683.81) | 6.15(4.42,8.09) |  | 137.79(65.12,241.22) | -0.37(-0.53,-0.22) |
| Burundi | 187.60(131.74,254.05) | 9.28(6.52,12.56) |  | 334.87(233.63,512.41) | 6.51(4.54,9.96) |  | 78.50(19.38,158.63) | -1.89(-2.19,-1.60) |
| Cabo Verde | 2.22(1.77,2.72) | 1.85(1.47,2.27) |  | 14.31(10.41,18.97) | 4.94(3.59,6.54) |  | 545.29(345.16,825.97) | 3.40(2.91,3.89) |
| Cambodia | 237.07(178.98,312.07) | 6.18(4.66,8.13) |  | 538.20(375.23,769.81) | 6.53(4.55,9.34) |  | 127.02(59.41,241.34) | 0.27(0.18,0.37) |
| Cameroon | 244.28(183.37,322.42) | 6.50(4.88,8.58) |  | 1053.63(706.69,1519.58) | 8.20(5.50,11.83) |  | 331.32(189.71,525.91) | 0.56(0.27,0.84) |
| Canada | 203.91(189.82,218.09) | 1.44(1.34,1.54) |  | 271.25(252.25,293.29) | 1.60(1.49,1.73) |  | 33.02(20.92,47.10) | -0.20(-0.77,0.36) |
| Central African Republic | 139.58(105.41,173.76) | 13.36(10.09,16.63) |  | 309.93(211.67,435.83) | 13.88(9.48,19.52) |  | 122.05(48.39,229.04) | 0.18(0.12,0.24) |
| Chad | 75.22(58.54,99.23) | 3.69(2.87,4.87) |  | 322.14(233.63,447.55) | 5.49(3.98,7.62) |  | 328.27(192.13,510.70) | 1.20(0.82,1.58) |
| Chile | 134.47(126.83,143.07) | 2.12(2.00,2.26) |  | 141.72(130.63,153.41) | 1.50(1.39,1.63) |  | 5.40(-4.27,17.38) | -0.75(-1.09,-0.40) |
| China | 9075.91(7722.93,10519.45) | 1.54(1.31,1.79) |  | 13200.41(10507.13,16136.52) | 1.86(1.48,2.27) |  | 45.44(12.91,93.16) | 0.47(0.13,0.81) |
| Colombia | 543.92(515.40,573.11) | 3.74(3.54,3.94) |  | 692.44(564.58,821.75) | 2.78(2.26,3.30) |  | 27.31(4.60,51.77) | -1.76(-2.04,-1.48) |
| Comoros | 13.14(8.27,18.03) | 7.92(4.99,10.87) |  | 28.00(18.31,37.80) | 8.01(5.24,10.81) |  | 113.12(40.94,261.63) | -0.24(-0.65,0.18) |
| Congo | 123.74(91.61,158.62) | 13.79(10.21,17.68) |  | 340.43(246.25,495.86) | 13.78(9.97,20.07) |  | 175.11(86.34,323.22) | -0.23(-0.53,0.07) |
| Cook Islands | 2.62(2.03,3.28) | 32.07(24.84,40.13) |  | 2.87(2.15,3.71) | 36.56(27.37,47.20) |  | 9.44(-26.80,62.46) | 0.67(0.49,0.85) |
| Costa Rica | 35.36(32.78,38.17) | 2.64(2.45,2.85) |  | 86.58(75.32,97.83) | 3.58(3.11,4.04) |  | 144.87(108.26,184.12) | 0.37(-0.27,1.02) |
| Croatia | 46.88(42.90,50.94) | 1.94(1.77,2.10) |  | 32.19(27.11,36.81) | 1.68(1.42,1.93) |  | -31.34(-43.44,-19.48) | -0.22(-0.48,0.03) |
| Cuba | 215.99(201.16,231.18) | 3.93(3.66,4.20) |  | 126.96(107.01,147.87) | 2.33(1.96,2.71) |  | -41.22(-51.86,-30.99) | -1.92(-2.64,-1.21) |
| Cyprus | 12.45(10.07,15.91) | 3.28(2.65,4.19) |  | 12.35(9.64,15.62) | 1.71(1.33,2.16) |  | -0.78(-29.00,38.50) | -2.47(-2.73,-2.20) |
| Czechia | 94.92(84.56,107.37) | 1.94(1.73,2.20) |  | 98.02(79.72,117.47) | 1.99(1.62,2.39) |  | 3.26(-20.50,31.41) | 0.56(0.16,0.97) |
| C么te d'Ivoire | 222.86(170.63,291.07) | 4.80(3.68,6.27) |  | 787.00(564.75,1079.83) | 6.74(4.83,9.24) |  | 253.14(143.77,418.57) | 0.91(0.68,1.14) |
| Democratic People's Republic of Korea | 328.50(225.52,469.63) | 3.24(2.22,4.63) |  | 604.61(396.46,852.37) | 4.27(2.80,6.02) |  | 84.05(19.16,183.05) | 1.16(0.97,1.35) |
| Democratic Republic of the Congo | 1162.88(852.60,1549.86) | 8.39(6.15,11.18) |  | 3067.16(2236.96,4201.84) | 8.50(6.20,11.65) |  | 163.76(78.40,279.41) | 0.16(0.04,0.28) |
| Denmark | 48.94(44.16,54.24) | 1.89(1.71,2.10) |  | 29.97(26.58,33.72) | 1.14(1.01,1.28) |  | -38.77(-48.27,-27.97) | -0.88(-1.54,-0.22) |
| Djibouti | 9.23(6.40,12.92) | 5.47(3.80,7.67) |  | 49.47(33.92,75.90) | 7.89(5.41,12.11) |  | 436.24(243.09,749.84) | 1.06(0.85,1.27) |
| Dominica | 2.88(2.52,3.27) | 9.67(8.44,10.96) |  | 4.76(3.70,5.96) | 14.62(11.38,18.31) |  | 65.12(24.53,120.73) | 1.51(1.34,1.68) |
| Dominican Republic | 137.54(112.62,164.84) | 4.53(3.71,5.43) |  | 441.66(335.27,563.45) | 8.10(6.15,10.34) |  | 221.13(131.38,336.15) | 2.60(2.38,2.83) |
| Ecuador | 177.66(167.07,188.09) | 4.27(4.02,4.52) |  | 472.98(357.51,609.30) | 5.45(4.12,7.02) |  | 166.23(96.66,245.24) | 0.47(-0.13,1.08) |
| Egypt | 1256.33(1087.87,1434.27) | 5.48(4.74,6.25) |  | 3373.43(2588.30,4331.70) | 7.03(5.39,9.02) |  | 168.51(101.34,252.69) | 1.03(0.83,1.23) |
| El Salvador | 114.36(102.80,127.90) | 5.55(4.99,6.20) |  | 360.71(281.38,459.57) | 11.84(9.24,15.09) |  | 215.41(137.90,308.64) | 2.54(2.33,2.75) |
| Equatorial Guinea | 19.34(13.92,26.97) | 12.82(9.23,17.87) |  | 63.06(39.53,94.46) | 9.51(5.96,14.25) |  | 226.02(88.21,431.71) | -0.99(-1.58,-0.40) |
| Eritrea | 140.52(102.45,189.44) | 11.13(8.11,15.01) |  | 290.53(191.61,421.42) | 9.88(6.52,14.33) |  | 106.75(41.16,204.15) | -0.37(-0.48,-0.25) |
| Estonia | 7.98(6.84,8.93) | 1.06(0.91,1.19) |  | 14.34(12.07,16.59) | 2.42(2.03,2.80) |  | 79.67(49.57,116.24) | 1.04(-0.20,2.30) |
| Eswatini | 24.64(18.15,32.23) | 8.67(6.39,11.34) |  | 85.47(53.57,122.98) | 16.14(10.11,23.22) |  | 246.84(118.49,439.87) | 2.38(1.39,3.39) |
| Ethiopia | 2533.60(2065.19,3181.01) | 14.16(11.54,17.78) |  | 2303.49(1886.65,2826.98) | 5.13(4.20,6.30) |  | -9.08(-34.77,22.96) | -4.02(-4.35,-3.69) |
| Fiji | 162.57(135.58,196.64) | 47.35(39.49,57.28) |  | 309.18(231.59,408.92) | 70.73(52.98,93.55) |  | 90.18(32.02,164.54) | 1.34(1.11,1.56) |
| Finland | 20.48(18.72,22.58) | 0.80(0.73,0.88) |  | 14.72(13.23,16.53) | 0.62(0.56,0.70) |  | -28.13(-37.74,-16.59) | -1.19(-1.68,-0.69) |
| France | 247.05(231.18,265.52) | 0.89(0.83,0.96) |  | 247.02(227.33,269.12) | 0.87(0.80,0.94) |  | -0.01(-11.69,12.22) | -0.03(-0.78,0.73) |
| Gabon | 46.31(34.51,63.07) | 12.27(9.14,16.71) |  | 117.08(80.25,169.71) | 14.45(9.90,20.94) |  | 152.82(67.36,291.47) | 0.64(0.44,0.83) |
| Gambia | 14.31(10.24,19.03) | 3.93(2.81,5.22) |  | 61.91(43.50,83.52) | 6.36(4.47,8.58) |  | 332.50(191.51,539.79) | 1.35(1.09,1.61) |
| Georgia | 64.91(56.94,74.66) | 2.48(2.18,2.85) |  | 65.90(56.26,75.88) | 4.05(3.46,4.66) |  | 1.53(-16.16,19.40) | 2.87(2.19,3.56) |
| Germany | 717.13(660.40,781.18) | 1.72(1.59,1.88) |  | 504.40(465.70,553.49) | 1.33(1.23,1.46) |  | -29.66(-37.55,-20.55) | -0.31(-0.58,-0.04) |
| Ghana | 288.49(215.94,376.17) | 5.07(3.79,6.61) |  | 1241.47(906.76,1627.81) | 8.17(5.96,10.71) |  | 330.34(202.78,527.52) | 2.06(1.63,2.48) |
| Greece | 37.78(35.32,40.39) | 0.77(0.72,0.82) |  | 46.71(43.16,50.65) | 1.02(0.94,1.10) |  | 23.65(11.68,38.36) | 0.98(0.86,1.10) |
| Greenland | 0.96(0.78,1.19) | 2.99(2.43,3.69) |  | 0.71(0.54,0.94) | 2.62(1.97,3.47) |  | -26.16(-48.98,5.77) | -0.22(-0.51,0.06) |
| Grenada | 5.29(4.73,5.91) | 16.13(14.41,18.02) |  | 8.99(7.41,10.66) | 17.14(14.13,20.33) |  | 69.95(37.51,108.14) | 0.37(0.15,0.60) |
| Guam | 4.41(3.77,5.01) | 6.32(5.40,7.18) |  | 7.33(6.34,8.63) | 10.03(8.68,11.82) |  | 66.30(35.40,104.59) | 1.82(1.44,2.21) |
| Guatemala | 129.59(122.76,137.24) | 4.47(4.24,4.74) |  | 1308.45(1096.13,1535.34) | 17.87(14.97,20.97) |  | 909.68(738.12,1085.18) | 3.29(2.77,3.82) |
| Guinea | 109.94(78.56,147.41) | 5.11(3.65,6.85) |  | 309.18(221.47,428.52) | 6.12(4.38,8.48) |  | 181.22(83.79,334.76) | 0.66(0.35,0.96) |
| Guinea-Bissau | 29.96(22.17,39.33) | 8.44(6.24,11.07) |  | 77.03(55.41,104.20) | 9.27(6.67,12.55) |  | 157.08(70.13,283.69) | 0.20(0.02,0.39) |
| Guyana | 54.89(47.78,62.98) | 16.30(14.19,18.70) |  | 81.32(59.12,106.95) | 21.90(15.92,28.80) |  | 48.15(3.58,99.91) | 1.11(0.49,1.72) |
| Haiti | 422.16(318.62,549.83) | 16.92(12.77,22.04) |  | 826.79(548.66,1175.66) | 13.62(9.04,19.36) |  | 95.85(26.35,198.13) | -0.57(-0.70,-0.45) |
| Honduras | 65.49(55.34,77.24) | 3.94(3.33,4.64) |  | 239.75(166.74,336.72) | 5.09(3.54,7.15) |  | 266.10(141.27,426.45) | 0.66(0.49,0.82) |
| Hungary | 131.54(121.53,140.22) | 2.68(2.47,2.85) |  | 113.25(98.06,129.08) | 2.49(2.16,2.84) |  | -13.90(-25.71,-0.41) | -0.22(-0.77,0.33) |
| Iceland | 0.51(0.46,0.56) | 0.42(0.38,0.46) |  | 0.53(0.47,0.60) | 0.33(0.29,0.37) |  | 4.65(-10.73,23.21) | -0.68(-1.04,-0.33) |
| India | 14126.90(12541.35,15460.10) | 3.86(3.43,4.22) |  | 33853.90(28885.36,38450.64) | 4.75(4.05,5.39) |  | 139.64(96.97,184.39) | 0.77(0.54,1.00) |
| Indonesia | 3923.51(3255.37,4598.85) | 4.82(4.00,5.65) |  | 10201.04(8054.49,12944.68) | 6.95(5.49,8.82) |  | 160.00(101.36,230.85) | 1.44(1.36,1.51) |
| Iran (Islamic Republic of) | 300.43(256.48,363.58) | 1.43(1.22,1.73) |  | 1209.81(1056.66,1360.72) | 2.60(2.27,2.93) |  | 302.70(197.44,386.11) | 2.54(2.28,2.81) |
| Iraq | 447.44(346.84,579.16) | 6.53(5.06,8.45) |  | 1385.90(960.80,1939.19) | 7.03(4.88,9.84) |  | 209.74(95.22,365.40) | 0.09(-0.03,0.20) |
| Ireland | 11.97(10.95,13.00) | 0.75(0.69,0.82) |  | 10.94(9.83,12.09) | 0.47(0.43,0.52) |  | -8.64(-20.54,4.18) | -1.59(-1.87,-1.32) |
| Israel | 46.65(43.58,49.71) | 2.18(2.03,2.32) |  | 73.45(67.17,79.79) | 1.74(1.59,1.89) |  | 57.46(40.94,75.62) | -0.80(-1.53,-0.06) |
| Italy | 504.67(487.32,519.82) | 1.80(1.74,1.86) |  | 303.24(289.79,318.07) | 1.14(1.09,1.20) |  | -39.91(-42.80,-36.29) | -1.21(-1.38,-1.04) |
| Jamaica | 86.98(80.97,93.43) | 8.89(8.28,9.55) |  | 153.03(111.26,205.70) | 10.51(7.64,14.13) |  | 75.93(23.11,135.36) | 0.21(-0.23,0.65) |
| Japan | 927.55(901.96,953.15) | 1.48(1.44,1.52) |  | 354.21(341.74,365.91) | 0.65(0.63,0.67) |  | -61.81(-63.32,-60.19) | -2.92(-3.39,-2.46) |
| Jordan | 85.44(68.88,105.99) | 6.00(4.84,7.44) |  | 289.83(224.56,363.23) | 4.72(3.66,5.91) |  | 239.24(128.28,378.56) | -1.11(-1.28,-0.94) |
| Kazakhstan | 111.95(98.74,126.03) | 1.46(1.29,1.65) |  | 144.01(117.41,173.31) | 1.59(1.30,1.91) |  | 28.64(4.56,63.53) | -0.95(-1.57,-0.32) |
| Kenya | 282.06(227.08,358.49) | 3.52(2.83,4.47) |  | 1222.50(968.89,1545.46) | 5.63(4.46,7.11) |  | 333.42(241.66,452.00) | 1.70(1.40,2.00) |
| Kiribati | 11.84(9.35,14.66) | 37.23(29.41,46.10) |  | 28.70(20.18,40.90) | 52.05(36.59,74.18) |  | 142.50(66.05,272.52) | 1.00(0.70,1.31) |
| Kuwait | 19.55(17.67,21.60) | 2.08(1.88,2.29) |  | 75.92(61.76,92.28) | 2.46(2.00,2.99) |  | 288.38(203.44,388.01) | 0.06(-0.58,0.70) |
| Kyrgyzstan | 23.19(19.38,27.39) | 1.26(1.05,1.49) |  | 56.49(43.15,72.61) | 1.77(1.35,2.27) |  | 143.56(80.18,228.61) | 0.85(0.52,1.19) |
| Lao People's Democratic Republic | 144.96(104.66,194.26) | 9.30(6.72,12.47) |  | 265.73(195.61,365.11) | 7.36(5.41,10.11) |  | 83.31(23.98,181.56) | -0.93(-1.00,-0.87) |
| Latvia | 22.80(20.85,25.08) | 1.78(1.63,1.96) |  | 35.96(30.84,41.15) | 4.36(3.74,4.99) |  | 57.76(31.47,85.46) | 1.60(0.30,2.91) |
| Lebanon | 87.44(65.87,112.99) | 6.87(5.17,8.87) |  | 112.79(80.43,143.97) | 3.90(2.78,4.97) |  | 28.98(-11.90,88.93) | -1.78(-2.04,-1.51) |
| Lesotho | 27.19(19.58,36.44) | 4.90(3.53,6.56) |  | 103.09(67.45,142.94) | 12.06(7.89,16.72) |  | 279.14(133.49,519.76) | 3.70(2.79,4.63) |
| Liberia | 41.96(30.90,54.25) | 4.63(3.41,5.99) |  | 170.25(112.89,244.48) | 7.28(4.83,10.45) |  | 305.73(163.61,543.64) | 0.88(0.61,1.15) |
| Libya | 29.71(22.47,38.21) | 1.86(1.41,2.39) |  | 195.53(132.10,278.24) | 4.95(3.35,7.05) |  | 558.18(323.73,852.96) | 3.96(3.59,4.34) |
| Lithuania | 16.34(14.51,18.10) | 0.92(0.82,1.02) |  | 36.49(31.44,42.06) | 2.98(2.57,3.43) |  | 123.26(86.28,167.63) | 2.96(1.74,4.19) |
| Luxembourg | 2.22(2.05,2.43) | 1.11(1.03,1.22) |  | 1.85(1.61,2.09) | 0.56(0.49,0.63) |  | -16.58(-28.62,-3.23) | -2.01(-2.35,-1.66) |
| Madagascar | 268.79(210.58,335.30) | 6.21(4.86,7.74) |  | 724.38(509.61,1013.89) | 6.16(4.33,8.62) |  | 169.49(89.52,281.67) | 0.12(0.03,0.22) |
| Malawi | 248.93(200.55,311.22) | 6.91(5.57,8.64) |  | 609.07(461.45,786.18) | 7.92(6.00,10.23) |  | 144.68(76.08,242.08) | -0.03(-0.37,0.32) |
| Malaysia | 363.11(303.74,424.28) | 4.63(3.87,5.41) |  | 751.16(641.32,872.98) | 4.51(3.85,5.24) |  | 106.87(64.91,157.10) | -0.14(-0.40,0.12) |
| Maldives | 5.93(4.63,7.40) | 7.55(5.90,9.42) |  | 8.55(6.26,10.99) | 2.57(1.88,3.30) |  | 44.12(1.01,106.47) | -3.75(-4.19,-3.30) |
| Mali | 185.01(145.32,236.01) | 6.07(4.77,7.74) |  | 508.99(380.87,666.25) | 6.04(4.52,7.91) |  | 175.12(88.94,288.12) | 0.01(-0.08,0.11) |
| Malta | 3.36(3.06,3.68) | 1.84(1.67,2.01) |  | 3.04(2.70,3.47) | 1.52(1.35,1.73) |  | -9.46(-22.23,3.76) | -0.80(-1.19,-0.41) |
| Marshall Islands | 3.41(2.65,4.46) | 21.16(16.46,27.70) |  | 14.56(9.06,21.81) | 53.24(33.14,79.76) |  | 327.37(168.41,509.80) | 2.80(2.34,3.26) |
| Mauritania | 34.50(26.62,42.97) | 4.57(3.53,5.69) |  | 82.80(61.02,117.00) | 4.86(3.58,6.86) |  | 139.97(66.37,244.70) | -0.00(-0.11,0.11) |
| Mauritius | 72.21(67.16,78.23) | 13.29(12.36,14.39) |  | 214.18(194.38,230.93) | 33.64(30.53,36.27) |  | 196.61(162.48,229.02) | 4.90(4.05,5.75) |
| Mexico | 4653.56(4543.50,4772.10) | 13.27(12.96,13.61) |  | 11939.53(10486.81,13568.48) | 18.49(16.24,21.01) |  | 156.57(126.31,193.03) | 0.90(0.42,1.38) |
| Micronesia (Federated States of) | 7.74(5.53,10.58) | 20.36(14.53,27.82) |  | 17.85(12.97,25.17) | 37.21(27.03,52.46) |  | 130.60(59.22,237.27) | 2.00(1.60,2.39) |
| Monaco | 0.08(0.06,0.10) | 0.55(0.43,0.69) |  | 0.11(0.08,0.15) | 0.70(0.50,0.95) |  | 34.75(-9.51,99.22) | 0.80(0.65,0.94) |
| Mongolia | 8.74(6.13,12.02) | 1.03(0.72,1.41) |  | 41.94(31.15,54.22) | 2.58(1.91,3.33) |  | 379.95(213.62,657.80) | 3.59(3.26,3.92) |
| Montenegro | 5.62(4.35,7.11) | 1.86(1.44,2.35) |  | 6.26(4.76,8.13) | 2.13(1.62,2.77) |  | 11.28(-23.10,59.56) | 1.02(0.56,1.48) |
| Morocco | 185.41(138.02,241.45) | 1.76(1.31,2.29) |  | 746.01(487.35,1048.53) | 4.09(2.67,5.74) |  | 302.36(157.97,501.91) | 3.27(3.03,3.51) |
| Mozambique | 385.34(303.91,484.23) | 8.01(6.32,10.06) |  | 1210.69(844.96,1646.47) | 10.50(7.33,14.28) |  | 214.19(111.80,359.40) | 1.72(1.39,2.04) |
| Myanmar | 2609.36(1899.01,3457.58) | 14.99(10.91,19.86) |  | 3785.54(2830.07,5035.91) | 13.91(10.40,18.51) |  | 45.08(-4.76,122.50) | -0.39(-0.50,-0.29) |
| Namibia | 30.55(23.59,39.49) | 5.72(4.42,7.40) |  | 88.70(59.65,126.36) | 7.82(5.26,11.14) |  | 190.31(88.18,336.93) | 0.62(0.15,1.09) |
| Nauru | 1.41(1.05,1.87) | 33.06(24.63,43.86) |  | 2.12(1.50,2.86) | 42.71(30.14,57.51) |  | 50.30(8.06,125.54) | 0.60(0.34,0.86) |
| Nepal | 266.76(195.48,341.24) | 3.52(2.58,4.50) |  | 659.57(457.56,911.23) | 4.48(3.11,6.19) |  | 147.25(65.92,267.88) | 0.94(0.58,1.31) |
| Netherlands | 124.96(116.03,134.70) | 1.61(1.49,1.73) |  | 68.69(62.28,75.39) | 0.90(0.81,0.98) |  | -45.03(-51.66,-38.24) | -2.10(-2.57,-1.64) |
| New Zealand | 35.76(32.11,39.21) | 2.16(1.94,2.36) |  | 43.74(40.34,47.44) | 1.79(1.65,1.94) |  | 22.32(7.97,38.53) | -1.06(-1.51,-0.61) |
| Nicaragua | 66.37(58.43,74.47) | 4.78(4.21,5.36) |  | 211.22(171.52,257.18) | 6.49(5.27,7.90) |  | 218.23(154.77,309.88) | 1.00(0.82,1.19) |
| Niger | 94.47(70.60,124.88) | 3.45(2.58,4.56) |  | 275.89(189.90,397.17) | 3.42(2.36,4.93) |  | 192.03(99.85,320.97) | 0.14(0.01,0.26) |
| Nigeria | 1878.63(1451.49,2485.02) | 5.49(4.24,7.26) |  | 4445.76(3117.39,6069.73) | 5.03(3.53,6.87) |  | 136.65(60.52,247.48) | -0.43(-0.54,-0.33) |
| Niue | 0.22(0.16,0.30) | 23.65(17.53,32.71) |  | 0.29(0.21,0.41) | 38.23(27.53,54.37) |  | 32.06(-14.03,110.53) | 1.39(1.19,1.59) |
| North Macedonia | 27.10(22.13,33.20) | 2.80(2.29,3.44) |  | 35.83(26.35,47.54) | 3.15(2.32,4.18) |  | 32.21(-6.43,79.76) | 0.40(-0.07,0.86) |
| Northern Mariana Islands | 3.36(2.40,4.53) | 12.68(9.07,17.10) |  | 5.15(4.14,6.19) | 21.65(17.43,26.02) |  | 53.37(9.68,125.28) | 2.83(2.13,3.55) |
| Norway | 19.55(18.72,20.44) | 0.95(0.91,1.00) |  | 18.91(18.00,19.81) | 0.74(0.70,0.78) |  | -3.31(-9.74,2.66) | -1.28(-1.93,-0.63) |
| Oman | 33.45(23.05,48.81) | 3.79(2.61,5.54) |  | 116.64(88.22,150.90) | 4.02(3.04,5.20) |  | 248.71(104.46,453.85) | -0.07(-0.38,0.24) |
| Pakistan | 1753.42(1338.64,2211.64) | 4.27(3.26,5.39) |  | 7005.94(5281.40,9073.38) | 6.58(4.96,8.52) |  | 299.56(198.20,427.61) | 0.98(0.71,1.26) |
| Palau | 1.40(1.02,1.83) | 18.82(13.71,24.66) |  | 3.90(2.97,5.17) | 41.16(31.30,54.54) |  | 179.23(89.04,327.03) | 2.62(2.34,2.89) |
| Palestine | 31.33(22.78,42.68) | 4.42(3.22,6.03) |  | 104.79(85.12,130.13) | 4.56(3.70,5.66) |  | 234.45(127.73,398.42) | 0.24(0.15,0.33) |
| Panama | 40.52(37.90,43.64) | 3.82(3.58,4.12) |  | 122.71(95.49,151.30) | 6.01(4.68,7.41) |  | 202.83(134.69,280.33) | 1.52(1.21,1.83) |
| Papua New Guinea | 371.62(242.02,507.99) | 22.14(14.42,30.26) |  | 1081.77(804.83,1414.24) | 22.82(16.98,29.83) |  | 191.09(82.48,359.19) | 0.12(0.02,0.21) |
| Paraguay | 72.00(61.97,83.85) | 4.47(3.85,5.21) |  | 318.63(237.47,422.69) | 9.08(6.77,12.05) |  | 342.52(220.32,506.95) | 2.54(2.15,2.93) |
| Peru | 239.42(196.98,287.28) | 2.66(2.19,3.19) |  | 690.83(505.77,943.12) | 3.80(2.78,5.19) |  | 188.54(96.84,311.47) | 1.15(0.85,1.44) |
| Philippines | 1815.38(1645.54,1995.86) | 6.93(6.29,7.62) |  | 5675.98(4736.19,6712.04) | 10.41(8.69,12.31) |  | 212.66(155.62,274.67) | 1.44(1.32,1.56) |
| Poland | 487.34(468.59,506.59) | 2.70(2.60,2.81) |  | 460.27(416.69,503.39) | 2.50(2.26,2.73) |  | -5.55(-15.02,4.30) | 0.15(-0.28,0.57) |
| Portugal | 128.26(120.41,136.35) | 2.72(2.55,2.89) |  | 67.64(62.53,73.63) | 1.41(1.30,1.53) |  | -47.26(-52.46,-41.43) | -2.12(-2.43,-1.81) |
| Puerto Rico | 155.11(146.02,164.63) | 9.27(8.73,9.84) |  | 172.43(141.08,207.95) | 11.70(9.57,14.11) |  | 11.16(-11.04,34.24) | 0.13(-0.19,0.45) |
| Qatar | 11.72(8.67,15.43) | 4.26(3.15,5.60) |  | 88.89(64.96,119.25) | 3.99(2.92,5.36) |  | 658.37(367.32,1058.91) | -0.96(-1.59,-0.32) |
| Republic of Korea | 1109.52(984.02,1257.98) | 4.75(4.22,5.39) |  | 552.85(466.54,642.73) | 2.09(1.76,2.43) |  | -50.17(-59.57,-39.83) | -2.81(-3.25,-2.37) |
| Republic of Moldova | 34.12(31.35,37.04) | 1.63(1.50,1.77) |  | 50.09(44.32,56.92) | 2.72(2.41,3.09) |  | 46.79(28.18,68.55) | 0.42(-0.59,1.43) |
| Romania | 194.71(171.89,218.30) | 1.79(1.58,2.01) |  | 144.34(122.26,169.55) | 1.62(1.37,1.91) |  | -25.87(-39.59,-9.63) | -0.68(-1.09,-0.27) |
| Russian Federation | 644.76(601.39,671.23) | 0.87(0.81,0.90) |  | 1506.57(1387.28,1627.38) | 2.20(2.02,2.37) |  | 133.66(111.30,161.21) | 1.74(0.83,2.66) |
| Rwanda | 285.92(199.46,403.73) | 11.07(7.72,15.63) |  | 362.64(206.50,577.32) | 6.28(3.58,10.00) |  | 26.83(-19.74,91.06) | -3.21(-3.75,-2.67) |
| Saint Kitts and Nevis | 1.78(1.63,1.94) | 10.64(9.73,11.58) |  | 2.95(2.27,3.74) | 9.30(7.16,11.80) |  | 65.92(25.62,110.13) | -0.44(-0.70,-0.17) |
| Saint Lucia | 8.44(7.83,9.10) | 15.30(14.20,16.50) |  | 13.06(10.45,16.09) | 13.97(11.18,17.21) |  | 54.75(23.84,92.10) | -0.33(-0.50,-0.16) |
| Saint Vincent and the Grenadines | 6.92(6.31,7.55) | 15.83(14.44,17.26) |  | 10.86(9.26,12.60) | 19.77(16.86,22.94) |  | 56.87(30.28,88.10) | 0.80(0.59,1.03) |
| Samoa | 9.71(7.36,12.66) | 15.43(11.69,20.12) |  | 22.23(16.31,29.56) | 25.24(18.52,33.56) |  | 129.03(52.14,243.89) | 1.90(1.80,2.00) |
| San Marino | 0.08(0.06,0.10) | 0.65(0.53,0.81) |  | 0.08(0.04,0.12) | 0.52(0.28,0.79) |  | -1.38(-46.65,64.21) | 0.29(-0.05,0.63) |
| Sao Tome and Principe | 0.80(0.61,1.00) | 1.99(1.53,2.48) |  | 2.59(1.83,3.57) | 2.68(1.89,3.70) |  | 223.99(106.15,387.31) | 1.13(0.97,1.29) |
| Saudi Arabia | 228.59(157.27,323.81) | 3.38(2.33,4.79) |  | 1699.65(1225.09,2259.47) | 6.92(4.99,9.20) |  | 643.55(310.39,1151.22) | 2.16(2.05,2.27) |
| Senegal | 135.44(104.90,172.73) | 5.10(3.95,6.50) |  | 426.83(311.24,601.83) | 6.61(4.82,9.33) |  | 215.15(116.22,359.15) | 0.99(0.77,1.21) |
| Serbia | 130.87(105.53,158.28) | 2.82(2.27,3.40) |  | 114.45(88.58,141.30) | 2.70(2.09,3.33) |  | -12.54(-34.96,18.08) | -0.15(-0.52,0.22) |
| Seychelles | 1.03(0.88,1.18) | 3.20(2.76,3.69) |  | 3.43(2.86,4.12) | 6.34(5.27,7.60) |  | 234.57(162.64,326.18) | 3.05(2.83,3.27) |
| Sierra Leone | 56.43(40.64,75.12) | 3.50(2.52,4.65) |  | 181.55(130.03,245.22) | 4.89(3.50,6.61) |  | 221.74(120.77,360.38) | 1.20(0.95,1.46) |
| Singapore | 44.48(41.12,48.30) | 2.52(2.33,2.74) |  | 13.06(12.12,14.07) | 0.41(0.38,0.44) |  | -70.65(-73.75,-67.44) | -5.97(-6.87,-5.06) |
| Slovakia | 44.46(38.00,51.16) | 1.79(1.53,2.05) |  | 38.14(30.07,47.57) | 1.43(1.13,1.78) |  | -14.21(-36.12,13.88) | -0.50(-0.84,-0.16) |
| Slovenia | 17.21(15.62,18.97) | 1.75(1.58,1.92) |  | 13.34(11.09,15.77) | 1.43(1.19,1.69) |  | -22.48(-36.89,-4.55) | -1.14(-1.40,-0.87) |
| Solomon Islands | 29.63(15.41,44.95) | 24.32(12.65,36.89) |  | 122.99(90.10,166.71) | 41.19(30.18,55.83) |  | 315.11(162.37,687.50) | 1.90(1.73,2.08) |
| Somalia | 292.79(204.10,416.93) | 10.29(7.17,14.65) |  | 681.50(463.24,941.16) | 8.65(5.88,11.95) |  | 132.76(54.59,258.28) | -1.22(-1.45,-1.00) |
| South Africa | 1297.98(1191.22,1421.66) | 8.10(7.44,8.88) |  | 3332.28(2981.22,3678.67) | 11.47(10.26,12.66) |  | 156.73(118.26,192.57) | 1.71(1.35,2.06) |
| South Sudan | 153.75(109.03,220.36) | 7.06(5.01,10.12) |  | 396.93(275.09,552.73) | 11.14(7.72,15.52) |  | 158.17(71.85,288.66) | 1.39(0.84,1.94) |
| Spain | 262.13(245.40,280.26) | 1.45(1.36,1.55) |  | 152.20(139.26,166.77) | 0.72(0.65,0.78) |  | -41.94(-48.01,-34.97) | -2.16(-2.33,-1.99) |
| Sri Lanka | 463.44(388.50,549.23) | 5.71(4.79,6.77) |  | 983.62(611.23,1404.16) | 9.32(5.79,13.30) |  | 112.24(24.55,227.36) | 2.23(1.85,2.62) |
| Sudan | 150.36(108.21,209.17) | 2.01(1.45,2.79) |  | 511.31(331.30,738.84) | 2.70(1.75,3.90) |  | 240.05(109.77,414.94) | 1.20(0.95,1.46) |
| Suriname | 15.08(12.87,17.26) | 8.76(7.47,10.03) |  | 36.04(27.11,46.27) | 12.98(9.76,16.66) |  | 138.91(71.67,231.31) | 1.49(1.15,1.83) |
| Sweden | 60.73(56.65,64.60) | 1.48(1.38,1.57) |  | 39.62(34.19,45.21) | 0.86(0.74,0.98) |  | -34.76(-43.96,-24.99) | -1.64(-1.95,-1.32) |
| Switzerland | 46.85(43.26,50.69) | 1.30(1.20,1.41) |  | 25.34(22.85,28.03) | 0.60(0.54,0.66) |  | -45.90(-52.32,-39.19) | -2.43(-2.73,-2.12) |
| Syrian Arab Republic | 122.82(94.63,158.10) | 2.74(2.11,3.53) |  | 238.56(166.03,340.63) | 3.83(2.67,5.47) |  | 94.23(23.07,205.87) | 0.76(-0.12,1.64) |
| Taiwan (Province of China) | 407.71(381.76,437.33) | 3.96(3.70,4.24) |  | 515.57(463.84,571.76) | 4.27(3.85,4.74) |  | 26.46(11.51,42.07) | -0.51(-1.11,0.10) |
| Tajikistan | 59.89(47.20,76.15) | 2.96(2.33,3.76) |  | 143.53(100.36,197.87) | 3.07(2.15,4.23) |  | 139.67(61.12,256.53) | -0.12(-0.36,0.12) |
| Thailand | 1400.26(1097.29,1756.36) | 4.99(3.91,6.25) |  | 2831.95(2047.95,3811.91) | 8.43(6.10,11.35) |  | 102.24(38.29,209.26) | 1.19(0.80,1.59) |
| Timor-Leste | 11.30(7.92,15.39) | 3.41(2.39,4.65) |  | 21.64(15.31,30.28) | 3.80(2.68,5.31) |  | 91.62(26.28,185.06) | 0.39(0.05,0.72) |
| Togo | 49.35(38.30,62.91) | 3.80(2.95,4.84) |  | 222.22(145.81,316.70) | 6.22(4.08,8.87) |  | 350.29(197.98,568.62) | 1.64(1.42,1.87) |
| Tokelau | 0.12(0.08,0.17) | 19.30(13.57,27.67) |  | 0.16(0.11,0.21) | 25.70(18.89,34.40) |  | 34.76(-10.53,106.02) | 0.99(0.76,1.22) |
| Tonga | 7.88(6.34,9.70) | 21.89(17.62,26.94) |  | 12.51(9.03,16.88) | 28.90(20.87,39.01) |  | 58.72(6.00,133.98) | 0.89(0.82,0.96) |
| Trinidad and Tobago | 151.91(143.77,161.16) | 27.72(26.23,29.41) |  | 165.74(122.71,216.64) | 24.34(18.02,31.82) |  | 9.10(-20.02,44.47) | -0.98(-1.25,-0.71) |
| Tunisia | 43.06(32.34,54.46) | 1.23(0.93,1.56) |  | 159.88(106.24,228.03) | 2.70(1.79,3.85) |  | 271.25(123.52,489.93) | 3.02(2.84,3.21) |
| Turkey | 1064.77(861.07,1338.90) | 4.29(3.47,5.40) |  | 1256.36(963.40,1633.72) | 2.96(2.27,3.86) |  | 17.99(-18.45,61.77) | -0.94(-1.24,-0.64) |
| Turkmenistan | 32.33(27.84,37.11) | 2.17(1.87,2.49) |  | 163.27(122.80,219.09) | 6.56(4.93,8.80) |  | 405.03(270.31,576.53) | 3.79(3.34,4.24) |
| Tuvalu | 1.01(0.75,1.33) | 24.69(18.41,32.62) |  | 1.60(1.20,2.15) | 28.47(21.23,38.14) |  | 59.26(18.71,112.33) | 0.41(0.15,0.67) |
| Uganda | 345.12(224.34,585.30) | 5.80(3.77,9.84) |  | 1065.88(708.57,1688.41) | 6.66(4.43,10.55) |  | 208.84(101.64,372.92) | -0.18(-0.45,0.08) |
| Ukraine | 366.40(320.26,410.65) | 1.45(1.27,1.63) |  | 256.94(182.85,335.71) | 1.22(0.87,1.59) |  | -29.88(-50.60,-6.10) | -2.33(-2.98,-1.68) |
| United Arab Emirates | 31.21(21.86,46.67) | 2.82(1.97,4.21) |  | 212.92(145.94,281.87) | 2.95(2.02,3.91) |  | 582.24(255.81,872.05) | -0.77(-1.22,-0.32) |
| United Kingdom | 315.46(310.73,320.78) | 1.14(1.13,1.16) |  | 245.80(239.60,252.48) | 0.79(0.77,0.81) |  | -22.08(-24.68,-19.67) | -1.11(-1.39,-0.82) |
| United Republic of Tanzania | 562.29(452.22,706.45) | 6.17(4.96,7.75) |  | 1473.00(1045.79,1994.71) | 6.28(4.46,8.50) |  | 161.97(72.46,276.31) | -0.06(-0.12,-0.00) |
| United States of America | 4.51(3.64,5.45) | 8.95(7.23,10.81) |  | 3.04(2.24,3.98) | 8.56(6.30,11.23) |  | -32.61(-53.16,-4.28) | 0.03(-0.28,0.34) |
| United States Virgin Islands | 3931.37(3824.53,4056.27) | 3.08(3.00,3.18) |  | 5691.24(5508.22,5881.98) | 3.77(3.65,3.89) |  | 44.76(38.34,50.94) | 0.25(-0.19,0.69) |
| Uruguay | 34.20(31.75,37.01) | 2.46(2.29,2.67) |  | 40.69(37.42,44.57) | 2.55(2.34,2.79) |  | 18.98(4.96,33.91) | -0.19(-0.35,-0.02) |
| Uzbekistan | 201.83(180.68,226.85) | 2.42(2.17,2.72) |  | 883.41(726.25,1061.11) | 5.20(4.28,6.25) |  | 337.71(252.10,436.17) | 1.87(1.44,2.30) |
| Vanuatu | 9.50(6.33,15.06) | 16.14(10.76,25.60) |  | 34.41(25.47,46.70) | 25.14(18.61,34.12) |  | 262.42(134.97,468.09) | 1.19(1.09,1.28) |
| Venezuela (Bolivarian Republic of) | 473.85(446.87,501.08) | 5.80(5.47,6.14) |  | 1290.17(961.35,1701.43) | 10.20(7.60,13.46) |  | 172.27(97.67,258.86) | 1.18(0.69,1.68) |
| Viet Nam | 1030.51(750.35,1375.45) | 3.74(2.72,4.99) |  | 2963.77(2101.83,4010.12) | 5.79(4.11,7.84) |  | 187.60(91.62,337.88) | 1.96(1.74,2.18) |
| Yemen | 77.10(50.02,121.50) | 1.74(1.13,2.75) |  | 252.80(155.57,419.76) | 1.82(1.12,3.02) |  | 227.91(107.73,402.77) | -0.08(-0.26,0.10) |
| Zambia | 231.34(179.44,290.59) | 8.33(6.46,10.47) |  | 666.72(465.75,922.27) | 8.35(5.84,11.56) |  | 188.20(88.91,320.74) | -0.50(-0.69,-0.31) |
| Zimbabwe | 124.65(96.07,160.88) | 3.41(2.63,4.40) |  | 533.81(382.40,725.13) | 8.21(5.88,11.15) |  | 328.24(186.58,549.48) | 3.54(2.61,4.48) |
