# Supplementary material for: Burden of type 2 diabetes in working-age adults (20–54 years): a GBD 2021 analysis projecting trends to 2035 and exploring the potential benefits of physical activity
Source: Front Public Health. 2026 Jan 5;13:1706523. doi: 10.3389/fpubh.2025.1706523 (PMC12812608; doi:10.3389/fpubh.2025.1706523)
Supplement: Supplementary file 3 [file Table_3.docx]

Table S3: DALYs of type 2 diabetes in adults aged 20-54 years between 1990 and 2021 at the national level.

| location | 1990 | |  | 2021 | |  | 1990-2021 | |
| --- | --- | --- | --- | --- | --- | --- | --- | --- |
|  | DALY cases | DALY rate |  | DALY cases | DALY rate |  | Cases change | EAPC |
| Afghanistan | 20392.19(15660.11,26614.04) | 646.28(496.31,843.47) |  | 134962.97(99996.86,180353.45) | 1104.74(818.52,1476.28) |  | 561.84(423.01,700.55) | 2.20(1.59,2.81) |
| Albania | 1762.23(1216.09,2495.70) | 116.53(80.42,165.03) |  | 2942.99(2005.47,4233.08) | 232.75(158.60,334.78) |  | 67.00(49.97,83.48) | 2.50(2.23,2.78) |
| Algeria | 20695.36(15529.44,27429.37) | 213.87(160.49,283.47) |  | 143404.63(103402.43,195545.43) | 661.59(477.04,902.14) |  | 592.93(494.38,688.83) | 3.74(3.67,3.82) |
| American Samoa | 290.60(238.11,352.54) | 1389.14(1138.21,1685.24) |  | 804.75(644.18,1027.38) | 3627.65(2903.85,4631.25) |  | 176.92(123.75,237.50) | 3.24(2.91,3.57) |
| Andorra | 50.47(37.18,67.81) | 162.60(119.80,218.50) |  | 164.34(113.34,225.32) | 367.22(253.26,503.46) |  | 225.65(171.30,277.12) | 2.80(2.70,2.89) |
| Angola | 22901.31(17776.69,28900.74) | 589.13(457.30,743.46) |  | 92316.01(72103.10,118643.47) | 763.12(596.04,980.76) |  | 303.10(199.92,416.21) | 0.95(0.81,1.10) |
| Antigua and Barbuda | 205.25(174.88,247.46) | 741.05(631.42,893.46) |  | 533.41(399.82,692.19) | 1130.96(847.72,1467.62) |  | 159.89(126.23,190.64) | 1.49(1.40,1.59) |
| Argentina | 37929.17(32383.54,44836.98) | 261.23(223.03,308.80) |  | 82315.78(62602.26,108532.86) | 366.79(278.95,483.61) |  | 117.03(88.79,146.77) | 0.88(0.77,0.99) |
| Armenia | 4556.23(3697.11,5621.81) | 283.70(230.20,350.04) |  | 6371.50(4888.17,8401.65) | 440.77(338.15,581.21) |  | 39.84(24.05,55.94) | 1.63(1.08,2.19) |
| Australia | 10710.87(8443.37,13648.31) | 127.43(100.45,162.38) |  | 26792.96(19627.89,35538.89) | 220.59(161.60,292.60) |  | 150.15(118.77,185.94) | 1.66(1.48,1.84) |
| Austria | 4995.20(4090.42,6183.57) | 126.49(103.58,156.58) |  | 9123.58(6435.94,12678.48) | 213.55(150.65,296.76) |  | 82.65(54.74,110.95) | 1.98(1.78,2.17) |
| Azerbaijan | 7090.07(5754.81,8878.24) | 213.29(173.12,267.08) |  | 24941.35(18383.32,33316.74) | 450.40(331.97,601.65) |  | 251.78(194.16,307.01) | 2.70(2.42,2.98) |
| Bahamas | 901.65(760.53,1089.87) | 724.30(610.94,875.50) |  | 2444.56(1864.22,3174.41) | 1217.77(928.67,1581.35) |  | 171.12(132.01,210.34) | 1.74(1.63,1.85) |
| Bahrain | 1367.95(1138.70,1656.82) | 491.52(409.15,595.32) |  | 10838.15(8335.64,13892.15) | 1124.63(864.96,1441.53) |  | 692.29(540.34,818.19) | 2.30(2.12,2.49) |
| Bangladesh | 134372.67(108308.23,163601.22) | 326.25(262.96,397.21) |  | 554378.19(422862.78,720022.85) | 690.55(526.73,896.88) |  | 312.57(243.86,380.10) | 2.83(2.68,2.99) |
| Barbados | 932.69(805.49,1097.76) | 766.86(662.27,902.57) |  | 1802.75(1394.50,2325.59) | 1273.80(985.33,1643.23) |  | 93.28(62.81,122.71) | 1.57(1.36,1.78) |
| Belarus | 6654.72(4877.48,8770.83) | 133.32(97.72,175.72) |  | 11333.20(8279.75,15374.40) | 256.25(187.21,347.62) |  | 70.30(53.23,90.18) | 1.53(1.29,1.77) |
| Belgium | 9112.89(6606.71,12185.38) | 186.54(135.24,249.43) |  | 19238.21(12939.55,27570.72) | 373.98(251.53,535.95) |  | 111.11(89.98,131.17) | 2.25(2.04,2.47) |
| Belize | 377.65(327.75,440.37) | 547.69(475.33,638.66) |  | 2262.04(1828.65,2805.07) | 1077.47(871.04,1336.14) |  | 498.98(434.30,567.43) | 2.22(1.85,2.59) |
| Benin | 5442.44(4395.73,6566.82) | 327.66(264.64,395.35) |  | 31416.14(24045.52,40903.80) | 609.33(466.38,793.35) |  | 477.24(367.76,588.74) | 1.88(1.80,1.96) |
| Bermuda | 138.12(112.91,169.66) | 419.79(343.16,515.64) |  | 200.44(145.84,270.29) | 697.12(507.21,940.05) |  | 45.12(23.01,65.84) | 1.59(1.54,1.65) |
| Bhutan | 703.21(547.10,883.51) | 281.56(219.05,353.75) |  | 1927.33(1453.87,2537.47) | 479.04(361.36,630.69) |  | 174.08(123.11,236.54) | 1.38(1.25,1.51) |
| Bolivia (Plurinational State of) | 10372.03(8530.64,12575.22) | 413.58(340.16,501.43) |  | 32286.67(25292.12,41484.31) | 565.95(443.34,727.17) |  | 211.29(145.44,294.73) | 0.78(0.67,0.89) |
| Bosnia and Herzegovina | 5500.06(4296.36,7131.40) | 240.92(188.19,312.37) |  | 8235.47(6017.24,11447.98) | 533.90(390.09,742.16) |  | 49.73(28.17,71.34) | 2.80(2.57,3.02) |
| Botswana | 1689.42(1258.99,2243.16) | 347.77(259.16,461.76) |  | 5967.84(4626.43,7601.43) | 483.69(374.97,616.09) |  | 253.25(151.45,377.92) | 0.99(0.82,1.16) |
| Brazil | 324785.11(284958.02,372796.53) | 488.12(428.27,560.28) |  | 652885.82(528064.72,804960.56) | 577.10(466.77,711.53) |  | 101.02(85.44,116.39) | 0.43(0.33,0.53) |
| Brunei Darussalam | 751.26(635.24,884.29) | 584.29(494.06,687.76) |  | 3158.94(2463.07,4071.80) | 1205.13(939.66,1553.39) |  | 320.49(241.63,411.59) | 2.45(2.28,2.61) |
| Bulgaria | 12887.47(10268.90,16409.97) | 317.94(253.34,404.84) |  | 17034.44(12867.82,22812.08) | 546.72(412.99,732.16) |  | 32.18(17.23,47.73) | 1.51(1.35,1.67) |
| Burkina Faso | 12546.77(10160.10,15595.69) | 398.61(322.78,495.47) |  | 42629.11(33726.07,54369.65) | 504.37(399.04,643.28) |  | 239.76(159.03,331.59) | 0.74(0.58,0.90) |
| Burundi | 10133.03(7746.53,13028.39) | 501.17(383.13,644.37) |  | 23023.99(17507.59,31897.65) | 447.48(340.27,619.95) |  | 127.22(65.72,202.29) | -1.04(-1.30,-0.79) |
| Cabo Verde | 268.60(212.18,343.64) | 224.08(177.01,286.69) |  | 1781.14(1361.85,2330.05) | 614.46(469.81,803.83) |  | 563.11(474.92,661.94) | 3.50(3.17,3.83) |
| Cambodia | 13549.16(10687.53,17128.62) | 352.98(278.43,446.23) |  | 41463.43(31468.23,53106.05) | 503.25(381.94,644.56) |  | 206.02(136.47,298.27) | 1.15(1.06,1.24) |
| Cameroon | 15154.69(12110.17,19076.50) | 403.12(322.13,507.44) |  | 81890.40(61842.22,106265.29) | 637.71(481.59,827.52) |  | 440.36(322.11,587.57) | 1.28(1.06,1.49) |
| Canada | 13812.31(11696.92,16400.53) | 97.76(82.79,116.08) |  | 52596.60(37384.10,71726.32) | 309.95(220.31,422.69) |  | 280.80(212.70,354.96) | 3.19(2.87,3.51) |
| Central African Republic | 8244.01(6600.59,9945.02) | 789.14(631.83,951.96) |  | 24450.38(18390.47,31349.71) | 1095.24(823.79,1404.29) |  | 196.58(131.23,283.19) | 1.14(1.09,1.18) |
| Chad | 5726.94(4615.07,7100.52) | 281.04(226.48,348.44) |  | 29477.43(22971.06,37886.17) | 502.06(391.25,645.28) |  | 414.71(312.78,519.21) | 1.81(1.54,2.07) |
| Chile | 12340.39(9892.26,15054.47) | 194.97(156.29,237.85) |  | 33014.95(23540.29,45206.37) | 350.24(249.73,479.57) |  | 167.54(132.87,206.29) | 2.14(1.95,2.33) |
| China | 1468055.27(1095824.69,1928795.08) | 249.71(186.40,328.08) |  | 3883238.14(2742745.88,5256087.98) | 547.33(386.58,740.83) |  | 164.52(141.23,187.35) | 2.63(2.33,2.93) |
| Colombia | 60203.10(47992.19,76537.84) | 413.49(329.62,525.68) |  | 139750.22(101428.10,190070.43) | 560.40(406.73,762.19) |  | 132.13(107.17,156.17) | 0.58(0.45,0.72) |
| Comoros | 775.31(543.73,1008.51) | 467.63(327.95,608.28) |  | 2195.84(1688.09,2762.47) | 628.11(482.87,790.19) |  | 183.22(107.43,307.27) | 0.83(0.54,1.12) |
| Congo | 6736.26(5221.75,8390.45) | 750.91(582.08,935.30) |  | 23956.10(18444.75,31176.61) | 969.79(746.68,1262.09) |  | 255.63(162.17,373.70) | 0.65(0.41,0.90) |
| Cook Islands | 164.81(134.42,200.28) | 2015.15(1643.58,2448.79) |  | 249.69(196.57,320.41) | 3180.01(2503.55,4080.80) |  | 51.50(18.56,93.26) | 1.64(1.52,1.75) |
| Costa Rica | 4612.68(3482.46,5999.43) | 344.45(260.05,448.00) |  | 17311.52(12691.83,23651.16) | 714.92(524.14,976.73) |  | 275.30(241.34,305.72) | 2.27(2.20,2.35) |
| Croatia | 5922.72(4514.22,7776.51) | 244.56(186.40,321.11) |  | 7079.96(5078.37,9854.83) | 370.37(265.66,515.53) |  | 19.54(6.99,33.85) | 1.48(1.33,1.63) |
| Cuba | 22606.26(18096.86,28511.90) | 411.00(329.01,518.37) |  | 36478.40(25484.19,50213.28) | 669.17(467.49,921.13) |  | 61.36(39.48,82.05) | 1.59(1.38,1.79) |
| Cyprus | 991.56(779.41,1221.07) | 261.42(205.49,321.93) |  | 2368.88(1728.50,3189.49) | 327.56(239.01,441.03) |  | 138.91(94.13,183.92) | 0.38(0.26,0.51) |
| Czechia | 12060.58(9024.44,16048.06) | 247.01(184.83,328.67) |  | 20855.54(14823.42,29631.84) | 424.03(301.39,602.47) |  | 72.92(51.69,94.70) | 1.68(1.50,1.86) |
| C么te d'Ivoire | 15845.70(12765.78,19600.99) | 341.60(275.20,422.55) |  | 70336.80(53469.33,89956.22) | 601.99(457.63,769.91) |  | 343.89(253.77,448.65) | 1.71(1.57,1.86) |
| Democratic People's Republic of Korea | 31557.75(23706.95,40053.48) | 311.20(233.78,394.98) |  | 80786.38(59981.43,106902.37) | 570.76(423.77,755.27) |  | 156.00(109.27,205.45) | 2.11(2.00,2.23) |
| Democratic Republic of the Congo | 69200.77(54258.25,86489.04) | 499.40(391.57,624.17) |  | 239912.36(189557.34,305195.04) | 665.11(525.51,846.10) |  | 246.69(163.40,341.11) | 0.96(0.86,1.06) |
| Denmark | 4189.43(3397.95,5096.49) | 161.83(131.25,196.87) |  | 6684.03(4698.32,8998.96) | 253.92(178.48,341.86) |  | 59.55(35.66,81.19) | 1.55(1.37,1.73) |
| Djibouti | 525.34(391.99,705.01) | 311.74(232.61,418.36) |  | 3249.15(2431.76,4367.69) | 518.53(388.08,697.03) |  | 518.49(346.95,772.17) | 1.55(1.38,1.72) |
| Dominica | 225.89(190.63,273.34) | 757.20(639.02,916.25) |  | 477.58(373.52,629.99) | 1466.40(1146.88,1934.37) |  | 111.42(79.07,143.21) | 2.30(2.21,2.38) |
| Dominican Republic | 12976.02(10504.75,16537.80) | 427.22(345.86,544.49) |  | 54747.43(41145.07,71047.00) | 1004.32(754.79,1303.33) |  | 321.91(261.00,384.25) | 3.16(3.05,3.28) |
| Ecuador | 13633.90(11605.92,16147.68) | 327.67(278.93,388.09) |  | 53234.84(41348.05,67009.82) | 613.19(476.27,771.86) |  | 290.46(235.66,344.87) | 1.87(1.56,2.19) |
| Egypt | 76592.18(66352.68,87819.12) | 333.89(289.25,382.83) |  | 343221.25(265263.70,443473.02) | 714.88(552.50,923.68) |  | 348.12(267.13,437.10) | 2.62(2.55,2.69) |
| El Salvador | 9182.63(7675.62,10949.64) | 445.47(372.36,531.19) |  | 29962.73(24443.58,37370.52) | 983.81(802.60,1227.05) |  | 226.30(180.41,281.42) | 2.58(2.45,2.72) |
| Equatorial Guinea | 1086.80(841.29,1416.62) | 720.21(557.51,938.77) |  | 5088.62(3799.36,6792.84) | 767.42(572.98,1024.43) |  | 368.22(235.52,551.56) | 0.24(-0.19,0.67) |
| Eritrea | 7536.96(5870.97,9696.59) | 597.02(465.05,768.09) |  | 20355.45(15017.89,26813.04) | 692.21(510.70,911.81) |  | 170.07(101.99,251.83) | 0.51(0.42,0.60) |
| Estonia | 1284.19(932.79,1705.03) | 171.07(124.26,227.12) |  | 2472.01(1784.18,3377.83) | 416.83(300.84,569.56) |  | 92.50(74.80,112.46) | 2.47(2.25,2.68) |
| Eswatini | 1373.99(1072.17,1724.13) | 483.61(377.38,606.85) |  | 4946.61(3497.41,6626.37) | 933.87(660.27,1250.99) |  | 260.02(155.62,401.08) | 2.40(1.57,3.24) |
| Ethiopia | 136480.14(114435.06,166262.23) | 762.95(639.72,929.44) |  | 184367.56(149974.49,223406.59) | 410.67(334.06,497.63) |  | 35.09(6.02,68.85) | -2.58(-2.86,-2.29) |
| Fiji | 8433.24(7145.11,9933.80) | 2456.48(2081.26,2893.57) |  | 17761.71(13981.08,22569.14) | 4063.50(3198.57,5163.33) |  | 110.62(58.16,174.43) | 1.63(1.47,1.79) |
| Finland | 5331.37(3739.62,7162.56) | 208.76(146.43,280.46) |  | 9830.14(6482.39,13660.66) | 413.95(272.97,575.25) |  | 84.38(65.69,103.42) | 2.00(1.92,2.09) |
| France | 29965.11(23195.30,38209.64) | 108.13(83.70,137.88) |  | 64751.80(45090.17,89088.54) | 226.98(158.06,312.28) |  | 116.09(87.93,143.59) | 2.67(2.38,2.96) |
| Gabon | 2672.92(2096.10,3463.77) | 708.14(555.33,917.67) |  | 8440.08(6386.58,10925.14) | 1041.62(788.19,1348.31) |  | 215.76(140.84,322.21) | 1.28(1.13,1.43) |
| Gambia | 1048.79(814.52,1340.23) | 287.79(223.50,367.76) |  | 5353.78(4148.83,6927.48) | 550.29(426.44,712.05) |  | 410.47(298.08,537.48) | 2.00(1.84,2.16) |
| Georgia | 6479.20(5090.51,8350.82) | 247.73(194.63,319.29) |  | 9932.27(7435.98,13102.65) | 610.04(456.72,804.77) |  | 53.29(36.34,69.93) | 3.54(3.20,3.89) |
| Germany | 66811.81(53477.39,81938.52) | 160.50(128.47,196.84) |  | 121618.60(85473.23,161652.13) | 320.80(225.46,426.40) |  | 82.03(57.09,102.94) | 2.56(2.38,2.74) |
| Ghana | 20618.46(16576.28,25279.96) | 362.32(291.29,444.23) |  | 100216.21(79406.12,128418.44) | 659.11(522.25,844.59) |  | 386.05(282.86,512.34) | 2.25(1.98,2.52) |
| Greece | 10280.13(7268.04,13997.05) | 209.58(148.17,285.36) |  | 21041.88(14135.41,29440.37) | 458.01(307.68,640.82) |  | 104.68(88.36,121.61) | 2.46(2.40,2.53) |
| Greenland | 48.53(40.15,59.30) | 150.92(124.87,184.41) |  | 75.75(57.25,97.20) | 278.96(210.84,357.94) |  | 56.09(15.47,105.74) | 2.12(2.03,2.20) |
| Grenada | 349.98(304.25,404.97) | 1067.23(927.77,1234.92) |  | 840.17(680.97,1068.53) | 1602.32(1298.71,2037.84) |  | 140.06(106.88,177.05) | 1.42(1.29,1.54) |
| Guam | 384.26(315.12,470.90) | 550.76(451.66,674.93) |  | 814.14(626.85,1031.98) | 1114.86(858.40,1413.17) |  | 111.87(87.60,137.15) | 2.40(2.19,2.61) |
| Guatemala | 12175.72(9977.47,14805.23) | 420.08(344.24,510.80) |  | 101005.73(84842.46,123809.52) | 1379.55(1158.79,1691.00) |  | 729.57(636.95,831.72) | 3.29(2.98,3.61) |
| Guinea | 7262.04(5649.05,9137.90) | 337.23(262.32,424.34) |  | 25613.00(19679.80,32339.43) | 507.01(389.56,640.16) |  | 252.70(174.63,352.82) | 1.31(1.16,1.46) |
| Guinea-Bissau | 1880.42(1476.58,2349.26) | 529.43(415.73,661.43) |  | 6110.59(4748.35,7901.01) | 735.76(571.74,951.34) |  | 224.96(151.05,320.50) | 0.94(0.81,1.07) |
| Guyana | 4014.85(3411.99,4853.73) | 1192.13(1013.12,1441.22) |  | 7941.30(6094.76,10140.57) | 2138.15(1640.98,2730.29) |  | 97.80(65.39,135.59) | 2.08(1.67,2.50) |
| Haiti | 27681.82(22300.31,34963.02) | 1109.48(893.79,1401.30) |  | 79861.43(60222.98,103894.61) | 1315.42(991.95,1711.27) |  | 188.50(124.45,261.07) | 0.59(0.48,0.69) |
| Honduras | 7224.91(5713.73,9263.82) | 434.40(343.54,556.99) |  | 35557.02(26196.43,47829.70) | 755.25(556.42,1015.92) |  | 392.15(321.19,466.88) | 1.72(1.64,1.81) |
| Hungary | 15049.46(11659.78,19351.09) | 306.07(237.13,393.55) |  | 20810.48(14975.71,28254.58) | 458.20(329.73,622.10) |  | 38.28(21.46,55.37) | 1.19(0.94,1.43) |
| Iceland | 155.11(108.56,211.88) | 127.49(89.23,174.15) |  | 496.47(326.31,697.91) | 302.92(199.10,425.83) |  | 220.07(192.90,250.79) | 2.79(2.70,2.88) |
| India | 1251350.17(1041641.20,1495387.70) | 341.92(284.62,408.60) |  | 4186480.03(3263243.97,5280941.77) | 587.27(457.76,740.79) |  | 234.56(201.79,264.17) | 1.71(1.53,1.89) |
| Indonesia | 264314.87(222349.40,307770.84) | 324.85(273.28,378.26) |  | 812544.13(659097.10,969658.83) | 553.44(448.92,660.45) |  | 207.42(164.63,255.94) | 1.17(0.88,1.45) |
| Iran (Islamic Republic of) | 36994.79(29376.77,46246.74) | 176.33(140.02,220.43) |  | 228118.33(168850.78,298762.52) | 490.47(363.04,642.36) |  | 516.62(449.30,568.27) | 3.28(3.11,3.45) |
| Iraq | 40304.55(31778.24,50097.35) | 587.82(463.47,730.65) |  | 225972.14(168397.71,300483.12) | 1146.94(854.71,1525.12) |  | 460.66(362.18,565.31) | 2.22(2.09,2.36) |
| Ireland | 2413.37(1749.05,3205.61) | 151.54(109.82,201.28) |  | 5782.12(3855.56,8101.05) | 250.59(167.09,351.09) |  | 139.59(114.50,163.21) | 1.66(1.53,1.78) |
| Israel | 4994.38(3964.55,6304.28) | 232.92(184.90,294.01) |  | 13552.78(9869.68,17871.48) | 321.01(233.77,423.30) |  | 171.36(144.05,201.20) | 0.90(0.60,1.21) |
| Italy | 61804.92(47597.21,78840.72) | 221.05(170.23,281.98) |  | 78103.43(55531.17,106951.51) | 293.46(208.65,401.85) |  | 26.37(14.60,37.39) | 1.14(0.93,1.35) |
| Jamaica | 5701.19(4954.12,6581.60) | 582.65(506.30,672.63) |  | 13816.99(10712.79,17639.70) | 949.21(735.95,1211.82) |  | 142.35(101.74,186.54) | 1.28(0.98,1.58) |
| Japan | 164546.78(124191.17,213729.44) | 261.80(197.59,340.05) |  | 238397.83(166162.23,329648.87) | 439.34(306.21,607.50) |  | 44.88(32.31,57.12) | 1.50(1.36,1.64) |
| Jordan | 6913.70(5635.84,8616.52) | 485.49(395.75,605.06) |  | 51693.02(37846.72,68496.92) | 841.60(616.17,1115.18) |  | 647.69(504.99,775.78) | 1.97(1.85,2.09) |
| Kazakhstan | 17833.99(13268.21,23533.50) | 232.87(173.25,307.29) |  | 46007.83(32244.83,62844.30) | 507.92(355.98,693.80) |  | 157.98(129.90,183.90) | 2.24(2.02,2.46) |
| Kenya | 17355.56(14665.66,20702.33) | 216.33(182.80,258.05) |  | 76071.07(63579.57,91742.41) | 350.22(292.71,422.36) |  | 338.31(267.37,421.63) | 1.67(1.45,1.88) |
| Kiribati | 662.95(543.29,793.65) | 2085.05(1708.69,2496.10) |  | 1801.45(1380.51,2315.09) | 3267.11(2503.68,4198.64) |  | 171.73(105.24,274.93) | 1.35(1.08,1.63) |
| Kuwait | 3427.42(2526.08,4511.77) | 364.17(268.40,479.39) |  | 30251.97(20244.22,42184.02) | 979.54(655.49,1365.89) |  | 782.65(691.54,868.55) | 3.23(3.07,3.39) |
| Kyrgyzstan | 2922.50(2238.75,3838.96) | 159.04(121.83,208.91) |  | 10879.25(7958.71,14651.70) | 340.56(249.14,458.65) |  | 272.26(226.80,319.12) | 2.48(2.30,2.66) |
| Lao People's Democratic Republic | 8159.07(6287.69,10435.82) | 523.66(403.55,669.79) |  | 21429.99(16460.01,26537.35) | 593.15(455.59,734.52) |  | 162.65(95.11,253.46) | 0.19(0.11,0.27) |
| Latvia | 2573.98(1998.41,3330.90) | 201.02(156.07,260.14) |  | 4240.82(3324.11,5532.77) | 513.90(402.81,670.46) |  | 64.76(51.37,79.43) | 2.58(2.20,2.97) |
| Lebanon | 7173.80(5692.65,9105.25) | 563.22(446.93,714.86) |  | 23752.83(16973.71,32127.78) | 820.72(586.48,1110.09) |  | 231.11(164.72,295.38) | 1.37(1.22,1.53) |
| Lesotho | 1586.83(1224.38,2043.96) | 285.70(220.45,368.01) |  | 6054.48(4448.09,8030.91) | 708.12(520.24,939.28) |  | 281.55(172.15,429.13) | 3.50(2.81,4.21) |
| Liberia | 3042.64(2451.51,3759.35) | 335.83(270.58,414.93) |  | 15467.96(11516.43,20377.24) | 661.22(492.30,871.07) |  | 408.37(303.96,557.47) | 1.79(1.61,1.96) |
| Libya | 3895.74(2944.17,5006.70) | 244.14(184.51,313.76) |  | 32476.24(23711.34,43802.05) | 822.87(600.79,1109.83) |  | 733.63(622.58,843.43) | 4.47(4.29,4.66) |
| Lithuania | 2546.13(1868.04,3378.16) | 143.50(105.29,190.40) |  | 4876.53(3754.32,6517.87) | 398.17(306.54,532.19) |  | 91.53(74.47,111.80) | 3.15(2.94,3.36) |
| Luxembourg | 347.56(259.66,456.15) | 174.16(130.12,228.58) |  | 1034.63(692.79,1436.29) | 311.97(208.89,433.08) |  | 197.69(163.09,227.90) | 1.95(1.90,2.01) |
| Madagascar | 15526.57(12742.61,18605.89) | 358.61(294.31,429.73) |  | 50814.62(39060.49,64782.55) | 431.84(331.95,550.55) |  | 227.28(151.89,314.32) | 0.70(0.61,0.78) |
| Malawi | 13188.18(11030.26,16100.90) | 365.99(306.10,446.82) |  | 34871.52(28052.18,43163.30) | 453.59(364.89,561.45) |  | 164.41(102.57,247.81) | 0.28(-0.01,0.57) |
| Malaysia | 28976.44(24083.67,35624.73) | 369.22(306.87,453.93) |  | 85743.28(67168.92,108661.52) | 514.84(403.31,652.46) |  | 195.91(157.78,234.76) | 1.00(0.87,1.12) |
| Maldives | 343.96(278.82,421.04) | 437.69(354.80,535.78) |  | 1147.90(857.11,1524.74) | 345.23(257.77,458.56) |  | 233.74(159.84,331.58) | -0.97(-1.42,-0.52) |
| Mali | 15198.20(12161.59,18916.77) | 498.58(398.96,620.57) |  | 62315.89(47570.62,81435.69) | 739.77(564.72,966.75) |  | 310.02(233.64,384.92) | 1.42(1.27,1.56) |
| Malta | 341.23(271.92,434.35) | 186.58(148.68,237.49) |  | 894.54(626.36,1213.02) | 446.25(312.46,605.12) |  | 162.15(127.94,195.39) | 2.65(2.53,2.78) |
| Marshall Islands | 249.66(201.47,302.83) | 1551.16(1251.77,1881.48) |  | 1110.87(811.74,1477.77) | 4062.85(2968.85,5404.76) |  | 344.95(250.11,448.56) | 3.03(2.72,3.35) |
| Mauritania | 2259.34(1818.63,2754.98) | 299.38(240.98,365.05) |  | 6630.30(5180.68,8435.68) | 388.80(303.80,494.67) |  | 193.46(129.88,266.96) | 0.63(0.57,0.70) |
| Mauritius | 4277.17(3808.24,4842.45) | 786.97(700.69,890.98) |  | 13317.66(11722.99,15546.42) | 2091.58(1841.13,2441.61) |  | 211.37(185.76,236.98) | 4.48(3.90,5.07) |
| Mexico | 358893.75(307793.38,413753.00) | 1023.33(877.63,1179.75) |  | 978151.54(820235.67,1163672.11) | 1514.76(1270.21,1802.06) |  | 172.55(152.61,194.79) | 1.21(1.02,1.40) |
| Micronesia (Federated States of) | 483.72(373.31,633.21) | 1272.20(981.83,1665.36) |  | 1246.15(952.01,1591.23) | 2597.53(1984.41,3316.85) |  | 157.62(98.05,231.14) | 2.43(2.09,2.78) |
| Monaco | 21.00(15.00,28.68) | 144.99(103.56,198.01) |  | 51.10(33.96,70.98) | 330.81(219.82,459.49) |  | 143.31(118.83,167.84) | 2.74(2.71,2.78) |
| Mongolia | 1111.47(841.41,1435.59) | 130.85(99.06,169.01) |  | 6636.95(5016.67,8692.67) | 407.48(308.00,533.69) |  | 497.13(411.64,594.30) | 4.07(3.93,4.22) |
| Montenegro | 759.59(568.82,1000.92) | 250.91(187.90,330.63) |  | 1264.45(912.96,1743.86) | 431.12(311.28,594.57) |  | 66.47(43.96,88.70) | 1.97(1.72,2.23) |
| Morocco | 29718.98(21755.46,39270.03) | 282.17(206.56,372.85) |  | 177468.15(124922.85,239603.82) | 972.27(684.40,1312.68) |  | 497.15(425.10,562.92) | 4.32(4.21,4.44) |
| Mozambique | 20262.44(16435.32,24687.42) | 421.08(341.54,513.03) |  | 74308.56(56991.73,96401.30) | 644.52(494.32,836.14) |  | 266.73(171.61,392.29) | 2.09(1.82,2.36) |
| Myanmar | 145333.78(111475.92,183900.93) | 834.64(640.20,1056.13) |  | 275074.81(221047.51,347910.62) | 1010.96(812.39,1278.64) |  | 89.27(38.59,158.23) | 0.42(0.33,0.52) |
| Namibia | 1805.90(1467.90,2214.54) | 338.27(274.96,414.81) |  | 5880.61(4356.51,7743.21) | 518.65(384.23,682.92) |  | 225.63(145.42,328.89) | 1.07(0.72,1.43) |
| Nauru | 82.78(65.22,106.47) | 1937.59(1526.56,2492.27) |  | 146.13(112.23,185.81) | 2939.72(2257.81,3737.93) |  | 76.54(37.42,135.77) | 1.10(0.88,1.33) |
| Nepal | 27351.27(21422.20,34915.43) | 360.79(282.58,460.56) |  | 99227.97(74727.52,132093.40) | 674.22(507.75,897.53) |  | 262.79(210.87,324.36) | 2.10(1.80,2.39) |
| Netherlands | 13009.43(10285.66,16286.21) | 167.26(132.24,209.39) |  | 21853.09(14919.51,30323.38) | 284.96(194.55,395.42) |  | 67.98(40.90,95.19) | 1.66(1.52,1.81) |
| New Zealand | 3512.91(2833.71,4346.39) | 211.85(170.89,262.11) |  | 7644.37(5878.61,9770.19) | 313.34(240.96,400.48) |  | 117.61(95.22,142.00) | 1.41(1.24,1.57) |
| Nicaragua | 6359.84(5164.94,7856.44) | 457.94(371.90,565.70) |  | 26261.05(20300.89,33598.95) | 806.62(623.55,1032.01) |  | 312.92(263.82,358.57) | 1.78(1.71,1.86) |
| Niger | 7579.02(6012.61,9544.63) | 276.49(219.35,348.20) |  | 32154.93(24488.01,42239.70) | 398.98(303.85,524.12) |  | 324.26(253.80,406.71) | 1.27(1.21,1.34) |
| Nigeria | 116618.17(94612.19,144841.48) | 340.61(276.34,423.05) |  | 352184.51(278422.50,450789.47) | 398.44(314.99,509.99) |  | 202.00(136.07,285.65) | 0.42(0.34,0.49) |
| Niue | 14.58(11.24,18.48) | 1583.08(1220.29,2006.81) |  | 24.08(18.57,31.24) | 3200.24(2468.22,4151.80) |  | 65.14(26.81,116.93) | 2.21(2.04,2.37) |
| North Macedonia | 2751.33(2175.38,3512.55) | 284.70(225.10,363.46) |  | 5719.53(4159.09,7842.15) | 503.05(365.80,689.74) |  | 107.88(76.23,137.60) | 1.84(1.57,2.11) |
| Northern Mariana Islands | 228.84(178.70,292.24) | 864.68(675.24,1104.24) |  | 414.27(343.37,509.92) | 1742.48(1444.23,2144.76) |  | 81.04(43.56,129.38) | 3.06(2.57,3.56) |
| Norway | 4479.19(3238.65,5946.28) | 218.43(157.93,289.97) |  | 7595.90(5278.31,10417.30) | 297.31(206.60,407.74) |  | 69.58(60.80,77.73) | 0.58(0.38,0.79) |
| Oman | 2711.05(2040.53,3529.80) | 307.51(231.46,400.38) |  | 15962.32(12152.78,20513.29) | 550.51(419.12,707.46) |  | 488.79(343.92,636.21) | 1.06(0.85,1.27) |
| Pakistan | 158292.62(127076.01,193080.02) | 385.66(309.61,470.42) |  | 784995.09(609125.71,985062.81) | 737.06(571.93,924.91) |  | 395.91(327.15,458.53) | 2.03(1.85,2.20) |
| Palau | 98.35(77.18,122.18) | 1324.29(1039.24,1645.14) |  | 316.99(254.30,394.97) | 3343.28(2682.16,4165.74) |  | 222.31(155.10,316.12) | 3.09(2.92,3.26) |
| Palestine | 2402.98(1867.66,3138.42) | 339.31(263.72,443.16) |  | 13382.43(10158.11,17018.63) | 582.48(442.14,740.75) |  | 456.91(346.61,583.10) | 1.87(1.80,1.95) |
| Panama | 4108.55(3317.60,5198.98) | 387.73(313.09,490.64) |  | 15706.62(11996.34,20284.74) | 769.48(587.71,993.76) |  | 282.29(240.79,325.38) | 2.33(2.19,2.47) |
| Papua New Guinea | 22062.65(16100.89,28460.02) | 1314.31(959.16,1695.42) |  | 85303.80(67363.04,107767.23) | 1799.20(1420.80,2272.99) |  | 286.64(178.87,439.80) | 1.01(0.93,1.09) |
| Paraguay | 6023.98(5103.51,7420.80) | 373.98(316.83,460.69) |  | 26229.33(20614.89,32924.39) | 747.69(587.64,938.53) |  | 335.42(268.72,419.50) | 2.39(2.12,2.66) |
| Peru | 18825.47(15460.41,22742.59) | 208.88(171.54,252.34) |  | 62507.66(48257.33,80985.09) | 344.08(265.63,445.79) |  | 232.04(172.85,297.27) | 1.68(1.45,1.91) |
| Philippines | 117532.85(103409.47,133842.69) | 448.91(394.97,511.20) |  | 353355.58(300929.12,413541.76) | 648.04(551.89,758.42) |  | 200.64(163.09,241.59) | 1.24(1.10,1.37) |
| Poland | 53711.82(42845.39,66772.42) | 298.12(237.80,370.61) |  | 81307.39(61262.91,105861.84) | 441.30(332.51,574.57) |  | 51.38(40.37,60.71) | 1.18(1.06,1.31) |
| Portugal | 14314.62(11381.03,17848.32) | 303.39(241.22,378.29) |  | 25222.52(17484.67,34342.15) | 525.42(364.23,715.39) |  | 76.20(51.78,98.24) | 1.80(1.72,1.88) |
| Puerto Rico | 13086.80(10944.51,15945.25) | 782.01(654.00,952.82) |  | 20702.63(16043.63,26968.08) | 1405.00(1088.81,1830.21) |  | 58.19(41.14,76.32) | 1.68(1.51,1.86) |
| Qatar | 1042.06(832.62,1313.17) | 378.35(302.30,476.78) |  | 16741.68(12135.09,22760.58) | 751.83(544.96,1022.13) |  | 1506.59(1160.32,1855.78) | 1.45(1.04,1.86) |
| Republic of Korea | 95401.35(78355.62,116792.30) | 408.72(335.69,500.36) |  | 221021.77(152126.44,302950.62) | 835.20(574.85,1144.79) |  | 131.68(92.45,167.82) | 2.49(2.26,2.72) |
| Republic of Moldova | 4615.75(3486.87,6062.02) | 220.42(166.51,289.49) |  | 8912.66(6481.44,11892.74) | 484.02(351.99,645.86) |  | 93.09(76.09,109.37) | 2.20(1.96,2.43) |
| Romania | 23348.93(17951.06,30908.67) | 214.74(165.10,284.27) |  | 30630.08(21574.48,42671.22) | 344.45(242.62,479.87) |  | 31.18(15.70,47.04) | 1.40(1.21,1.60) |
| Russian Federation | 112243.30(84447.47,144767.51) | 151.19(113.75,195.01) |  | 242844.63(184946.79,312603.50) | 354.21(269.76,455.96) |  | 116.36(107.36,126.43) | 2.41(2.21,2.62) |
| Rwanda | 14718.20(10619.65,19954.27) | 569.86(411.17,772.59) |  | 22542.87(14990.55,32114.02) | 390.39(259.60,556.14) |  | 53.16(11.08,112.56) | -2.52(-3.02,-2.01) |
| Saint Kitts and Nevis | 130.59(112.53,153.42) | 781.64(673.54,918.29) |  | 371.07(283.79,488.56) | 1170.44(895.14,1541.00) |  | 184.14(140.57,231.62) | 1.31(1.20,1.43) |
| Saint Lucia | 596.02(514.79,705.01) | 1080.47(933.21,1278.04) |  | 1492.24(1157.21,1945.31) | 1596.46(1238.03,2081.16) |  | 150.37(116.56,185.40) | 1.35(1.23,1.47) |
| Saint Vincent and the Grenadines | 459.25(403.66,526.50) | 1050.08(922.97,1203.85) |  | 999.80(810.27,1243.46) | 1819.62(1474.69,2263.08) |  | 117.70(90.36,147.52) | 1.85(1.72,1.99) |
| Samoa | 690.49(541.95,858.81) | 1097.33(861.27,1364.81) |  | 1968.09(1532.12,2502.48) | 2234.52(1739.54,2841.26) |  | 185.03(123.92,254.41) | 2.61(2.46,2.76) |
| San Marino | 17.21(12.52,23.16) | 144.68(105.20,194.68) |  | 48.46(32.30,68.32) | 330.23(220.09,465.59) |  | 181.52(151.87,210.22) | 2.94(2.87,3.02) |
| Sao Tome and Principe | 88.90(67.38,116.08) | 221.14(167.60,288.76) |  | 448.80(330.94,605.42) | 464.93(342.84,627.18) |  | 404.86(327.78,480.60) | 2.57(2.50,2.64) |
| Saudi Arabia | 22643.10(17235.06,28759.06) | 335.25(255.18,425.80) |  | 209758.66(156817.49,273413.74) | 854.06(638.50,1113.24) |  | 826.37(607.75,1035.30) | 2.96(2.86,3.05) |
| Senegal | 10746.16(8615.41,13481.01) | 404.45(324.25,507.38) |  | 41237.65(31882.70,52698.88) | 639.04(494.07,816.65) |  | 283.74(214.84,358.10) | 1.71(1.55,1.86) |
| Serbia | 15228.07(11552.69,19910.28) | 327.58(248.51,428.30) |  | 19819.26(14366.01,27439.38) | 467.39(338.79,647.09) |  | 30.15(14.31,48.45) | 1.12(0.91,1.32) |
| Seychelles | 88.43(71.79,108.82) | 276.05(224.09,339.70) |  | 496.43(369.07,650.26) | 916.59(681.44,1200.61) |  | 461.36(379.48,533.89) | 4.29(4.15,4.44) |
| Sierra Leone | 4395.25(3481.16,5535.59) | 272.24(215.62,342.87) |  | 18234.40(14059.68,23815.81) | 491.16(378.71,641.50) |  | 314.87(233.00,402.53) | 1.94(1.78,2.10) |
| Singapore | 6843.05(5124.90,8946.62) | 388.00(290.58,507.27) |  | 19406.01(12699.65,28133.81) | 613.13(401.25,888.89) |  | 183.59(143.56,223.28) | 1.25(1.08,1.41) |
| Slovakia | 5291.55(4057.03,6888.74) | 212.46(162.89,276.59) |  | 8547.08(6033.23,11784.67) | 320.35(226.13,441.69) |  | 61.52(39.77,81.61) | 1.20(1.04,1.37) |
| Slovenia | 2105.27(1613.45,2761.61) | 213.51(163.63,280.07) |  | 3093.22(2209.49,4274.86) | 331.66(236.91,458.36) |  | 46.93(30.07,63.09) | 1.22(1.06,1.37) |
| Solomon Islands | 1610.18(993.89,2334.81) | 1321.60(815.76,1916.35) |  | 7253.45(5641.33,9215.38) | 2429.35(1889.41,3086.44) |  | 350.47(207.89,644.41) | 2.15(2.02,2.29) |
| Somalia | 15479.35(11532.51,21201.45) | 543.84(405.17,744.88) |  | 44061.16(33026.96,56243.64) | 559.22(419.17,713.84) |  | 184.64(112.73,292.69) | -0.49(-0.66,-0.31) |
| South Africa | 82215.87(73337.07,92397.72) | 513.34(457.90,576.91) |  | 223052.51(191774.51,254724.11) | 767.88(660.20,876.91) |  | 171.30(143.42,197.80) | 1.61(1.36,1.86) |
| South Sudan | 8265.44(6099.36,11207.84) | 379.73(280.22,514.91) |  | 23174.27(17453.51,30045.04) | 650.51(489.93,843.38) |  | 180.38(104.29,284.28) | 1.70(1.26,2.15) |
| Spain | 43776.60(32627.60,58388.19) | 242.20(180.52,323.04) |  | 98840.77(65884.65,137481.48) | 464.76(309.80,646.46) |  | 125.78(97.36,150.31) | 2.02(1.78,2.26) |
| Sri Lanka | 34325.71(28664.21,40838.93) | 423.22(353.41,503.52) |  | 104022.42(78123.48,140414.56) | 985.64(740.24,1330.46) |  | 203.05(137.39,281.64) | 3.10(2.77,3.44) |
| Sudan | 16062.20(12319.12,20441.29) | 214.50(164.51,272.98) |  | 85879.65(63238.66,113048.25) | 453.67(334.06,597.19) |  | 434.67(350.83,527.88) | 2.58(2.48,2.68) |
| Suriname | 1239.13(1018.65,1508.37) | 719.56(591.52,875.90) |  | 4292.67(3379.04,5619.38) | 1545.84(1216.83,2023.61) |  | 246.43(197.81,306.36) | 2.79(2.59,2.99) |
| Sweden | 8838.70(6674.71,11373.99) | 215.23(162.53,276.97) |  | 13941.91(9748.25,19017.29) | 303.63(212.30,414.16) |  | 57.74(41.66,71.83) | 0.92(0.83,1.01) |
| Switzerland | 8741.22(6264.22,11513.55) | 242.81(174.00,319.81) |  | 18851.83(12454.12,26343.16) | 445.73(294.47,622.86) |  | 115.67(91.07,137.04) | 1.89(1.78,2.00) |
| Syrian Arab Republic | 12955.63(10214.71,16376.17) | 288.93(227.81,365.22) |  | 43098.25(31457.09,57488.53) | 692.05(505.12,923.12) |  | 232.66(177.63,290.51) | 2.80(2.27,3.33) |
| Taiwan (Province of China) | 36631.06(30081.78,44976.36) | 355.36(291.82,436.31) |  | 72127.00(53656.22,95463.09) | 598.00(444.86,791.48) |  | 96.90(73.38,118.27) | 1.21(0.88,1.54) |
| Tajikistan | 4440.05(3576.68,5556.75) | 219.13(176.52,274.24) |  | 17516.49(13148.29,22764.63) | 374.47(281.09,486.67) |  | 294.51(227.84,383.27) | 1.54(1.41,1.67) |
| Thailand | 92372.29(75634.45,111894.94) | 328.92(269.32,398.44) |  | 235517.97(183737.14,301821.23) | 701.01(546.88,898.36) |  | 154.97(104.71,224.41) | 2.15(1.85,2.44) |
| Timor-Leste | 741.32(569.79,943.11) | 223.95(172.13,284.91) |  | 2304.08(1768.00,3001.29) | 404.03(310.03,526.29) |  | 210.81(133.08,303.31) | 1.96(1.74,2.17) |
| Togo | 3347.51(2733.23,4194.72) | 257.72(210.43,322.95) |  | 16624.49(12695.67,21428.09) | 465.56(355.54,600.09) |  | 396.62(289.99,534.12) | 1.92(1.79,2.05) |
| Tokelau | 8.46(6.35,11.35) | 1410.38(1059.36,1892.33) |  | 15.20(11.74,19.85) | 2505.15(1934.46,3271.97) |  | 79.73(42.66,125.84) | 1.89(1.71,2.08) |
| Tonga | 485.02(402.68,579.00) | 1347.29(1118.56,1608.35) |  | 958.97(743.43,1235.25) | 2215.41(1717.47,2853.66) |  | 97.72(54.47,150.52) | 1.67(1.62,1.72) |
| Trinidad and Tobago | 8989.05(8069.17,10194.50) | 1640.25(1472.40,1860.21) |  | 14390.74(11422.83,18397.43) | 2113.76(1677.82,2702.27) |  | 60.09(33.12,89.96) | 0.39(0.23,0.56) |
| Tunisia | 7090.81(5238.26,9422.22) | 203.25(150.15,270.08) |  | 40054.96(27984.42,55628.68) | 676.62(472.72,939.69) |  | 464.89(398.07,537.72) | 4.05(3.92,4.17) |
| Turkey | 74677.88(61102.64,92136.57) | 300.92(246.22,371.27) |  | 218500.19(159996.49,294833.49) | 515.59(377.54,695.71) |  | 192.59(132.68,255.82) | 2.00(1.62,2.38) |
| Turkmenistan | 2583.20(2094.10,3126.13) | 173.46(140.62,209.91) |  | 13852.26(11057.79,17336.60) | 556.29(444.07,696.22) |  | 436.24(354.72,537.79) | 3.95(3.70,4.19) |
| Tuvalu | 56.97(45.00,72.67) | 1397.32(1103.71,1782.45) |  | 112.37(88.63,141.33) | 1995.17(1573.72,2509.35) |  | 97.23(57.12,139.48) | 1.05(0.84,1.26) |
| Uganda | 19022.50(13291.10,29387.38) | 319.86(223.48,494.14) |  | 70066.58(52317.03,96917.08) | 437.92(326.98,605.73) |  | 268.34(163.79,413.87) | 0.48(0.25,0.70) |
| Ukraine | 49082.00(38072.12,61864.83) | 194.81(151.11,245.55) |  | 71000.60(50611.58,94689.83) | 337.22(240.38,449.74) |  | 44.66(27.22,60.85) | 1.10(0.85,1.35) |
| United Arab Emirates | 2816.73(2113.29,3621.51) | 254.28(190.78,326.93) |  | 45112.01(32027.51,60892.54) | 624.99(443.72,843.62) |  | 1501.58(1090.17,1840.16) | 2.05(1.56,2.54) |
| United Kingdom | 53635.87(39774.06,70147.89) | 194.25(144.04,254.05) |  | 172092.86(116549.29,238934.75) | 552.23(373.99,766.71) |  | 220.85(186.70,246.60) | 3.26(3.10,3.43) |
| United Republic of Tanzania | 30186.40(25197.76,36757.10) | 331.35(276.59,403.48) |  | 96181.46(73431.30,122855.22) | 409.85(312.91,523.51) |  | 218.63(132.33,320.32) | 0.59(0.54,0.64) |
| United States of America | 438.65(348.05,564.34) | 870.69(690.86,1120.19) |  | 576.80(420.65,782.10) | 1625.12(1185.15,2203.53) |  | 31.50(10.12,52.30) | 2.07(1.90,2.25) |
| United States Virgin Islands | 347709.58(289050.30,415179.46) | 272.53(226.55,325.41) |  | 905522.88(690363.39,1163951.91) | 599.35(456.94,770.40) |  | 160.43(136.31,183.85) | 2.43(2.16,2.71) |
| Uruguay | 2758.82(2282.99,3354.38) | 198.82(164.52,241.73) |  | 5973.19(4439.80,7896.08) | 373.79(277.84,494.12) |  | 116.51(90.83,141.42) | 2.12(2.03,2.20) |
| Uzbekistan | 16910.25(13874.92,20666.48) | 203.07(166.62,248.18) |  | 97092.04(76293.33,126708.64) | 572.00(449.47,746.48) |  | 474.16(407.05,538.82) | 3.09(2.80,3.38) |
| Vanuatu | 580.53(419.93,828.17) | 987.09(714.02,1408.16) |  | 2542.54(2019.40,3158.74) | 1857.65(1475.43,2307.87) |  | 337.97(212.40,493.73) | 1.85(1.79,1.92) |
| Venezuela (Bolivarian Republic of) | 38642.22(32447.04,46583.59) | 473.25(397.38,570.51) |  | 124496.34(98296.19,158910.52) | 984.56(777.36,1256.72) |  | 222.18(174.06,270.25) | 2.03(1.75,2.32) |
| Viet Nam | 68056.50(53451.95,85172.21) | 246.69(193.75,308.73) |  | 232553.72(182178.12,298027.42) | 454.38(355.95,582.31) |  | 241.71(165.53,343.46) | 2.22(2.06,2.39) |
| Yemen | 8264.02(6145.71,11196.26) | 186.78(138.91,253.06) |  | 47232.08(34185.10,64468.86) | 339.25(245.54,463.06) |  | 471.54(369.98,564.42) | 1.05(0.53,1.56) |
| Zambia | 13797.48(11159.04,16492.08) | 497.09(402.04,594.17) |  | 51672.80(39604.17,66115.55) | 647.46(496.24,828.42) |  | 274.51(179.78,385.05) | 0.47(0.33,0.60) |
| Zimbabwe | 8588.70(6906.27,10534.98) | 234.82(188.82,288.03) |  | 35874.84(27409.37,45843.34) | 551.59(421.43,704.86) |  | 317.70(225.54,434.00) | 3.21(2.59,3.84) |
